# Assessing the Role of the Generative Pretrained Transformer (GPT) in Alzheimer’s Disease Management: Comparative Study of Neurologist- and Artificial Intelligence–Generated Responses

**DOI:** 10.2196/51095

**Published:** 2024-10-31

**Authors:** Jiaqi Zeng, Xiaoyi Zou, Shirong Li, Yao Tang, Sisi Teng, Huanhuan Li, Changyu Wang, Yuxuan Wu, Luyao Zhang, Yunheng Zhong, Jialin Liu, Siru Liu

**Affiliations:** 1 West China Medical School Sichuan University Chengdu China; 2 Department of Neurology West China Hospital Sichuan University Chengdu China; 3 Department of Neurology Chengdu Shangjin Nanfu Hospital Chengdu China; 4 Department of Neurology Guizhou Provincial People’s Hospital Guiyang China; 5 Mental Health Center West China Hospital Sichuan University Chengdu China; 6 West China College of Stomatology Sichuan University Chengdu China; 7 Department of Medical Informatics West China Medical School Chengdu China; 8 West China School of Nursing Sichuan University Chengdu China; 9 Department of Otolaryngology-Head and Neck Surgery West China Hospital Sichuan University Chengdu China; 10 Department of Biomedical Informatics Vanderbilt University Medical Center Nashville, TN United States

**Keywords:** Alzheimer's disease, artificial intelligence, AI, large language model, LLM, Generative Pretrained Transformer, GPT, ChatGPT, patient information

## Abstract

**Background:**

Alzheimer’s disease (AD) is a progressive neurodegenerative disorder posing challenges to patients, caregivers, and society. Accessible and accurate information is crucial for effective AD management.

**Objective:**

This study aimed to evaluate the accuracy, comprehensibility, clarity, and usefulness of the Generative Pretrained Transformer’s (GPT) answers concerning the management and caregiving of patients with AD.

**Methods:**

In total, 14 questions related to the prevention, treatment, and care of AD were identified and posed to GPT-3.5 and GPT-4 in Chinese and English, respectively, and 4 respondent neurologists were asked to answer them. We generated 8 sets of responses (total 112) and randomly coded them in answer sheets. Next, 5 evaluator neurologists and 5 family members of patients were asked to rate the 112 responses using separate 5-point Likert scales. We evaluated the quality of the responses using a set of 8 questions rated on a 5-point Likert scale. To gauge comprehensibility and participant satisfaction, we included 3 questions dedicated to each aspect within the same set of 8 questions.

**Results:**

As of April 10, 2023, the 5 evaluator neurologists and 5 family members of patients with AD rated the 112 responses: GPT-3.5: n=28, 25%, responses; GPT-4: n=28, 25%, responses; respondent neurologists: 56 (50%) responses. The top 5 (4.5%) responses rated by evaluator neurologists had 4 (80%) GPT (GPT-3.5+GPT-4) responses and 1 (20%) respondent neurologist’s response. For the top 5 (4.5%) responses rated by patients’ family members, all but the third response were GPT responses. Based on the evaluation by neurologists, the neurologist-generated responses achieved a mean score of 3.9 (SD 0.7), while the GPT-generated responses scored significantly higher (mean 4.4, SD 0.6; *P*<.001). Language and model analyses revealed no significant differences in response quality between the GPT-3.5 and GPT-4 models (GPT-3.5: mean 4.3, SD 0.7; GPT-4: mean 4.4, SD 0.5; *P*=.51). However, English responses outperformed Chinese responses in terms of comprehensibility (Chinese responses: mean 4.1, SD 0.7; English responses: mean 4.6, SD 0.5; *P*=.005) and participant satisfaction (Chinese responses: mean 4.2, SD 0.8; English responses: mean 4.5, SD 0.5; *P*=.04). According to the evaluator neurologists’ review, Chinese responses had a mean score of 4.4 (SD 0.6), whereas English responses had a mean score of 4.5 (SD 0.5; *P*=.002). As for the family members of patients with AD, no significant differences were observed between GPT and neurologists, GPT-3.5 and GPT-4, or Chinese and English responses.

**Conclusions:**

GPT can provide patient education materials on AD for patients, their families and caregivers, nurses, and neurologists. This capability can contribute to the effective health care management of patients with AD, leading to enhanced patient outcomes.

## Introduction

Alzheimer’s disease (AD) is a progressive neurodegenerative disorder that has emerged as a significant health challenge worldwide. AD International reports indicate that the US population aged 65 years and older is predicted to surge from 58 million in 2021 to 88 million by 2050 [1,2]. As the most common form of dementia, AD accounts for 60%-80% of cases and significantly affects patients, caregivers, and society [3-5]. Given the disease’s progressive nature, efficient management of AD and related cognitive decline is crucial for enhancing the life quality of patients and caregivers [6].

Optimally managing AD often requires a multifaceted approach to address the cognitive, functional, and behavioral symptoms associated with the disorder [7]. Current pharmacological interventions, such as cholinesterase inhibitors and *N*-methyl-D-aspartate receptor antagonists, deliver moderate symptomatic relief but fail to halt disease progression [8-10]. Meanwhile, nonpharmacological interventions, such as cognitive stimulation, physical exercise, and caregiver support programs, have shown potential benefits for patients with AD [11-13]. Patients diagnosed with AD and their caregivers frequently grapple with unmet needs and inadequate awareness about managing the disease and its complications. A lack of health literacy among patients with AD and caregivers has been reported [14,15]. The internet can offer valuable health information, but the complex nature of the primary literature and potential misinformation can often lead to more confusion than clarity [16]. Studies show that online resources provided by dementia-focused organizations are often lengthy and complex, demonstrating a scarcity of easily understandable information for this demographic [17].

Improving health literacy and empowering patients and caregivers about AD require the provision of precise, accessible, and holistic resources that cover both pharmacological and nonpharmacological interventions. Customizing these resources to meet the unique needs of individuals and caregivers at different disease stages is vital, given potential variations in management strategies based on AD severity and progression [18,19]. The increasing prevalence of AD poses a significant challenge for patients, caregivers, and society at large. Effective disease management is paramount to enhance the life quality of those affected. By boosting health literacy and providing accessible, precise, and comprehensive AD management and treatment information, we can empower patients and caregivers, which will ultimately lead to better outcomes for those living with the disease [20].

The Generative Pretrained Transformer (GPT), developed by OpenAI, is an advanced natural language processing (NLP) model based on the GPT-3.5 architecture. It has been refined through supervised learning, human feedback, and reinforcement learning techniques [21]. Released on November 30, 2022, GPT has demonstrated potential in various medical applications, such as answering United States Medical Licensing Examination (USMLE) questions [22], generating simplified radiology reports for patients [23], optimizing clinical decision support [24], and several other clinical applications [25]. However, concerns exist regarding GPT’s comprehension of queries and its restricted capacity to deliver detailed answers [26]. To date, there has been no research evaluating the accuracy and comprehensiveness of GPT in answering specific questions related to AD. Therefore, we collected the perception of participants (evaluator neurologists/family members) regarding the accuracy, comprehensibility, clarity, and usefulness of GPT‘s responses to frequently asked questions related to the management and caregiving of patients with AD.

The aims of this study were:

- To evaluate the potential of GPT‘s capability to respond questions related to AD management
- To compare and assess the difference between GPT’s responses to AD’s care queries and those provided by neurologists

## Methods

### Participant Selection

The recruitment of neurologists was carried out through a purposeful sampling approach, with careful consideration given to ensuring the representativeness of the selected neurologists. Factors considered included their affiliation with different hospitals, years of clinical experience, medical specialties, and age. The selection criteria for neurologists were as follows: (1) ≥5 years of clinical experience in neurology, (2) a medical doctor degree, (3) grade III hospitals (the highest level of hospitals in China), and (4) voluntary willingness to participate in this study.

In the selection of family members to participate in the study, our aim was to ensure diversity and representativeness by including individuals of varying ages, sexes, cultural backgrounds, and educational levels who have experience in caring for a person with AD daily. The criteria for selecting these participants were as follows: (1) 18-55 years old, (2) living with or providing daily care of a person with AD for a minimum of 1 year, (3) proficiency in the language skills necessary to comprehend and evaluate the study’s content, (4) primary school education or higher, and (5) voluntary willingness to participate in this study.

### Questionnaire Development

In our initial phase, we systematically compiled frequently encountered inquiries pertaining to AD from authoritative publications issued by esteemed professional associations and organizations [27-35]. A study showed that most of the content of the educational materials provided by these associations is unanimously recognized, with a mean overall score of 32.33 (SD 4.66) for the applicability of their educational content out of a total score of 44 [36]. This indicates that the content is well suited for health education purposes. To foster inclusivity and encompass a broad spectrum of patients and caregivers, we expanded the initial question pool. We meticulously excluded queries that were redundant, had ambiguous connotations, displayed subjectivity susceptible to individual interpretation, and underwent precise linguistic and grammatical refinement to enhance precision. Subsequently, after 3 rounds of expert discussion, we definitively curated a comprehensive set of 14 questions related to AD. These questions comprehensively address the multifaceted concerns of patients’ families and caregivers, encompassing vital subjects, such as general knowledge regarding AD, associated risks, diagnostic processes, treatment modalities, caregiving strategies, prognostic outlook, rehabilitation measures, and nursing practices (Multimedia Appendix 1). All questions used in this study are listed in Table S1 in Multimedia Appendix 2.

### GPT and Response Generation

GPT (GPT-3.5, or ChatGPT) leverages reinforcement learning from human feedback or reinforcement learning from human preference approaches to generate coherent and contextually appropriate answers. Building on the foundation of the GPT-3.5 large language model, this research preview prototype demonstrates impressive capabilities in understanding and generating human-like text [36]. It underwent pretraining on an extensive data set comprising text extracted from online sources, such as websites, books, and papers up until 2021.

**Figure 1:**
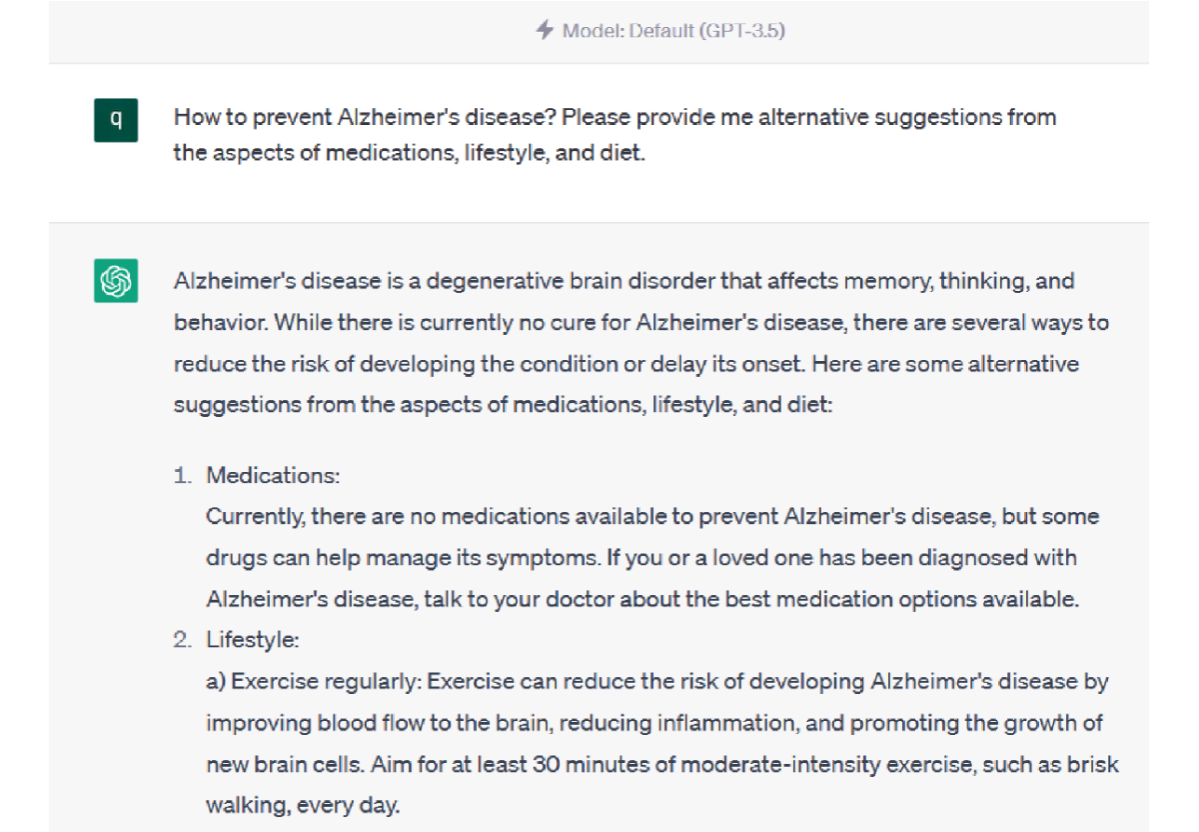
Example of using GPT-3.5 to generate a response. GPT: Generative Pretrained Transformer.

Questions were input into the GPT May 3 version, using the GPT-3.5 and GPT-4 models. Each question was treated as an individual prompt using the “New Chat” function. Additionally, to explore the variations in GPT’s responses, each question was translated into Chinese. Figure 1 shows an example of using GPT-3.5 to generate responses. We also invited 4 respondent neurologists to answer each question to compare the quality of GPT and respondent neurologists’ responses. All responses are listed in Table S2 in Multimedia Appendix 2.

### Evaluation Response

To minimize bias between the evaluators, comprising neurologists and patients’ family members, in their perception of GPT and the responses from respondent neurologists, we eliminated any mentions of artificial intelligence (AI) in the GPT-generated responses. Subsequently, we used a random number generator to generate random identifiers for 8 answers. The coder remained unaware of whether these identifiers were generated by a neurologist or by GPT, ensuring a double-blind approach. The randomly coded answers were independently reviewed and scored by 5 evaluator neurologists and 5 family members of patients for each response. The evaluator neurologists were asked to provide their demographic information, including sex, age, professional title, and years of practice. Each response was assessed using a 5-point Likert scale ranging from 1 (strongly disagree) to 5 (strongly agree), with criteria including accuracy, comprehensiveness, comprehensibility, and overall satisfaction [25,37-40]. Table S3 in Multimedia Appendix 2 shows the questionnaire items for the evaluator neurologists. To further assess the quality of the responses, optional open-ended questions were included, and the evaluator neurologists were asked to explain their scoring choices. The evaluator neurologists were also asked to select the most satisfactory and least satisfactory responses. Patients’ family members were asked to provide their demographic information, including sex, age, occupation, marital status, duration of illness, stage of illness, annual household income, and education level. Each response provided was rated on a 5-point Likert scale ranging from 1 (strongly disagree) to 5 (strongly agree), focusing on criteria such as comprehensibility, usefulness, and overall satisfaction. Table S4 in Multimedia Appendix 2 details the questionnaire items used for the patients’ family members. Furthermore, patients’ family members were asked to select the most satisfactory and least satisfactory response from each set of responses. In this study, we implemented several measures to ensure that all participants, including patients’ family members, could fully comprehend the English responses generated by GPT. All English questionnaires and responses were carefully translated into Chinese by qualified professionals and underwent a rigorous review process to ensure linguistic accuracy and cultural relevance. This translation process was aimed at minimizing any language barriers for the participants (Multimedia Appendix 3).

### Statistical Analysis

Descriptive statistics were calculated for the Likert scale ratings of each response category, including means (SDs). We evaluated the distribution of continuous variables using the Shapiro-Wilk test with IBM SPSS Statistics version 26.0, which indicated nonnormal distribution (Multimedia Appendices 4 and 5). To compare the ratings of responses produced by GPT and those generated by neurologists, we conducted the Mann-Whitney *U* test. The statistical significance level was set at *P*<.05. Furthermore, we calculated intraclass correlation coefficients (ICCs) to assess the reliability among evaluators. The ICC estimates and 95% CIs were calculated with a 2-way mixed-effects model using both the mean and consistency definitions for the k rater type. The ICC values were interpreted as follows: values<0.5 indicate low reliability, 0.5-0.74 indicate moderate reliability, 0.75-0.9 indicate good reliability, and >0.9 indicate excellent reliability [41]. All questionnaires were input into IBM SPSS Statistics version 26.0 for processing. We used Microsoft Excel version 2304 to rank the satisfaction of each response; stacked bar charts were generated by Microsoft Excel version 2304.

### Ethical Considerations

All participants in this study completed the questionnaire voluntarily to ensure equal participation. The data collected do not contain any personally identifiable information, as all information was anonymized to comply with data protection regulations. According to Article 32 of the Regulations on Ethical Review of Biomedical Research Involving Human Subjects in China [42], this study was exempt from the requirement to obtain approval from an ethical review board.

## Results

### Study Participants

As of April 10, 2023, we enrolled 9 neurologists, 4 respondent neurologists, and 5 evaluator neurologists in the study. The respondent neurologists included 2 (50%) neurology specialists, 1 (25%) fellow in neurology, and 1 (35%) neurologist, while the evaluator neurologists included 1 (20%) neurology professor, 1 (20%) neurology specialist, 2 (40%) fellows in neurology, and 1 (20%) neurologist. The mean age of the respondent neurologists and evaluator neurologists was 38.50 (SD 10.91) and 43.80 (SD 16.83) years, respectively. The mean clinical experience of the respondent neurologists and evaluator neurologists was 12.75 (SD 8.81) and 19.60 (SD 16.83) years, respectively. The ICC value of the respondent neurologists and evaluator neurologists was 0.95 (95% CI 0.42-0.99) and 0.98 (95% CI 0.85-0.99), respectively, indicating good reliability. Table 1 shows the characteristics of the respondent neurologists and evaluator neurologists in the study.

As of April 2023, we invited 5 family members of patients with AD to participate in this study. Most participants were female (n=4, 80%), with a mean age of 31.20 (SD 14.87) years. In terms of occupation, 3 (60%) participants were students, 1 (20%) was employed, and the remaining 1 (20%) was self-employed. Their annual family income was concentrated (n=4, 80%) in the range of Chinese Yuan (CNY) 10,000-20,000 (~US $1406-$2813), and the majority of them (n=4, 80%) lived in urban areas. In addition, their educational background ranged from primary school to a master’s degree. Regarding the stage of AD, 4 (80%) were in the middle stage and 1 (20%) in the late stage. The mean duration of AD was 7.0 (SD 4.9) years. The ICC value was 0.89 (95% CI 0.48-0.99), also showing good reliability. Table 2 shows the characteristics of the patients’ family members who participated in the survey.

**Table 1:** Characteristics of neurologists participating in the study.

Characteristics			Respondent neurologists (n=4)		Evaluator neurologists (n=5)
**Sex, n (%)**					
	Male	2 (50)		3 (60)	
	Female	2 (50)		2 (40)	
**Age (years), mean (SD)**			38.50 (10.91)		43.80 (16.83)
**Title of public health technician, n (%)**					
	Neurology professor	0		1 (20)	
	Neurology specialist	2 (50)		1 (20)	
	Fellow in neurology	1 (25)		2 (40)	
	Neurologist	1 (25)		1 (20)	
**Clinical experience (years), mean (SD)**			12.75 (8.81)		19.60 (16.83)
**ICC^a^ (95% CI)**			0.95 (0.42-0.99)		0.98 (0.85-0.99)

^a^ICC: intraclass correlation coefficient.

**Table 2:** Characteristics of patients’ family members (n=5).

Characteristics		Value
**Sex, n (%)**		
	Male	1 (20)
	Female	4 (80)
**Age group (years), n (%)**		
	18-29	3 (60)
	30-50	1 (20)
	51-52	1 (20)
**Occupation, n (%)**		
	Student	3 (60)
	Customer service	1 (20)
	Other	1 (20)
**Marital status, n (%)**		
	Married	3 (60)
	Unmarried	2 (40)
**Living place, n (%)**		
	Urban	4 (80)
	Rural	1 (20)
**Education, n (%)**		
	Primary school diploma	1 (20)
	High school diploma	1 (20)
	Bachelor’s degree	1 (20)
	Master’s degree	2 (40)
**Household income (CNY^a^ per annum), n (%)**		
	10,000-20,000 (~US $1406-$2813)	4 (80)
	20,000-50,000 (~US $2813-$7032)	1 (20)
**Stage of AD^b^, n (%)**		
	Middle stage	4 (80)
	Late stage	1 (20)
**Duration of AD (years), mean (SD)**		7.0 (4.9)
**ICC^c^ value (95% CI)**		0.89 (0.48-0.99)

^a^CNY: Chinese Yuan. An exchange rate of CNY 1= US $0.14 was applied.

^b^AD: Alzheimer’s disease.

^c^ICC: intraclass correlation coefficient.

### Comparison of GPT Responses with Neurologist Responses

A total of 112 questions were answered by GPT and the respondent neurologists. Specifically, GPT-3.5 and GPT-4 each provided responses to 14 questions, both in English and in Chinese. Additionally, the 4 respondent neurologists individually answered 14 questions in a manner consistent with GPT’s responses. All responses are included in the questionnaire (Multimedia Appendix 2). The average length of GPT responses to each question was 668.9 (SD 218.9) characters, of which GPT-3.5 responses had an average length of 630.9 (SD 212.7) characters, GPT-4 responses had an average length of 708.2 (SD 222.2) characters, and the average length of the respondent neurologists’ responses to each question was 577.6 (SD 717.3) characters. In the evaluation of the most satisfactory responses for each questionnaire, the top 4 (80%) of the top 5 responses rated by the respondent neurologists were GPT responses and the fifth was a respondent neurologist’ response (Multimedia Appendix 1). The top 5 responses with the highest scores rated by the patients’ family members were GPT responses (n=4, 80%), except for the third response to a respondent neurologist’s answer (Multimedia Appendix 2). Overall, GPT’s responses received higher satisfaction scores from both evaluator neurologists and patients’ families compared to those from respondent neurologists.

### Results of Evaluator Neurologists’ Review of GPT- and Neurologist-Generated Responses

**Figure 2:**
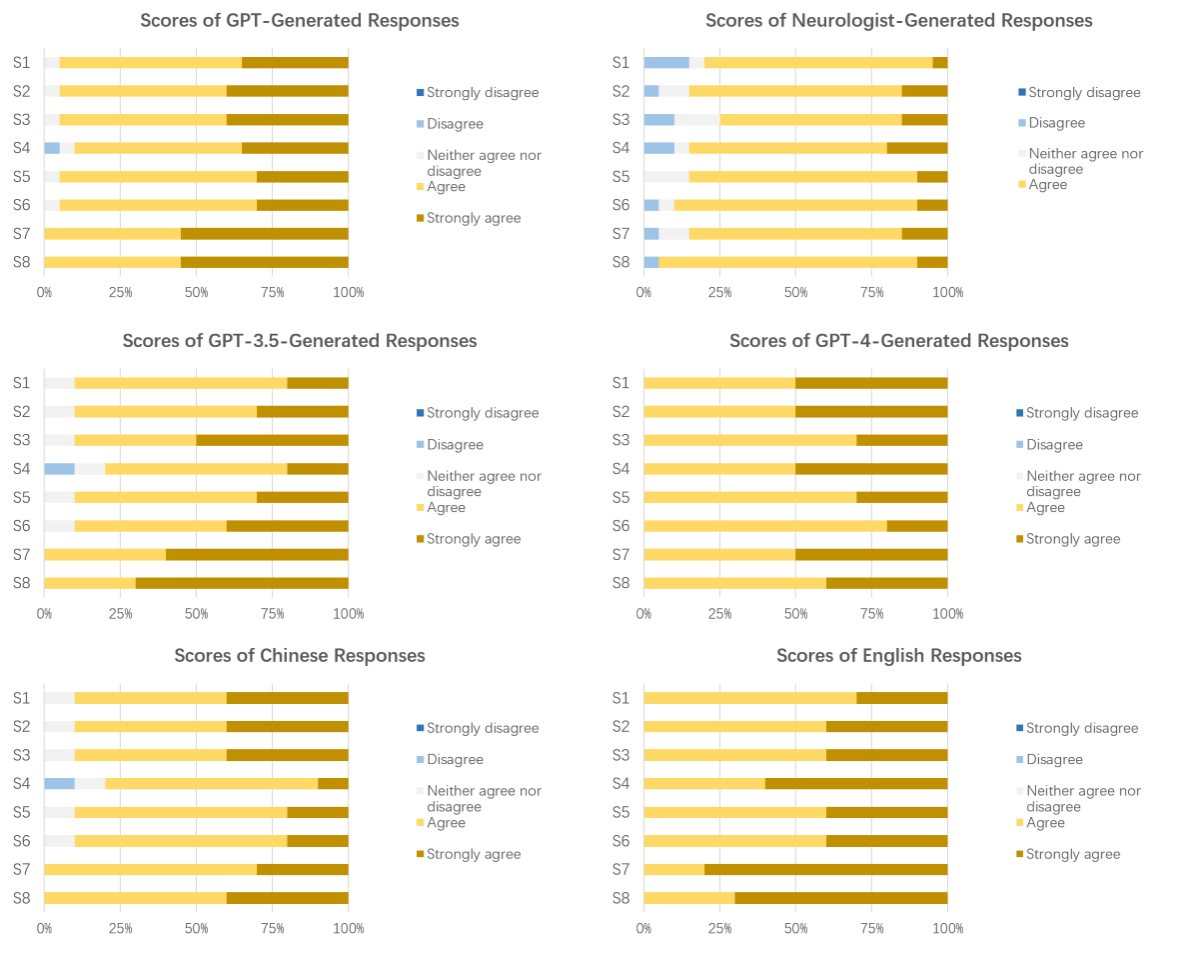
Stacked bar charts of neurologists’ scores of each item for GPT (GPT-3.5+GPT-4)–generated responses and neurologist-generated responses, GPT-3.5- and GPT-4-generated responses, Chinese responses, and English responses. GPT: Generative Pretrained Transformer; S1: accuracy; S2: comprehensiveness; S3, S4, S7: comprehensibility; S5, S6, S8: satisfaction.

In the evaluator neurologists’ evaluations, on average, both GPT- and neurologist-generated responses were rated as “agree” in comprehensiveness and satisfaction. In terms of accuracy and comprehensibility, GPT-generated responses were rated as “agree,” while neurologist-generated responses were rated as “neither agree nor disagree.” Figure 2 shows the stacked bar graphs representing the scores of each item of the GPT- and neurologist-generated responses. The detailed information for each item is listed in Table S3 in Multimedia Appendix 2.

Compared to GPT-generated responses, neurologist-generated responses scored lower in accuracy (GPT: mean 4.3, SD 0.6; neurologists: mean 3.7, SD 0.8; *P*=.04), comprehensiveness (GPT: mean 4.4, SD 0.6; neurologists: mean 4.0, SD 0.7; *P*=.11), comprehensibility (GPT: mean 4.4, SD 0.6; neurologists: mean 3.9, SD 0.8; *P*<.001), and satisfaction (GPT: mean 4.4, SD 0.6; neurologists: mean 4.0, SD 0.6; *P*<.001). The mean overall score for neurologist-generated responses was 3.9 (SD 0.7), while that for GPT-generated responses was 4.4 (SD 0.6; *P*<.001). Table 3 shows the means (SDs) for each aspect, and Table S5 in Multimedia Appendix 2 shows the means (SDs) for each item.

**Table 3:** Evaluator neurologists’ ratings.

Group and response category			Accuracy		Comprehensiveness		Comprehensibility		Satisfaction		Overall
**Group 1**											
	GPT^a,b^-generated responses, mean (SD)	4.3 (0.6)		4.4 (0.6)		4.4 (0.6)		4.4 (0.6)		4.4 (0.6)	
	Neurologist-generated responses, mean (SD)	3.7 (0.8)		4.0 (0.7)		3.9 (0.8)		4.0 (0.6)		3.9 (0.7)	
	*P* value	.04		.11		<.001		<.001		<.001	
**Group 2**											
	GPT-3.5-generated responses, mean (SD)	4.1 (0.6)		4.2 (0.6)		4.3 (0.7)		4.4 (0.9)		4.3 (0.7)	
	GPT-4-generated responses, mean (SD)	4.5 (0.5)		4.5 (0.5)		4.4 (0.5)		4.3 (0.5)		4.4 (0.5)	
	*P* value	.19		.35		.67		.36		.51	
**Group 3**											
	GPT^b^ Chinese responses, mean (SD)	4.3 (0.7)		4.3 (0.7)		4.1 (0.7)		4.2 (0.8)		4.4 (0.6)	
	GPT^b^ English responses, mean (SD)	4.3 (0.5)		4.4 (0.5)		4.6 (0.5)		4.5 (0.5)		4.5 (0.5)	
	*P* value	.91		.85		.005		.04		.002	

^a^GPT: Generative Pretrained Transformer.

^b^GPT-3.5+GPT-4.

To further analyze the responses generated by GPT, we divided the responses into 2 groups according to the model that generated each response and the language of the response. We further compared the scores of each aspect (Table 3) and the scores of the items of the 2 groups of responses generated by GPT. Figure 2 shows stacked bar charts for the scores of each item. The results indicated that there were no statistically significant differences between GPT-3.5 and GPT-4 in terms of accuracy, comprehensiveness, comprehensibility, and satisfaction. However, although there were no significant differences in accuracy and comprehensiveness scores between Chinese and English responses, there were significant differences in comprehensibility (Chinese: mean 4.1, SD 0.7; English: mean 4.6, SD 0.5; *P*=.005) and satisfaction (Chinese: mean 4.2, SD 0.8; English: mean 4.5, SD 0.5; *P*=.04) scores. The mean total score for Chinese responses was 4.4 (SD 0.6), while that for English responses was 4.5 (SD 0.5; *P*=.002). This suggests that GPT performs differently in different languages, with responses in the English context being more comprehensible and satisfying.

### Results of Patient Families’ Review of GPT- and Neurologist-Generated Responses

**Figure 3:**
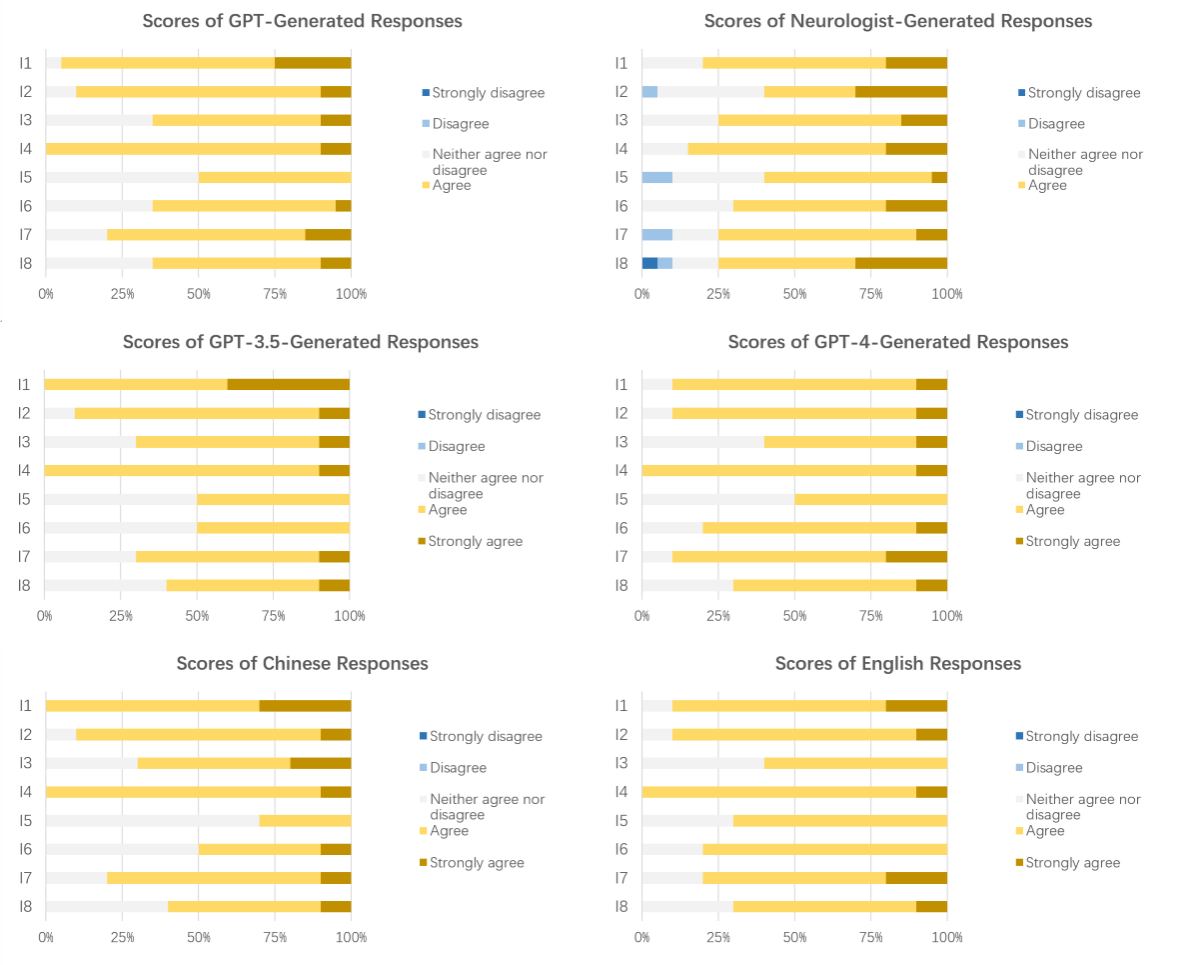
Stacked bar charts of patient family scores for each item for GPT- and neurologist-generated responses, GPT-3.5- and GPT-4-generated responses, Chinese responses, and English responses. GPT: Generative Pretrained Transformer; I1-I3: comprehensibility; I4, I6: practicality; I5, I7, I8: satisfaction.

In patient family ratings, on average, the comprehensibility of GPT-generated responses was rated as “agree,” while practicality and satisfaction were rated as “neither agree nor disagree.” Figure 3 shows stacked bar graphs representing the scores of each item of GPT- and neurologist-generated responses. The neurologist-generated responses scored similarly to the GPT-generated responses in terms of comprehensibility, usefulness, and satisfaction. The total scores for both were the same, 3.9. The means (SDs) for each aspect are shown in Table 4, the detailed information for each item is listed in Table S4 in Multimedia Appendix 2, and the means (SDs) for each item are shown in Table S8 in Multimedia Appendix 2.

As mentioned before, we divided the responses into 2 groups and compared the scores of each aspect (Table 4) and the scores of the items of the 2 groups of responses generated by GPT. We found that there were no significant differences in the comprehensibility, usefulness, and satisfaction scores between GPT-3.5 and GPT-4 and between Chinese and English responses.

**Table 4:** Patients’ family members’ ratings.

Group and response category		Comprehensibility	Usefulness	Satisfaction	Overall
**Group 1**					
	GPT^a,b^-generated responses, mean (SD)	4.0 (0.5)	3.9 (0.5)	3.7 (0.6)	3.9 (0.6)
	Neurologist-generated responses, mean (SD)	3.9 (0.7)	4.0 (0.7)	3.7 (0.9)	3.9 (0.8)
	*P* value	.62	.60	.57	.75
**Group 2**					
	GPT-3.5-generated responses, mean (SD)	4.1 (0.6)	3.8 (0.5)	3.7 (0.6)	3.9 (0.6)
	GPT-4-generated responses, mean (SD)	3.9 (0.5)	4.0 (0.5)	3.8 (0.6)	3.9 (0.6)
	*P* value	.26	.34	.39	.65
**Group 3**					
	GPT^b^ Chinese responses, mean (SD)	4.1 (0.6)	3.9 (0.6)	3.6 (0.6)	3.9 (0.6)
	GPT^b^ English responses, mean (SD)	3.9 (0.5)	4.0 (0.4)	3.8 (0.6)	3.9 (0.5)
	*P* value	.26	.60	.19	.62

^a^GPT: Generative Pretrained Transformer.

^b^GPT-3.5+ GPT-4.

### Comments on GPT- and Neurologist-Generated Responses

In this study, the comments of the 5 evaluator neurologists on all responses were qualitatively analyzed. According to the evaluator neurologists’ comments, the GPT responses had some shortcomings:

- Deviation in understanding the question. For example, 1 of the GPT responses to the question “What are the precautions for medication in AD, and how can medication-related side effects be minimized?” was “diet and lifestyle”, and the advice on care and minimizing medication-related side effects was confusing.
- Omission of information. Some evaluator neurologists commented that the responses generated by GPT were incomplete. For example, in response to the question “How can I prevent AD? Please give me advice on alternative medications, lifestyle, and diet,” the list of medications was incomplete.

There were more problems with the responses generated by the 4 respondent neurologists than with those generated by GPT. Problems found with respondent neurologists’ responses included:

- Missing information. For example, for the question “How can I prevent AD? Please give me alternative advice on medication, lifestyle, and diet,” 1 (25%) respondent neurologist did not provide a comprehensive list of risk factors.
- Template responses. For example, 2 (50%) respondent neurologists provided overly structured answers to the questions “What is the role of regular physical examinations and health assessments in preventing AD?” and “What are the causes or risk factors for AD?”
- Excessive use of medical jargon, for example, in responses to the questions “What is the role of regular health checks and health assessments in preventing AD?” and “What symptoms should a person with AD look for and go to the emergency room for?”
- Lack of relevance. For example, for the question “What are the precautions for medication in AD, and how can medication-related side effects be minimized?” 1 (25%) respondent neurologist did not mention ways to avoid medication-related side effects.
- Lack of practicality. For example, the response of 1 (25%) respondent neurologist to the question “What precautions should I take when treating people with AD with medication, and how can I minimize adverse effects related to medication?” was not practical.
- Potential for ambiguity. For example, when asked, “How do you determine the stage of progression of AD in a patient?”, 1 (25%) respondent neurologist responded that there are 3 stages, with a total of 12 years, which could lead to confusion about the patient’s life expectancy.

These results suggest that although GPT may suffer from misunderstanding, a lack of information, and a lack of relevance, its overall rating is still better than the individual responses of respondent neurologists.

## Discussion

### Principal Findings

The main finding of this research highlights the exceptional performance of GPT responses compared to those provided by evaluator neurologists, showing a significant advantage across 4 key dimensions: accuracy, comprehensiveness, comprehensibility, and overall satisfaction (Table 3). Although neurologists’ responses still performed well in all aspects, they were outperformed by those of GPT. Additionally, a qualitative analysis revealed certain limitations in both GPT’s and neurologists’ responses, with more shortcomings identified in the neurologists’ responses. Qualitative analysis can be influenced by factors such as the specific questions asked, the expertise of the neurologist, and the criteria used to score the responses. These findings align with prior research indicating the superior performance of GPT compared to human professionals in various medical specialties, particularly in cardiology, interventional radiology, and ophthalmology [43-45]. However, the application of GPT in a clinical setting must be approached with care, emphasizing ethics and transparency to safeguard patients’ rights and the quality of health care [25,46].

The superior performance of GPT responses compared to those of neurologists can be attributed to GPT’s remarkable potential for comprehensive data coverage [47]. GPT excels in effectively covering a wide spectrum of medical literature, allowing it to provide information that spans different medical domains [48]. In contrast, neurologists tend to have expertise focused on specific medical specialties or in dealing with individual patient histories, which tends to limit their scope of response [49]. The discrepancies observed between the responses of GPT and those of neurologists can be attributed to various factors affecting the latter. These include time constraints, the depth of expertise, and the variability inherent in individual knowledge and experience. Conversely, the limitations in GPT’s performance may be due to deficiencies in training data, which may include inaccuracies and omissions [43,50]. Moreover, as an AI language model, GPT may not fully capture and communicate the contextual nuances and emotional subtleties that are often essential in medical consultations [51,52]. A remarkable limitation is its interaction style: When faced with ambiguous queries, GPT lacks the capability to request clarification through follow-up questions, such as “Did you mean home care services?” [50]. Furthermore, several studies have indicated that the responses generated by GPT may present readability challenges for a general audience, potentially impacting their comprehensibility and accessibility [50,53-56]. Therefore, it is important to maintain a balanced perspective when evaluating the effectiveness of AI models like GPT in clinical settings. This involves acknowledging their strengths in terms of data coverage and information retrieval, while also being aware of their limitations in areas where human practitioners outperform them.

In the comparative analysis, GPT-4 (score range 4.2-4.5) outperformed GPT-3.5 (score range 3.9-4.7). However, this difference did not have statistical significance (*P*>.05), indicating that the improvements in GPT-4 may not be significant enough to substantially improve the quality of responses to AD-related queries. Nevertheless, GPT-4 has displayed superior performance in various domains, especially in the USMLE and StatPearls questions related to ophthalmology, epilepsy, and patient education. GPT-4 has statistically significantly outperformed GPT-3.5 (*P*<.001 to <.005) [45,57,58]. Additionally, it has proven to be effective in delivering accurate and comprehensible information to patients about medical procedures, associated risks, benefits, and recovery periods, thereby supporting informed decision-making [59]. OpenAI reports that GPT-4 is “82% less likely to respond to requests for disallowed content and 40% more likely to produce factual responses than GPT-3.5 on our internal evaluations” [60]. Moreover, GPT-4 benefits from training on more recent data, extending up to September 2021, including up-to-date information, unlike GPT-3.5, which was limited to data available before June 2021. This enhancement allows GPT-4 to generate more up-to-date responses [61]. There has been a notable increase in the consistency of GPT-4’s responses over time, possibly due to its robust training and advanced sampling mechanisms that promote response stability [62]. With continued advances in AI, the GPT-4 is poised to become a valuable tool for patient management and health care delivery.

The comparison analysis showed that the responses generated by the GPT model in English (score range 4.3-4.7) significantly outperformed those in Chinese (score range 3.8-4.5; *P*<.05). This difference in performance suggests more advanced training and proficiency of the GPT model in English language contexts, indicating that responses to AD-related consultations may be more effective in English. Additionally, Hristidis et al’s [50] research supported the limited capability of the GPT model in languages other than English, attributing this to a relatively lower level of training and development in non-English languages. Conversely, Takagi et al [57] provided evidence of GPT-4’s effectiveness in the areas of clinical reasoning and medical knowledge within the Japanese language context. This finding provides an important perspective on the potential of GPT models in different linguistic environments, emphasizing the necessity for more extensive training and development in languages other than English to achieve optimal performance in a wider range of linguistic settings.

To better evaluate the usefulness of the GPT model’s responses, we compared the responses of the GPT model to patients’ family members with those provided by the neurologists. The focus was on comprehensibility, usefulness, and overall satisfaction with the patient’s actual care needs. The results showed that the responses of the GPT model were similar to those of the neurologists, with a score of 3.9, and the difference was not statistically significant (*P*>.05). This suggests that the similarity in scores may be due to the limited medical expertise of the patients’ families. Their ability to recognize the professional depth of the responses may be limited, leading them to prioritize the usefulness and understandability of the information over its medical accuracy and complexity [58,63,64]. This observation emphasizes a crucial aspect of using AI in patient care communication: the need to balance professional medical advice with lay understandability and applicability.

### Limitations

There are several limitations of our study that need to be discussed. First, the sample size used for assessment was relatively small, consisting of only 5 neurologists and 5 family members of patients with AD as raters. Although we carefully considered the representativeness of both neurologists and family members when selecting them, the limited sample size could introduce potential biases into the results. Factors such as the clinical experience of the selected neurologists, the time constraints they faced, and the educational background and medical knowledge of the patients’ family members may have influenced the outcomes. Consequently, the results of this study may not be fully reflective of the broader population of neurologists and patients’ families. Second, it is important to note that GPT was trained on text data only up to 2021 and does not include information on drugs, clinical guidelines, or research developments beyond that year. Consequently, GPT’s responses may contain outdated clinical recommendations, which could impact the accuracy and relevance of its suggestions. Furthermore, GPT has the capability to generate responses that sound confident but may not necessarily be accurate, including the possibility of providing incorrect answers or fabricated references [65-67]. Finally, it is essential to acknowledge that the assessment process relies on the perceptions of the participants, specifically the assessors who are neuroscientists and family members. Although we have developed normative assessment criteria, these evaluations inherently involve subjective judgments, introducing a degree of subjectivity to the assessment process. These limitations emphasize the need for caution in interpreting our findings and the importance of further research to address these limitations. Such research will contribute to a more comprehensive understanding of the potential and limitations of GPT in health care settings.

### Conclusion

The results of this study suggest that GPT is a promising tool in the management of AD. The responses provided by GPT can be a valuable resource, providing useful information and support to patients and their families. However, it is important to emphasize that GPT should be used judiciously and should not be relied upon as the sole source of knowledge and expertise. Neurologists should continue to work with GPT, using their clinical expertise and judgment to ensure that patients receive accurate and tailored treatment recommendations.

## Appendix

Multimedia Appendix 1 Questionnaire development.

Multimedia Appendix 2 Input prompts and AI outputs. AI: artificial intelligence.

Multimedia Appendix 3 Translation development process.

Multimedia Appendix 4 Normal distribution test of evaluator neurologists’ reviews.

Multimedia Appendix 5 Normal distribution test of patients' family members' reviews.

Multimedia Appendix 6 Original GPT transcripts. GPT: Generative Pretrained Transformer.

## Abbreviations

AD: Alzheimer's disease
AI: artificial intelligence
CNY: Chinese Yuan
GPT: Generative Pretrained Transformer
ICC: intraclass correlation coefficients
NLP: natural language processing
USMLE: States Medical Licensing Examination

## Data Availability

The input prompts and artificial intelligence outputs are available in Multimedia Appendix 2.
