# Supplementary material for: Assessing the Role of the Generative Pretrained Transformer (GPT) in Alzheimer’s Disease Management: Comparative Study of Neurologist- and Artificial Intelligence–Generated Responses
Source: J Med Internet Res. 2024 Oct 31;26:e51095. doi: 10.2196/51095 (PMC11565080; doi:10.2196/51095)
Supplement: Multimedia Appendix 1 [file jmir_v26i1e51095_app1.docx]

**Questionnaire Development**

This study, conducted between September 2022 and November 2022, utilized a modified Delphi consultation method for the development of a questionnaire. The methodology can be outlined as follows:

**Content Collection and Categorization**

The initial step involved gathering content related to patient education from nine globally recognized Alzheimer's disease websites.

This content was systematically categorized into seven key topics, which encompassed:

1. General knowledge of Alzheimer's Disease (AD);
2. Associated risks;
3. Diagnostic process;
4. Treatment modalities;
5. Caregiving strategies;
6. Prognostic outlook;
7. Rehabilitative measures and care practices. A total of 60 pertinent questions were collated during this phase.

**Expert Involvement and Interviews**

To ensure the questionnaire's robustness, interviews were conducted with a panel of experts comprising: Five neurology experts; Two geriatric nurses; Two family members of AD patients. These one-to-one interviews, lasting 30-60 minutes each, consisted of the following inquiries:

(1) Do these 60 questions adequately encompass the fundamental topics that AD patients and their families need to be informed about? If not, please suggest additional questions.

(2) Can you identify similarities among the 60 questions?

(3) Please list the questions in accordance with the seven critical topics. For each of the seven essential topics, two questions were presented. Subsequently, based on expert feedback, 25 questions were identified as salient.

**Refinement and Finalization**

The research team engaged in extensive discussions to evaluate and select the most pertinent questions. Fourteen questions were ultimately finalized through a consensus-building process within the team.

**Expert Validation**

To validate the selected 14 questions, they were returned to the panel of experts.

The experts were tasked with assessing whether these questions adequately covered the essential information that AD patients and their families needed, as well as ensuring clarity and absence of ambiguity in the question formulation. The final 14 questions were determined following this expert validation.

In summary, this study employed a rigorous and systematic approach to develop a questionnaire for AD, involving content extraction, expert input, and validation procedures to ensure the reliability and relevance of the final questionnaire.
