# Supplementary material for: Assessing the Role of the Generative Pretrained Transformer (GPT) in Alzheimer’s Disease Management: Comparative Study of Neurologist- and Artificial Intelligence–Generated Responses
Source: J Med Internet Res. 2024 Oct 31;26:e51095. doi: 10.2196/51095 (PMC11565080; doi:10.2196/51095)
Supplement: Multimedia Appendix 2 [file jmir_v26i1e51095_app2.docx]

Table S1. Selected questions.

| **Questions** | |  |
| --- | --- | --- |
| Q1. How to prevent Alzheimer's disease? Please provide me alternative suggestions from the aspects of medications, lifestyle, and diet. | |  |
| Q2. What are the causes or risk factors for Alzheimer's disease? | |  |
| Q3. If a parent had/has Alzheimer's disease, are their children at greater risk of getting it? Is Alzheimer's disease hereditary? | |  |
| Q4. What is the role of regular physical examination and health assessments help the prevention of Alzheimer's disease? | |  |
| Q5. How to prevent and manage memory impairment in patients with Alzheimer's disease? | |  |
| Q6. What are the treatments options for Alzheimer's disease? Please provide me alternative suggestions from the aspects of pharmaceutical, psychosocial, and caregiving. | |  |
| Q7. What considerations are there for pharmacological treatment in patients with Alzheimer's disease, and how can medication-related adverse effects be minimized? | |  |
| Q8. What types of nursing and care are necessary for patients with Alzheimer's disease? | |  |
| Q9. What strategies can be used to provide nutritional support for patients with Alzheimer's disease? | |  |
| Q10. What are the symptoms that Alzheimer's disease patients should look out and go to the emergency room for? | |  |
| Q11. How to help the families of late-stage Alzheimer's disease patients cope with the stress of daily living and caregiving? | |  |
| Q12. How can Alzheimer's disease patients receive psychological and emotional support? | |  |
| Q13. How to determine the stage of Alzheimer's disease progression in a patient? | |  |
| Q14. How to determine the stage of Alzheimer's disease progression in a patient? | |  |
|  |  |  |

Table S2. Responses from the ChatGPT Language Model and neurologist for 14 questions.

| Question 1: How to prevent Alzheimer's disease? Please provide me alternative suggestions from the aspects of medications, lifestyle, and diet. |
| --- |
| GPT3 Chinese version:  Alzheimer's disease is a disease that gradually affects the function of the human brain. Although there is no complete cure for the disease, there are some measures that can help prevent and delay its onset and progression.  Drugs:  While there are currently no medications specifically designed to prevent Alzheimer's disease, some medications may help slow the progression of the disease. For example, some clinical trials have shown that the use of cholinesterase inhibitors (such as donepezil) may help improve memory and cognitive function in patients. In addition, other drugs such as risperidone may also be of some benefit, but should be used only on the advice of a doctor.  In terms of lifestyle:  1. Focus on physical activity: Physical activity is good for both brain and body health. 150 minutes of moderate-intensity aerobic activity per week can improve cardiovascular fitness and cognitive performance.  2. Social interaction: Staying social, making new friends, or participating in community events are also effective ways to improve cognition and prevent Alzheimer's disease.  3. Keep learning: Learning new things, learning new skills, reading, participating in discussions, etc. help keep the brain active and improve memory and cognition.  Diet:  1. Adequate intake of vitamins and minerals: such as vitamin B, vitamin C and folic acid, etc., can be taken through food or supplements. Folate is especially important, a deficiency of which has been linked to an increased risk of Alzheimer's disease.  2. Choose healthy oils: Eat more foods rich in unsaturated fatty acids, such as fish, nuts, olive oil, etc., which help to protect brain health.  Cut back on sugar: Studies show that consuming too much sugar may increase your risk of Alzheimer's disease, so try to minimize your intake of sugary foods.  Preventing Alzheimer's disease requires a multifaceted approach, including medication, lifestyle, and diet. Note, however, that these recommendations don't completely prevent Alzheimer's disease, but they can help reduce your risk of developing the disease. Also, if you have a family history or other high-risk factors, such as high blood pressure, high cholesterol, or diabetes, talk to your doctor early on for more preventive measures and treatment options. Finally, staying positive and reducing stress and anxiety can also help keep your brain healthy. |
| GPT3 English version:  Alzheimer's disease is a degenerative brain disorder that affects memory, thinking, and behavior. While there is currently no cure for Alzheimer's disease, there are several ways to reduce the risk of developing the condition or delay its onset. Here are some alternative suggestions from the aspects of medications, lifestyle, and diet:  Medications:  Currently, there are no medications available to prevent Alzheimer's disease, but some drugs can help manage its symptoms. If you or a loved one has been diagnosed with Alzheimer's disease, talk to your doctor about the best medication options available.  Lifestyle:  a) Exercise regularly: Exercise can reduce the risk of developing Alzheimer's disease by improving blood flow to the brain, reducing inflammation, and promoting the growth of new brain cells. Aim for at least 30 minutes of moderate-intensity exercise, such as brisk walking, every day.  b) Stay mentally active: Keeping your brain active by engaging in activities that challenge you, such as reading, doing puzzles, or learning a new skill, can help reduce the risk of Alzheimer's disease.  c) Manage stress: Chronic stress can increase the risk of developing Alzheimer's disease. Try to manage your stress levels through techniques such as meditation, yoga, or deep breathing exercises.  d) Get enough sleep: Lack of sleep can contribute to cognitive decline and increase the risk of Alzheimer's disease. Aim for at least seven hours of sleep every night.  Diet:  a) Eat a healthy diet: A diet rich in fruits, vegetables, whole grains, lean protein, and healthy fats can help reduce the risk of Alzheimer's disease.  b) Limit alcohol intake: Heavy alcohol consumption can damage the brain and increase the risk of Alzheimer's disease. Try to limit your alcohol intake to no more than one drink per day.  c) Watch your sugar intake: A high-sugar diet can increase inflammation in the brain, which can contribute to cognitive decline. Limit your intake of sugary foods and drinks.  d) Consider supplements: Some supplements, such as omega-3 fatty acids, vitamin D, and vitamin E, may help reduce the risk of Alzheimer's disease. Talk to your doctor before starting any supplement regimen. |
| GPT4 Chinese version:  Alzheimer's disease is a common neurodegenerative disease, mainly manifested by the gradual decline of cognitive function. Although there is currently no cure, medications, lifestyle and dietary adjustments can be used to reduce the risk or delay the course of the disease. Here are some suggestions:  Drugs:  Maintain regular communication with your doctor to keep abreast of the latest research progress and preventive measures.  If there is a family history or other high-risk factors, drug intervention can be taken under the guidance of a doctor.  In terms of lifestyle:  Keep exercising: insist on aerobic exercise, such as walking, running, swimming, etc., at least 150 minutes a week.  Stay socially active: Stay connected with family, friends, and participate in community activities to reduce feelings of loneliness and symptoms of depression.  Maintain mental activity: Do reading, writing, puzzle solving and other activities to enhance the cognitive function of the brain.  Sufficient sleep: Ensure 7-9 hours of high-quality sleep every night, which helps the brain to remove harmful substances.  Diet:  Increasing your intake of vegetables, fruits, and whole grains, which are rich in fiber and antioxidants, can help prevent neurodegenerative diseases.  Increasing foods rich in Omega-3 fatty acids, such as deep-sea fish, nuts, flaxseeds, etc., can help protect brain health.  Reduce the intake of saturated fat, trans fat and salt to reduce the risk of cardiovascular disease.  Eat plenty of antioxidant-rich foods like nuts, berries, and red wine in moderation.  Note that the above recommendations do not guarantee complete prevention of Alzheimer's disease, but can help reduce your risk. |
| GPT4 English version:  There is no guaranteed way to prevent Alzheimer's disease, but there are some strategies that may help reduce the risk. It is important to note that these suggestions are not definitive, but they may contribute to overall brain health and promote cognitive function.  Medications:  Currently, there are no medications specifically designed to prevent Alzheimer's disease. However, some studies suggest that managing blood pressure, cholesterol levels, and other cardiovascular risk factors may contribute to a reduced risk. It is essential to talk to a healthcare professional about any concerns and the potential benefits of medications for these conditions.  Lifestyle:  Physical activity: Engage in regular exercise, as it may help improve brain health by increasing blood flow, reducing inflammation, and promoting the growth of new brain cells. Aim for at least 150 minutes of moderate aerobic activity or 75 minutes of vigorous aerobic activity per week.  Mental stimulation: Keep your brain active through mentally challenging activities like reading, solving puzzles, learning new skills, or playing strategic games. These can help build cognitive reserve and maintain brain function.  Social engagement: Stay socially active by maintaining relationships with friends and family or participating in group activities. Social engagement can help reduce stress and maintain cognitive function.  Sleep: Prioritize getting enough sleep, as poor sleep or sleep disorders can negatively affect brain health. Aim for 7-9 hours of quality sleep per night.  Stress management: Practice stress reduction techniques like meditation, yoga, or deep breathing exercises, as chronic stress can harm brain health.  Diet:  Follow a brain-healthy diet, such as the Mediterranean or DASH (Dietary Approaches to Stop Hypertension) diets. These emphasize fruits, vegetables, whole grains, lean protein, and healthy fats while limiting saturated fats, sugars, and processed foods.  Include omega-3 fatty acids in your diet, which are found in fatty fish like salmon, mackerel, and sardines. Omega-3s may help reduce inflammation and support brain health.  Consume antioxidants, which can be found in foods like berries, leafy greens, and nuts. Antioxidants can help protect brain cells from damage caused by free radicals.  Limit alcohol consumption, as excessive alcohol use can increase the risk of developing Alzheimer's disease. Stick to moderate drinking guidelines (up to one drink per day for women and up to two drinks per day for men).  Remember that these suggestions are not definitive ways to prevent Alzheimer's disease but may help promote overall brain health. Always consult a healthcare professional for personalized advice and recommendations. |
| Neurologist 1:  ① Drugs: Patients need to add some vitamins, such as some vitamin B complex or folic acid, which can prevent cerebral arteriosclerosis, and vitamin E should be added. Because vitamin E can improve the function of brain cells and is an antioxidant.  ②Life style: A. Exercising muscles can stimulate the brain, and at the same time, it is necessary to pay attention to the exercises of balance and coordination. Learn a foreign language, practice a musical instrument, learn to draw, and more. B. The greater the novelty, complexity and challenge of the learning content, the greater the benefit.  ③ Diet: Exercising muscles can stimulate the brain, and at the same time, it is necessary to pay attention to the practice of balance and coordination. Improving balance and coordination as you age can reduce your risk of head injury from falls. |
| Neurologist 2:  Answer: Prevention of Alzheimer's disease-- medicine articles:  Treatment of hypercholesterolemia; treatment of type 2 diabetes; treatment of hypertension and other chronic cardiovascular and cerebrovascular diseases.  Prevention of Alzheimer's Disease - Lifestyle:  (1) To maintain brain health, we must start from a young age, overcome unhealthy lifestyles, stay away from unhealthy living habits, do not stay up late, and ensure adequate sleep. Don't overuse your brain, don't smoke, don't drink too much, stay away from drugs. (2) Regular physical and mental activities, insist on physical exercise, such as walking for 30 minutes a day will make the body and brain full of vitality. (3) Carry out frequent interpersonal communication, retain and develop one's own interests and hobbies. The brain is "use it or lose it", the more you use the brain the better, including chatting with friends, actively thinking about problems, participating in reading, calligraphy, painting, photography, writing, playing games, or keeping a diary, weaving handicrafts, etc. (4) Protect the heart and prevent heart disease, hypertension, diabetes, hyperlipidemia, and stroke. If you already have these chronic diseases, you must actively treat them to control blood pressure, blood sugar, blood lipids, hyperviscosity, weight, atrial fibrillation, arrhythmia, coronary heart disease, cerebrovascular disease, etc., because these are Alzheimer's diseases risk factors. (5) Avoid traumatic brain injury: fasten seat belts when driving; keep the room clean and avoid falling; pay attention to obey the traffic rules when riding bicycles and crossing the road.  Prevention of Alzheimer's Disease - Diet:  Eat a balanced diet with less fat and more foods rich in antioxidants. Keep your weight, blood pressure, cholesterol, and blood sugar levels within normal limits. Eat fruits and vegetables; eat fish at least once a week. Reduce the intake of saturated fatty acids and meat and quit smoking. |
| Neurologist 3:  Drugs: 1. Drugs to improve cognitive function  Drugs that improve cognitive function can improve the cognitive function of patients with Alzheimer's disease in the early stage, and delay the rate of cognitive decline in the later stage. (1) Vasodilators: Papaverine can improve the patient's attention and mood. Cyclomandelate (antithrombotic pills) can also be used. Isosuprin.  Brain metabolism-promoting drug: hydroergot alkaloid (hydergine), which can promote brain cell metabolism, the dose is 3-4·5mg/day, taken in three divided doses. The dose of piracetamide (naofukang) is 1·2-2·4g/day, taken in three divided doses. The dose of pyrithione (brain rejuvenation) is 300-600 mg/day in three divided doses.  2. Drugs to control psychosis  Antidepressant and antipsychotic drugs can be given when patients develop delusions, anxiety, hallucinations, depression, sleep disturbance and other related symptoms. Patients with Alzheimer's disease need to be treated with drugs under the guidance of doctors when early symptoms appear. Although the disease is irreversible and cannot be cured after the clinical symptoms appear, effective treatment can delay the progression of the disease and improve the quality of life of the patient.  Lifestyle: 1) Be careful not to use aluminum cooking utensils. The amount of aluminum in the brain of the elderly with dementia is four times that of the average person, so try to reduce exposure to aluminum;  2) Insist on using the brain, insist on reading, playing chess, guessing puzzles and other mental activities, so that the brain cells are always active and excited, and slow down the aging of brain function;  3) Pay attention to listening to music more, music will have a positive impact on brain waves, blood flow, and hormones, and can stimulate people's various senses, thereby activating brain functions;  4) Do more exercise, such as tapping teeth can increase blood flow to the face and brain, walking or jogging can also provide more energy for the brain, and finger movements can directly stimulate brain cells;  5) Pay attention to quitting smoking and drinking. Toxic substances such as tobacco and alcohol have an impact on the function of the brain;  6) Pay attention to maintaining extensive contact with the society, avoid living in isolation, and actively participate in social activities, which can not only restore mental and physical strength, but also reduce stress levels and help maintain the communication function between brain cells.  Diet: 1) Eat scientifically, adhere to a balanced diet with low fat, low salt, meat and vegetables, which can prevent cerebral arteriosclerosis and reduce the occurrence of dementia.  2) Pay attention to vitamin supplements in the diet, such as vitamin B and folic acid can prevent arteriosclerosis, and vitamin E is an antioxidant that can improve the function of brain cells.  3) Pay attention to eat more vegetables, drink more water, and prevent constipation. Long-term constipation can make people's intelligence decline. Studies have shown that more than 80% of Alzheimer's patients are related to constipation. |
| Neurologist 4:  The ways to prevent Alzheimer's disease are as follows:  In terms of drugs: (1) Use strong drugs with caution and avoid drug abuse; (2) Use drugs correctly under the guidance of doctors and avoid self-medication; (3) Regularly check your physical condition to detect diseases early.  In terms of lifestyle: (1) Participate in more social activities to avoid loneliness and depression; (2) Exercise regularly to maintain mental health; (3) Maintain a positive and optimistic attitude and actively face life pressure.  Diet: (1) Eat more fresh vegetables and fruits, and avoid high-fat and high-sugar foods; (2) Use more vegetable oils to reduce the intake of saturated fatty acids; (3) Control alcohol consumption and avoid excessive alcohol consumption. |

| Question 2: What are the causes or risk factors for Alzheimer's disease? |
| --- |
| GPT3 Chinese version:  Alzheimer's disease is a neurodegenerative disorder whose causes are not fully understood, but a variety of factors may increase the risk. The following are possible causes and risk factors:  1. Age: The risk of developing Alzheimer 's disease increases with age.  2. Genes: There is a genetic predisposition to Alzheimer's disease, and if there is someone with Alzheimer's disease in the family, the risk is higher. Specifically, the early onset of Alzheimer's disease (onset before age 65) is often associated with some genetic mutations.  3. Protein deposition: Deposition of two proteins, β - amyloid protein and tau protein, will appear in the brain of Alzheimer's disease patients. These deposits interfere with communication between neurons and eventually lead to neuron death.  4. Inflammation: Inflammation may negatively affect the brain and accelerate the progression of Alzheimer's disease.  5. Nutrition: Deficiencies in certain nutrients, such as folic acid and vitamins B6 and B12, may increase the risk of Alzheimer's disease.  6. Other diseases: Chronic diseases such as high blood pressure, diabetes and cardiovascular disease may increase the risk of Alzheimer 's disease.  7. Lifestyle: Poor lifestyle habits such as lack of physical activity, high-fat diet, smoking and alcohol consumption may increase the risk of Alzheimer's disease.  It is important to note that the causes and risk factors of Alzheimer's disease are still largely unknown, and there is currently no cure for Alzheimer's disease. |
| GPT3 English version:  Alzheimer's disease is a complex disorder that involves a combination of genetic, environmental, and lifestyle factors. While the exact cause of Alzheimer's disease is not yet fully understood, researchers have identified several risk factors that may increase the likelihood of developing the condition.  Here are some of the most well-known risk factors for Alzheimer's disease:  Age: Alzheimer's disease is more common in people over the age of 65, and the risk increases as you get older.  Genetics: Certain genes have been linked to an increased risk of Alzheimer's disease, including the APOE gene. However, having these genes does not mean that you will necessarily develop the disease.  Family history: People who have a first-degree relative (parent or sibling) with Alzheimer's disease are more likely to develop the condition themselves.  Lifestyle factors: Certain lifestyle factors, such as smoking, high blood pressure, and high cholesterol, have been linked to an increased risk of Alzheimer's disease.  Traumatic brain injury: People who have experienced a severe head injury or repeated concussions may be at a higher risk of developing Alzheimer's disease later in life.  Chronic stress: Prolonged stress and anxiety may also increase the risk of developing Alzheimer's disease.  It's worth noting that while these risk factors can increase the likelihood of developing Alzheimer's disease, they do not guarantee that someone will develop the condition. Additionally, there are also some factors that may help reduce the risk of Alzheimer's disease, such as regular exercise, a healthy diet, and staying socially engaged. |
| GPT4 Chinese version:  Alzheimer's disease is a common neurodegenerative disease characterized by progressive memory loss and cognitive decline. Although the exact cause of Alzheimer's disease is not fully understood, researchers have identified several possible causes and risk factors.  Genetic factors: Some people have genes that may make them more likely to develop Alzheimer's disease. For example, people who carry gene variants such as APOE-e4 are more likely to develop the disease.  Age: Age is the greatest risk factor for Alzheimer's disease. The risk of developing the disease increases with age, especially in those over 65 years of age.  Family history: If someone in your family has Alzheimer's disease, your risk may be increased.  Gender: Women have a slightly higher risk of Alzheimer's disease than men, which may be related to women's longer average life expectancy.  Cardiovascular risk factors: Risk factors for cardiovascular diseases such as high blood pressure, high cholesterol, diabetes, and obesity may be related to the occurrence of Alzheimer's disease.  Traumatic brain injury: Head injuries, especially those that result in loss of consciousness, may increase the risk of Alzheimer's disease.  Lifestyle and environmental factors: Several studies suggest that staying physically, mentally healthy, and socially active may help reduce the risk of Alzheimer's disease. Unhealthy lifestyles such as smoking, lack of exercise, and poor diet may also increase the risk.  Note that the risk factors listed above do not necessarily lead to Alzheimer's disease. These factors only increase the likelihood of disease. In many cases, the onset of Alzheimer's disease may be the result of a combination of factors. Knowing these risk factors can help us take preventive measures to reduce the risk of disease. |
| GPT4 English version:  Alzheimer's disease is a complex and multifaceted neurodegenerative disorder. The exact cause of Alzheimer's disease is not yet fully understood, but researchers believe that a combination of genetic, environmental, and lifestyle factors contribute to the development of the disease. Some known risk factors for Alzheimer's include:  Age: The risk of developing Alzheimer's disease increases with age, with most cases occurring in people over the age of 65. However, early-onset Alzheimer's can also occur in people in their 40s and 50s, though it is less common.  Genetics: Family history and specific gene mutations can increase the risk of developing Alzheimer's. The presence of the APOE-e4 gene is a well-known genetic risk factor, but other genes may also contribute to the disease.  Family history: Having a first-degree relative, such as a parent or sibling, with Alzheimer's disease increases the likelihood of developing the condition.  Sex: Women have a higher risk of developing Alzheimer's disease than men, possibly due to hormonal and genetic differences.  Head injuries: A history of moderate to severe head injuries may increase the risk of Alzheimer's disease.  Cardiovascular risk factors: Conditions that affect the heart and blood vessels, such as high blood pressure, high cholesterol, and diabetes, can increase the risk of Alzheimer's disease. Adopting a heart-healthy lifestyle can help reduce these risk factors.  Lifestyle factors: Poor diet, lack of exercise, smoking, and excessive alcohol consumption may contribute to an increased risk of developing Alzheimer's disease.  Education and cognitive reserve: Lower levels of education and engagement in mentally stimulating activities have been associated with a higher risk of Alzheimer's disease.  Sleep disorders: Chronic sleep problems, such as insomnia or sleep apnea, may increase the risk of developing Alzheimer's disease.  Chronic inflammation and oxidative stress: Inflammation and oxidative stress are thought to contribute to the development of Alzheimer's disease, and factors that increase inflammation and oxidative stress can increase the risk.  It is important to note that having one or more of these risk factors does not guarantee that someone will develop Alzheimer's disease. However, understanding and managing these factors can help reduce the risk and delay the onset of symptoms. |
| Neurologist 1:  1). Etiology: ① Abnormal biochemical transmitters in the central nervous system: the concentration and activity of choline acetyltransferase decrease, resulting in a decrease in the synthesis of acetylcholine in the presynapse of neurons, especially in the hippocampus and temporal lobe cortex is closely related to near memory and instantaneous memory. In severe cases, systems such as dopamine, norepinephrine, and serotonin are also damaged. ② Genetic factors: There is a mild genetic predisposition, and the incidence rate in the immediate relatives of patients is 4 times that of the general population. ③ amyloid deposits in the central nervous system. ④ Accumulation of metal aluminum in the brain: it can lead to blockage of nerve cell protein synthesis based on cell and metabolic disorders, but its etiological role has not yet been determined. ⑤ Others include virus theory, immune disorder theory and brain injury theory.  2) Risk factors: The most important risk factors for Alzheimer's disease are age, gender and genes. The older you are, the higher the probability of getting the disease. Women are more likely to get the disease than men. People with family genes are more likely to get the disease. |
| Neurologist 2:  Answer: The etiology of Alzheimer's disease: The etiology is still unclear. There are many theories, including: neurotoxic injury, infection, inflammatory response, oxidative stress, and immune function deficiency. Environmental factors may also be included.  Risk factors for Alzheimer's disease are: advanced age; female gender; family history of Alzheimer's disease; poor attitude; low education or IQ; history of depression or major adverse life events; history of traumatic brain injury; Unhealthy lifestyles such as staying up late, smoking, excessive drinking, not exercising, not using the brain; overnutrition, obesity or nutritional imbalance; systemic or metabolic diseases, such as cardiovascular and cerebrovascular diseases, "three highs"; environmental risk factors, etc. wait. For some Alzheimer's disease risk factors, risk reduction actions can be taken, such as: avoid excessive drinking; avoid excess weight; avoid central obesity; treat hypercholesterolemia; treat type 2 diabetes; treat high blood pressure; avoid contact killing Insecticides; avoid head trauma; reduce the intake of saturated fatty acids and meat; quit smoking; correct bad mentality, prevent depression, etc. |
| Neurologist 3:  Etiology: 1. Genetics 2. Senile plaques 3. Neurofibrillary tangles 4. Oxidative stress 5. Neurotransmitters  Risk factors: Age is significantly associated with the prevalence of Alzheimer's disease. The older you are, the higher the prevalence rate is. For the elderly over 60 years old, the prevalence rate doubles for every five years, and female patients are about twice that of male patients.  It is relatively certain that Alzheimer's disease is related to genetics. Most epidemiological studies suggest that family history of dementia is a risk factor for Alzheimer's disease. The allele of apolipoprotein e, ε4 is Alzheimer's disease. An important risk factor for Alzheimer's disease, the frequency of the apolipoprotein e, ε4 allele is about 40% in autopsy-proven Alzheimer's disease patients, and about 16% in normal controls.  Traumatic brain injury as a risk factor for Alzheimer's disease has been reported a lot. Severe brain trauma may be one of the causes of Alzheimer's disease. The relative risk of Alzheimer's disease when there is hypothyroidism high.  Depression, especially first-episode depression in old age, is a risk factor for Alzheimer's disease. The level of education is related to the increased prevalence of Alzheimer's disease. The possible explanation is that early education training promotes the development of cortical synapses, which increases the number of synapses and increases brain reserve, thereby reducing the risk of dementia. |
| Neurologist 4:  The common etiology and risk factors of Alzheimer's disease include genetic factors, vascular factors, emotional factors and many other factors. Genetic factors can cause the disease, but it is not very common; vascular factors are more common, such as diabetes and hyperlipidemia, high blood pressure, etc. can induce Alzheimer's disease; chronic damage of various poisons, long-term depression and other conditions may also cause this disease. |

| Question 3: If a parent had/has Alzheimer's disease, are their children at greater risk of getting it? Is Alzheimer's disease hereditary? |
| --- |
| GPT3 Chinese version:  A person's risk of developing the disease increases slightly if one of their parents has Alzheimer's disease. Specifically, studies have shown that if a person has one parent with Alzheimer's disease, that person's risk of developing the disease is roughly double that of the general population. If both parents have Alzheimer's disease, that person's risk is higher.  Alzheimer's disease may involve a variety of genetic and environmental factors. Although certain genetic mutations are associated with Alzheimer's disease, Alzheimer's disease is not a clearly inherited disease, and no single gene can explain the full spectrum of disease manifestations. In most cases, the onset of Alzheimer's disease is caused by the interaction of multiple genes and environmental factors.  So, while having Alzheimer's in the family may increase a person's risk, it doesn't necessarily have to be passed on to the next generation. However, since Alzheimer's disease is a very common form of dementia, preventive measures and early screening are important to reduce risk and diagnose early. |
| GPT3 English version:  Yes, having a parent with Alzheimer's disease does increase the risk of developing the disease. Alzheimer's disease is influenced by a complex interplay of genetic, environmental, and lifestyle factors. While genetics do play a role, it is important to note that having a family member with Alzheimer's disease does not guarantee that a person will develop the disease.  There are two types of Alzheimer's disease: early-onset and late-onset. Early-onset Alzheimer's disease is rare, accounting for less than 5% of all cases, and is usually caused by a genetic mutation. In contrast, late-onset Alzheimer's disease, which accounts for the vast majority of cases, is thought to be caused by a combination of genetic and environmental factors.  Studies have identified several genes that are associated with an increased risk of developing late-onset Alzheimer's disease. The most well-known of these genes is the apolipoprotein E (APOE) gene. There are three common variants of the APOE gene: APOE2, APOE3, and APOE4. People who inherit one copy of the APOE4 variant have an increased risk of developing Alzheimer's disease, while those who inherit two copies of the variant have an even greater risk. However, it's important to note that not all people with the APOE4 variant will develop Alzheimer's disease, and not all people who develop Alzheimer's disease have the APOE4 variant.  In summary, while having a parent with Alzheimer's disease does increase the risk of developing the disease, it's not a guarantee. Genetic testing can provide some information about a person's risk, but it's important to remember that Alzheimer's disease is influenced by a combination of genetic and environmental factors, and there is still much to learn about the disease. |
| GPT4 Chinese version:  Yes, if one of the parents has Alzheimer's disease, the risk of the child is relatively increased. Alzheimer's disease is hereditary, but not absolute. Alzheimer's disease has been linked to gene variants that may lead to the accumulation of abnormal proteins in the brain that increase the risk of the disease, research has found.  It is worth noting that genetic factors are not the only factors that affect the risk of Alzheimer's disease. Factors such as environment, lifestyle and age also have an impact on the risk of developing Alzheimer's disease. Therefore, even if there is a family history, it does not mean that the child will definitely develop Alzheimer's disease. Maintaining a healthy lifestyle, such as eating right, exercising moderately, and staying socially active, can reduce the risk of the disease. If in doubt, it is recommended to consult a professional doctor for evaluation and advice. |
| GPT4 English version:  Alzheimer's disease (AD) is a complex neurological disorder, and its causes are not completely understood. However, research has identified genetic and environmental factors that influence the risk of developing the disease. Both hereditary and non-hereditary factors can contribute to a person's risk of developing Alzheimer's.  There are two types of Alzheimer's disease: early-onset (less common) and late-onset (more common). Early-onset AD typically occurs before the age of 65 and is more strongly linked to genetic factors. Several genes have been identified that can cause early-onset AD, including mutations in the APP, PSEN1, and PSEN2 genes. If a parent has a mutation in one of these genes, their children have a 50% chance of inheriting the mutation and are at a higher risk of developing early-onset AD.  Late-onset AD is more common and usually occurs after the age of 65. The genetic factors influencing late-onset AD are less well understood, but one known genetic risk factor is the presence of the APOE ε4 allele. Having one or two copies of the APOE ε4 allele increases a person's risk of developing Alzheimer's. However, not everyone with the APOE ε4 allele will develop AD, and some people without the allele can still develop the disease. This suggests that a combination of genetic, environmental, and lifestyle factors contribute to the risk of late-onset AD.  In summary, if a parent had or has Alzheimer's disease, their children may be at a greater risk of developing the disease, depending on the specific genetic factors involved. Alzheimer's disease can have a hereditary component, but it is not solely determined by genetics. Other factors such as age, environment, and lifestyle can also influence an individual's risk. |
| Neurologist 1:  According to official statistics, in fact, only 5% of the Alzheimer's disease population is monogenic. The proportion of disease caused by it is still relatively low. |
| Neurologist 2:  Answer: The vast majority of Alzheimer's disease is sporadic, and a small part is hereditary. If there are two or more immediate family members with Alzheimer's disease, then it is necessary to actively look for genetic factors. For example, carrying some risk genes for Alzheimer's disease, such as APOE, increases the risk of Alzheimer's disease by 2-3 times. |
| Neurologist 3:  Alzheimer's disease is inherited, and mutations in three autosomal dominant genes can run in families, so children are also at increased risk of the disease. |
| Neurologist 4:  (1) Alzheimer's disease is divided into familial and sporadic. Familial AD is an autosomal dominant inheritance, that is, one of the parents has Alzheimer's disease. If there is no gene mutation, the child, regardless of gender, will have the gene that induces AD and cause the disease. (2) The amyloid precursor protein gene located on chromosome 21, the presenilin 1 gene located on chromosome 14, and the presenilin 2 gene mutation located on chromosome 1 are common genetic genes in familial AD. Carriers of the first two genes are almost 100% of the disease, and 95% of the latter are also diseased.  (3) Sporadic is the condition of the disease without inheritance. Generally speaking, we believe that the mutation of the apolipoprotein E gene is most closely related to this condition. |

| Question 4: What is the role of regular physical examination and health assessments help the prevention of Alzheimer's disease? |
| --- |
| GPT3 Chinese version:  Regular physical exams and health evaluations can help to identify and manage risk factors and early symptoms that may lead to Alzheimer's disease, which can help prevent or delay the progression of the disease. Here are some risk factors and early symptoms that may be associated with Alzheimer's disease:  High blood pressure: High blood pressure may lead to cerebrovascular disease, which may increase the risk of Alzheimer's disease. By measuring your blood pressure regularly and treating it promptly, you can reduce the effects of high blood pressure on your brain.  High cholesterol: High cholesterol may increase the risk of Alzheimer's disease. Regularly checking your cholesterol levels and taking the necessary steps to manage them can reduce this risk.  Diabetes: Diabetes may lead to neurological damage and decline in cognitive function, which increases the risk of Alzheimer's disease. Regularly checking blood sugar levels and maintaining a healthy diet and lifestyle can reduce the risk of diabetes.  Mild cognitive impairment: Mild cognitive impairment is one of the early symptoms of Alzheimer's disease. Regular assessment of cognitive function can help in early detection and necessary measures can be taken.  Regular physical exams and health evaluations can help identify and manage risk factors and early symptoms that may lead to Alzheimer's disease, which can help prevent or delay the progression of the disease. |
| GPT3 English version:  Regular physical examinations and health assessments are important in the prevention of Alzheimer's disease because they can help identify risk factors and early signs of cognitive decline, which can be addressed and managed before they develop into full-blown Alzheimer's disease.  Physical examinations can detect health problems such as high blood pressure, high cholesterol, and diabetes, which are risk factors for Alzheimer's disease. By managing these conditions, individuals can lower their risk of developing Alzheimer's disease.  Cognitive assessments, which are often part of a routine physical examination, can detect early signs of cognitive decline, such as problems with memory, language, and attention. Early detection of cognitive decline can allow for early intervention, such as lifestyle changes, medication, and cognitive training, which can slow the progression of cognitive decline and possibly prevent Alzheimer's disease.  Furthermore, regular physical activity has been shown to be beneficial for brain health and can lower the risk of developing Alzheimer's disease. Regular physical examinations can help identify areas where an individual can improve their physical activity levels and overall health.  In summary, regular physical examinations and health assessments can help identify risk factors for Alzheimer's disease, detect early signs of cognitive decline, and provide opportunities for early intervention, which can all contribute to the prevention of Alzheimer's disease. |
| GPT4 Chinese version:  Regular checkups and health assessments can help prevent Alzheimer 's disease or detect symptoms early so aggressive interventions can be taken. Although there is currently no cure for Alzheimer's disease, timely detection and management of the condition can have a positive impact on a patient's quality of life.  The benefits of regular checkups and health evaluations include:  Early detection: Cognitive decline, which may be an early sign of Alzheimer's disease, can be detected earlier through tests of cognitive function.  Risk Factor Management: Regular physical exams can help identify and manage some of the risk factors associated with the development of Alzheimer's disease, such as high blood pressure, diabetes, and high cholesterol. Controlling these factors can reduce the risk of disease.  Lifestyle Modifications: A health assessment can help you understand the impact of your lifestyle on your brain health. Lifestyle improvements, such as increasing exercise, eating a healthy diet, staying socially active, and getting healthy sleep, can reduce the risk of Alzheimer's disease.  Mental health: Regular assessments can detect mental health problems, such as depression or anxiety. Dealing with these issues can improve quality of life and possibly reduce the risk of Alzheimer's disease.  Medication: If early symptoms of Alzheimer's disease are seen, your doctor may recommend medication to slow the progression of the disease. Although these drugs cannot cure the disease, they may slow down the progression of the disease to some extent and improve the quality of life of patients.  In conclusion, regular physical examination and health assessment are helpful for early detection of Alzheimer's disease, effective prevention and intervention measures can be taken to reduce the risk of disease progression and improve the quality of life of patients. |
| GPT4 English version:  Regular physical examinations and health assessments can play a significant role in the prevention of Alzheimer's disease. Although there is no definitive way to prevent Alzheimer's, these evaluations can help in several ways:  Early detection: Regular health assessments can help detect early signs of cognitive decline, which may be indicative of Alzheimer's disease or other forms of dementia. Early diagnosis allows for prompt intervention, which may slow down the progression of the disease and improve the quality of life for the patient.  Identifying risk factors: Health assessments can identify modifiable risk factors for Alzheimer's disease, such as hypertension, high cholesterol, diabetes, obesity, and sleep disorders. By addressing these factors through lifestyle changes and medical interventions, the risk of developing Alzheimer's may be reduced.  Promoting a healthy lifestyle: Physical examinations and discussions with healthcare providers can encourage individuals to adopt a healthy lifestyle, which is associated with a lower risk of Alzheimer's. A healthy lifestyle includes regular physical activity, a balanced diet, maintaining a healthy weight, getting enough sleep, and managing stress.  Monitoring cognitive health: Regular cognitive assessments can help track any changes in memory and thinking skills, enabling healthcare providers to intervene if necessary.  Mental health assessment: Mental health issues, such as depression and anxiety, can increase the risk of developing Alzheimer's disease. Regular health assessments can help identify and treat these conditions, which may reduce the risk of Alzheimer's.  Medication management: Some medications can increase the risk of developing Alzheimer's or worsen cognitive symptoms. Regular health assessments allow healthcare providers to review medications and make necessary adjustments.  While there is no guarantee that regular physical examinations and health assessments will prevent Alzheimer's disease, they can contribute to overall health and well-being, which may reduce the risk of developing the disease or slow its progression. |
| Neurologist 1:  Regular physical examination and health assessment can understand the current physical condition, detect patients early, treat them in time, and delay the course of the disease. |
| Neurologist 2:  Regular physical examination and health assessment are of positive significance to the prevention of Alzheimer's disease. Mainly reflected in the management and reduction of risk factors for Alzheimer's disease, such as: overnutrition, obesity or nutritional imbalance; systemic or metabolic diseases, such as cardiovascular and cerebrovascular diseases, "three highs"; treatment of hypercholesterolemia; Treatment of type 2 diabetes; treatment of high blood pressure; correction of bad attitude, prevention of depression, etc. |
| Neurologist 3:  1) Neuropsychological Test  Mini-Mental Examination (MMSE): The content is concise, the measurement time is short, and it is easily accepted by the elderly. It is currently the most common scale to measure the degree of intellectual impairment in this disease. The total score of the scale is related to the level of education. If the illiteracy is ≤17 points; the primary school level is ≤20 points; the middle school level is ≤22 points; and the university level is ≤23 points, it means that there is cognitive impairment. Further detailed neuropsychological tests should be performed to assess cognitive functions such as memory, executive function, language, use, and visuospatial abilities. For example, the Alzheimer's Disease Assessment Scale Cognitive Component (ADAS-cog) is an 11-item cognitive battery designed specifically to detect changes in the severity of Alzheimer's disease, but is primarily used in clinical trials.  Assessment of activities of daily living: For example, the assessment of activities of daily living (ADL) scale can be used to assess the degree of impairment of patients' daily life functions. The scale has two parts: one is the physical self-care ability scale, which measures the patient's ability to take care of himself (such as dressing, undressing, combing hair, brushing teeth, etc.); the other is the tool use ability scale, which measures the patient's ability to use Ability for tools of daily living (e.g. making phone calls, taking the bus, cooking for yourself, etc.). The latter are more susceptible to cognitive decline early in the disease.  Assessment of behavioral and psychiatric symptoms (BPSD): including Alzheimer's Disease Behavioral Pathology Rating Scale (BEHAVE-AD), Neuropsychiatric Symptoms Inventory (NPI) and Cohen-Mansfield Agitation Inventory (CMAI), etc. The information provided by the baseline evaluation not only detects the presence or absence of symptoms, but also evaluates the frequency, severity, and burden on caregivers. Repeated evaluations can also monitor the effect of treatment. The Cornell Depression Scale for Dementia (CSDD) focuses on evaluating agitation and depression in dementia, and the 15-item Geriatric Depression Scale can be used to evaluate depressive symptoms in Alzheimer's disease. In contrast, CSDD had higher sensitivity and specificity, but it was not related to the severity of dementia.  2) blood test  It is mainly used to discover accompanying diseases or complications, discover potential risk factors, and rule out dementia caused by other causes. Including blood routine, blood sugar, blood electrolytes including blood calcium, kidney function and liver function, vitamin B12, folic acid level, thyroxine and other indicators. Serological tests for syphilis, human immunodeficiency virus, and Borrelia burgdorferi should be performed for high-risk groups or those with clinical symptoms.  3) Neuroimaging examination  Structural Imaging: Used to rule out other underlying diseases and detect specific imaging findings in Alzheimer's disease.  CT (thin-layer scan) and MRI (coronal) examination of the head can show obvious atrophy of the cerebral cortex, especially the hippocampus and medial temporal lobe, which supports the clinical diagnosis of Alzheimer's disease. Compared with CT, MRI is useful for detecting subcortical vascular changes (e.g. critical infarcts) and suggestive of specific diseases (e.g. multiple sclerosis, progressive supranuclear palsy, multiple system atrophy, corticobasal degeneration, prion diseases, frontal Temporal lobe dementia, etc.) are more sensitive to changes.  Functional neuroimaging, such as positron emission tomography (PET) and single photon emission computed tomography (SPECT), can improve dementia diagnostic confidence.  18F-Deoxyribose Glucose Positron Emission Scanning (18FDG-PET) can show decreased glucose metabolism in the temporal parietal and superior /posterior temporal regions, posterior cingulate cortex, and precuneus, revealing specific abnormal changes in Alzheimer's disease. Reduced frontal lobe metabolism can be seen in the late stage of Alzheimer's disease. 18FDG-PET has a sensitivity of 93% and a specificity of 63% for the pathological diagnosis of Alzheimer's disease. It has become a practical tool, especially for the differential diagnosis of Alzheimer's disease and other dementias.  Amyloid PET imaging is a very promising technique, but it is not currently used routinely.  4) Electroencephalogram (EEG)  The EEG of Alzheimer's disease is characterized by decreased alpha waves, increased theta waves, and decreased average frequency. But 14% of patients had normal EEG early in the disease. EEG is used in the differential diagnosis of Alzheimer's disease, which can provide early evidence of prion disease, or suggest the possible presence of intoxication-metabolic abnormalities, transient epileptic amnesia, or other epileptic disorders.  5) Cerebrospinal fluid detection  Cerebrospinal fluid counts, protein, glucose, and protein electrophoresis: Testing should be done in patients suspected of vasculitis, infection, or demyelinating disease. Patients with rapidly progressive dementia should undergo 14-3-3 protein examination, which is helpful for the diagnosis of prion disease.  Detection of β-amyloid and Tau protein in cerebrospinal fluid: The level of β-amyloid (Aβ42) in the cerebrospinal fluid of patients with Alzheimer's disease decreases (due to the deposition of Aβ42 in the brain, the Aβ42 content in the cerebrospinal fluid decreases), and the total Tau protein or phosphorylated Elevated tau protein. Studies have shown that the diagnostic sensitivity of Aβ42 is 86%, and the specificity is 90%; the diagnostic sensitivity of total Tau protein is 81%, and the specificity is 90%; the diagnostic sensitivity of phosphorylated Tau protein is 80%, and the specificity is 92%. Compared with the control, the sensitivity of diagnosing Alzheimer's disease can reach 85% to 94%, and the specificity is 83% to 100%. These markers can be used to support the diagnosis of Alzheimer's disease, but the specificity of distinguishing Alzheimer's disease from other dementia diagnoses is low (39% to 90%). At present, there is still a lack of unified testing and sample processing methods.  6) Genetic testing  Can provide a reference for diagnosis. Amyloid precursor protein gene (APP), presenilin 1, 2 gene (PS1, PS2) mutations account for 50% of familial early-onset Alzheimer's disease. Apolipoprotein APOE4 gene detection can be used as a reference for sporadic Alzheimer's disease. |
| Neurologist 4:  Alzheimer's disease is usually preventable. Since the causes of Alzheimer's disease are closely related to cerebrovascular diseases, it is necessary to actively cooperate with doctors to control the factors that induce the disease, and go to the hospital for regular health checkups. If there is any abnormality, it is necessary to actively cooperate with the doctor to control the condition. Maintaining adequate sleep time, a healthy diet and a good attitude, insisting on doing some exercise, and appropriately increasing some mental activities can help prevent Alzheimer's disease. |

| Question 5: How to prevent and manage memory impairment in patients with Alzheimer's disease? |
| --- |
| GPT3 Chinese version:  Alzheimer 's disease is a common neurodegenerative disorder that often leads to memory impairment and cognitive decline. While there is currently no cure for Alzheimer's disease, there are ways to prevent and manage memory impairment that can help patients relieve symptoms and improve their quality of life. Here are some suggestions:  Eat a healthy diet: Eating a healthy diet is critical to preventing and managing Alzheimer's disease. It is recommended that patients eat more fruits, vegetables, whole grain bread, beans, fish and nuts, and avoid foods high in fat, cholesterol and sugar. In addition, patients may consider supplementing with beneficial nutrients such as omega-3 fatty acids, folic acid, vitamin B6, and vitamin B12.  Physical exercise: Moderate physical activity can improve blood circulation and improve cardiorespiratory fitness, which is very beneficial for the prevention and management of Alzheimer's disease. It is recommended that patients do moderate amount of aerobic exercise, such as brisk walking, running, swimming, cycling, etc., at least 150 minutes of exercise per week.  Challenge your brain: Activities such as reading books, completing math problems, and solving difficult problems can help patients exercise their brains and enhance memory and cognitive skills. In addition, learning new skills, learning new languages, participating in social activities, etc. can also improve the brain vitality of patients.  Get enough sleep: Getting enough sleep can help the brain recover and repair, which can help prevent and manage Alzheimer's disease. It is recommended that patients maintain a sleep time of 7-9 hours per night and establish regular sleep habits.  Manage stress: Chronic stress may lead to decreased brain function and increase the risk of Alzheimer's disease. Patients can adopt some methods of coping with stress, such as exercising, meditating, reading, listening to music, etc.  Regular medical checkups: Patients need regular medical checkups to detect and treat potential health problems early.  In conclusion, the key to preventing and managing Alzheimer's disease is adopting a series of healthy lifestyle and habits, including healthy eating, moderate physical activity, challenging the brain, getting enough sleep, managing stress and getting regular medical check-ups. If you or a loved one suffers from Alzheimer's disease or other forms of memory impairment, you should consult a professional physician and healthcare provider in order to develop an appropriate treatment and management plan. |
| GPT3 English version:  Alzheimer's disease is a progressive neurodegenerative disorder that affects memory and cognitive function. While there is no cure for Alzheimer's disease, there are several strategies that can be employed to prevent or manage memory impairment in patients with this condition.  Encourage Physical Exercise: Physical exercise has been shown to improve cognitive function and reduce the risk of developing Alzheimer's disease. It is important to encourage patients to engage in regular physical activity, such as walking, swimming, or cycling.  Healthy Diet: A healthy diet can also help to reduce the risk of developing Alzheimer's disease. Patients should be encouraged to consume a diet rich in fruits, vegetables, whole grains, lean protein, and healthy fats such as those found in fish and nuts.  Cognitive Stimulation: Cognitive stimulation exercises can help to improve memory and cognitive function in patients with Alzheimer's disease. These exercises can include puzzles, reading, and playing games that challenge the mind.  Medications: Medications such as cholinesterase inhibitors and memantine can help to manage the symptoms of Alzheimer's disease and slow down the progression of the disease.  Lifestyle changes: Patients should be advised to quit smoking, limit alcohol consumption, and manage stress levels as these factors have been shown to worsen cognitive function.  Social support: Social support from family, friends, and support groups can help to improve the patient's emotional well-being and reduce the risk of depression.  Routine: Patients with Alzheimer's disease benefit from routine and structure in their daily lives. Having a daily routine can help to reduce confusion and anxiety.  It is important to note that while these strategies can help to prevent or manage memory impairment in patients with Alzheimer's disease, there is currently no cure for this condition. Therefore, a multidisciplinary approach involving healthcare professionals, caregivers, and family members is essential to provide comprehensive care for patients with Alzheimer's disease. |
| GPT4 Chinese version:  Alzheimer's disease is a common neurodegenerative disease that causes symptoms such as memory impairment and cognitive decline in patients. Currently, there is no cure for Alzheimer's disease, but there are steps you can take to prevent and manage memory impairment in patients:  Maintain a healthy lifestyle: Eating a balanced diet, exercising moderately, getting a good night's sleep, and reducing stress all play a positive role in preventing and managing Alzheimer's disease.  Stay socially active: Keeping in touch with family and friends and participating in social activities can help patients maintain mental health and slow down the deterioration of symptoms.  Cognitive training: Performing various cognitive activities, such as reading, writing, puzzles, Sudoku, etc., can help delay the decline of cognitive ability.  Drug treatment: Using drugs for Alzheimer's disease under the guidance of a doctor, such as Mecrolol, Donepezil, etc., can relieve symptoms.  Environmental adjustment: create a safe and comfortable living environment for patients, reduce mood swings and stress. For example, use labels to remind patients of the location of daily necessities, keep the home environment tidy, and avoid frequently changing the location of items.  Rely on memory aids: Use notebooks, cell phone reminders, calendars, and other tools to help patients remember important things.  Support from family members and caregivers: Loved ones and caregivers are patient, caring, and supportive, understanding the needs and difficulties of the patient, and assisting them with their daily lives.  Periodic examination: regular neuropsychological assessment, in order to detect changes in patients' symptoms in time, and adjust treatment and nursing plans.  Note that these methods do not cure Alzheimer's disease, but may help patients cope better with memory impairment and improve their quality of life. Follow your professional doctor's advice and see your doctor regularly. |
| GPT4 English version:  I am not a medical professional, but I can offer some general information on preventing and managing memory impairment in patients with Alzheimer's disease. It's important to consult with a healthcare professional for personalized advice and guidance.  Early diagnosis: Early detection of Alzheimer's disease can help identify appropriate interventions and support systems.  Medications: Some medications may help slow the progression of Alzheimer's or manage symptoms. Commonly prescribed drugs include cholinesterase inhibitors (e.g., donepezil, rivastigmine, and galantamine) and memantine.  Cognitive stimulation: Engaging in cognitively stimulating activities, such as puzzles, reading, or learning new skills, can help maintain mental function.  Physical exercise: Regular physical activity has been linked to better brain health and may help slow the progression of Alzheimer's.  Healthy diet: A diet rich in fruits, vegetables, whole grains, lean proteins, and healthy fats, such as the Mediterranean or DASH diet, can support brain health.  Social engagement: Staying socially active and maintaining strong relationships can have a positive impact on brain health.  Quality sleep: Prioritizing good sleep hygiene can help maintain cognitive function.  Stress management: Engaging in stress-reduction techniques, such as meditation, deep breathing, or yoga, can support overall brain health.  Regular medical care: Routine check-ups and working closely with healthcare professionals can help manage Alzheimer's disease and identify other health issues that may impact memory.  Assistive devices and strategies: Using memory aids, such as calendars, reminders, and labeling items, can help patients maintain independence and manage memory impairment.  Remember, it's crucial to consult a healthcare professional for personalized advice and to establish a comprehensive care plan for Alzheimer's patients. |
| Neurologist 1:  Train the patient's instantaneous memory ability, that is, tell the patient some content and let the patient retell it immediately until he can't retell it. Train the patient's long-term memory ability, that is, let the patient recall a certain content in the past. If the patient can successfully recall, then recall the content of a longer time ago, increasing the training difficulty; if the patient cannot successfully recall, then recall the relatively recent content, reducing the training difficulty, repeatedly train until you can memorize. It can also sort out the ins and outs of interpersonal relationships, express the relationship and characteristics of various things, strengthen the understanding and memory of people and things, and enhance memory. For patients who really have memory difficulties, it is recommended that patients record some important things in a fixed notebook to help memory and avoid forgetting. |
| Neurologist 2:  For memory damage, in addition to taking nootropic drugs under the guidance of a doctor, appropriate memory training can also be carried out to maintain the patient's original memory as much as possible and delay the further decline of memory. Training memory can prevent the aging of the brain, and it is a good prescription for strengthening the brain. Memory not only has a great effect on people's work, study, and life, but also an important means to ensure human health and delay brain aging. |
| Neurologist 3:  First, to prevent brain damage, we must prevent and treat any related diseases that are prone to brain damage. Second, we need a healthy diet, eat more foods rich in vitamin B, vitamin C and choline. Third, in general, healthy lifestyles such as reasonable diet, moderate exercise, good learning, positive emotions, and active prevention and treatment of related diseases are beneficial to the prevention The occurrence of memory impairment is somewhat helpful. |
| Neurologist 4:  The prevention and management of memory impairment in patients with Alzheimer's disease can be carried out from the following aspects: (1) In terms of diet, eat more foods rich in B vitamins and folic acid, and eat more walnuts, which have obvious brain nourishing effects , can improve memory ; (2) In terms of exercise, regular aerobic exercise, such as jogging, yoga, Tai Chi, etc., can be maintained for more than 30 minutes each time, so as not to feel tired ; (3) Use your brain more, you can do mental work in your daily life, such as painting, listening to music, playing go, or going to a university for the elderly to learn new knowledge; (4) Communicate more with the outside world and avoid living alone ; (5) Avoid some bad things Lifestyle habits, such as quitting smoking and drinking. |

| Question 6: What are the treatments options for Alzheimer's disease? Please provide me alternative suggestions from the aspects of pharmaceutical, psychosocial, and caregiving. |
| --- |
| GPT3 Chinese version:  Alzheimer's disease is a progressive neurodegenerative disease for which there is currently no cure. However, several drug and non-drug treatments can help patients relieve symptoms and improve quality of life. At the same time, psychosocial support and care for people with Alzheimer's disease is also very important.  In terms of drug treatment:  1. Acetylcholinesterase inhibitors: These drugs increase levels of acetylcholine in the brain, which improves cognition and memory.  2. Glutamine receptor antagonists: These drugs reduce the release of glutamate, thereby reducing symptoms.  3. Antidepressants: This drug can improve emotional states and behavioral problems in people with Alzheimer's disease.  Antipsychotic medication: This medication can help control hallucinations and delusions in people with Alzheimer's disease.  Psychosocial support:  1. Provide emotional support: People with Alzheimer's disease often face challenges and difficulties, so providing emotional support can help them reduce stress and anxiety.  2. Establish social networks: Helping patients establish and maintain social networks can help them maintain social and cognitive function.  3. Provide cognitive stimulation: Providing cognitive stimulation and social activities can help patients maintain cognitive ability and promote brain function.  Nursing:  1. Regular work and rest: Providing regular work and rest and regular medication can help Alzheimer's patients maintain their physical health and living habits.  2. Safety guarantee: prevent patients from getting lost or being injured, and provide safety guarantee measures.  3. Daily care: providing basic daily care, such as eating, bathing, dressing, etc., can help patients stay healthy.  People with Alzheimer's disease need comprehensive treatment and attention. Medication can help relieve symptoms, psychosocial support can provide emotional and cognitive support, and care can help patients maintain physical health and quality of life. However, treating Alzheimer's disease is not a simple task and requires long-term efforts and attention from medical staff and family members. Here are some additional suggestions:  1. Family members need training: To better care for the patient, family members need training on how to deal with the patient's behavioral and emotional problems and how to provide day-to-day care for the patient.  2. Regular follow-up visits: patients need regular follow-up visits to monitor the progress of the disease and adjust the treatment plan.  3. Maintain a healthy lifestyle: Adopting a healthy lifestyle as much as possible, such as a healthy diet, regular exercise, and adequate sleep, can help patients maintain good health.  4. Provide a safe and comfortable living environment: Providing patients with a safe and comfortable living environment can help them reduce anxiety and tension.  Treating Alzheimer's disease requires a comprehensive approach that involves medication, psychosocial support, and nursing. Family members and medical staff need to work closely together to provide long-term attention and care to patients to improve their quality of life and alleviate their suffering. |
| GPT3 English version:  Alzheimer's disease is a progressive neurodegenerative disorder that affects the brain, leading to cognitive decline and memory loss. While there is no cure for Alzheimer's disease, there are several treatment options available that can help manage the symptoms and improve the quality of life for individuals with the disease. Here are some alternative suggestions from the aspects of pharmaceutical, psychosocial, and caregiving:  Pharmaceutical treatments:  Cholinesterase inhibitors: These medications help to increase the levels of acetylcholine, a neurotransmitter that is important for memory and learning. Examples include donepezil (Aricept), rivastigmine (Exelon), and galantamine (Razadyne).  Memantine: This medication works by regulating the activity of glutamate, a neurotransmitter that is involved in learning and memory. Memantine is often prescribed in combination with a cholinesterase inhibitor.  Antidepressants: These medications may be prescribed to help manage depression and anxiety, which are common in individuals with Alzheimer's disease.  Psychosocial treatments:  Cognitive stimulation therapy: This involves structured activities and exercises that are designed to stimulate cognitive function and improve memory.  Reality orientation therapy: This involves providing cues and reminders about the time, place, and situation to help individuals with Alzheimer's disease maintain their sense of reality.  Music therapy: This involves listening to music or playing musical instruments, which has been shown to help reduce anxiety and improve mood in individuals with Alzheimer's disease.  Caregiving treatments:  Respite care: This involves taking a break from caregiving responsibilities and having someone else care for the individual with Alzheimer's disease for a short period of time.  Home modifications: Making modifications to the home environment, such as installing handrails and removing trip hazards, can help reduce the risk of falls and other accidents.  Support groups: Joining a support group can provide emotional support and practical advice for caregivers of individuals with Alzheimer's disease.  It's important to note that the best treatment plan for Alzheimer's disease will depend on the individual's specific symptoms and needs. Consulting with a healthcare professional or a specialist in geriatric care can help you determine the best course of action for your loved one. |
| GPT4 Chinese version:  Alzheimer's disease is a common neurodegenerative disease, mainly manifested as cognitive dysfunction and behavioral abnormalities. There is currently no cure, but there are a variety of treatments that can relieve symptoms and slow the course of the disease. The following are medical, psychosocial and nursing recommendations for treatment:  medical treatement:  Drug therapy is mainly used to relieve symptoms and delay the course of the disease. Major drugs include:  Acetylcholinesterase inhibitors (such as donepezil, ribavirin, Calgol, etc.): These drugs improve nerve conduction by increasing the concentration of acetylcholine in the brain, and have a certain effect on patients with mild to moderate Alzheimer's disease.  NMDA receptor antagonists (such as memantine): These drugs have some effect on patients with moderate to severe Alzheimer's disease by regulating the calcium ion homeostasis of neurons.  Other medications: Antidepressants, anxiolytics, antipsychotics, etc. can be used to treat the emotional and behavioral problems associated with Alzheimer's disease.  Psychosocial support: Psychosocial support is an important part of the care of people with Alzheimer's disease and can improve the quality of life for people with Alzheimer's disease. include:  Cognitive rehabilitation training: such as memory training, language training, social skills training, etc., to improve the cognitive function of patients.  Psychotherapy: Such as psychological counseling, cognitive behavioral therapy, etc., to help patients cope with psychological and behavioral problems.  Social activities: Encourage patients to participate in various social activities, such as family gatherings, community activities, etc., to enhance interpersonal communication and emotional support.  Nursing Measures: Provide individualized care measures for the special needs of Alzheimer's patients:  Environmental adjustment: keep the home environment safe, tidy, spacious and bright, and prevent patients from falling or getting lost.  Daily life care: help patients to complete basic living needs such as washing, dressing, eating, and excretion, and pay attention to maintaining patients' personal hygiene.  Safety monitoring: such as installing guardrails, safety doors and other facilities to ensure that patients will not leave their homes or get lost. Use GPS positioning devices or medical ID bracelets, etc., to quickly find patients in emergencies.  Regular work and rest: assist patients to establish a regular work and rest time, ensure adequate sleep, and help relieve patients' mood swings and behavioral problems.  Nutritious diet: Provide a balanced and nutritious diet, especially foods rich in antioxidants, B vitamins, omega-3 fatty acids, etc. that are good for the brain.  Maintain communication: Maintain patient, respectful communication with patients, encouraging them to express their feelings and needs. Provide timely attention and assistance to patients' distress and needs.  Moderate exercise: Encourage patients to do moderate exercise, such as walking, Tai Chi, yoga, etc., to enhance physical strength, improve mental state, and improve quality of life.  Family support and education: Provide family members with education and support about Alzheimer's disease, helping them understand the symptoms, development and coping methods of the disease so that they can better care for the patient.  In summary, comprehensive drug therapy, psychosocial support, and nursing measures can help patients with Alzheimer's disease relieve symptoms, delay the course of the disease, and improve the quality of life. It is important to follow your professional doctor's advice to develop an individualized treatment and care plan. |
| GPT4 English version:  While there is no cure for Alzheimer's disease, various treatment options can help manage its symptoms and improve the quality of life for patients and their caregivers. These treatments can be broadly classified into three categories: pharmaceutical, psychosocial, and caregiving.  Pharmaceutical treatments:  a. Cholinesterase inhibitors: These medications, such as donepezil, rivastigmine, and galantamine, help improve communication between brain cells by preventing the breakdown of the neurotransmitter acetylcholine. They can help manage memory, thinking, and language problems in mild to moderate Alzheimer's.  b. Memantine (Namenda): This drug regulates the activity of the neurotransmitter glutamate, which plays a role in learning and memory. Memantine is often used in moderate to severe Alzheimer's to help delay the progression of symptoms.  Psychosocial interventions:  a. Cognitive stimulation therapy: This therapy involves engaging in activities and exercises that stimulate thinking, memory, and communication. It can help improve cognitive function and quality of life for people with Alzheimer's.  b. Reminiscence therapy: This intervention involves discussing past experiences, often with the help of photos, music, or familiar items, to help individuals with Alzheimer's recall and share memories.  c. Reality orientation: This approach aims to improve patients' awareness of their surroundings and the current time to reduce confusion.  d. Behavioral therapy: This type of therapy helps manage challenging behaviors, such as agitation or aggression, by identifying triggers and teaching patients and caregivers strategies to cope.  Caregiving strategies:  a. Creating a structured daily routine: A consistent daily schedule can help reduce confusion and anxiety in Alzheimer's patients.  b. Maintaining a safe and supportive environment: Removing clutter, ensuring good lighting, and using clear labels can help reduce disorientation and the risk of falls.  c. Communication strategies: Speaking slowly and clearly, using short sentences, and maintaining eye contact can help improve communication with Alzheimer's patients.  d. Encouraging physical activity: Regular exercise, such as walking or gentle stretching, can improve mood and overall well-being.  e. Providing emotional support: Providing reassurance, validation, and comfort can help alleviate stress and anxiety in Alzheimer's patients.  It's important to note that the effectiveness of these treatments and strategies may vary depending on the individual and the stage of Alzheimer's. A healthcare team, including physicians, nurses, and therapists, can help tailor a comprehensive care plan to meet the specific needs of each patient. |
| Neurologist 1:  1) Drugs: There are currently 5 drugs that can treat dementia symptoms, 3 of which are cholinesterase inhibitors (Aricept (donepezil hydrochloride), rivastigmine/rivastigmine (Exelon), Galantamine), one NMDA receptor antagonist (memantine), and one combination preparation (memantine-donepezil combination preparation).  2) Social psychology: Many epidemiological studies have proved that more participation in social activities can reduce the risk of dementia, and the depth of communication is greater than the scope of social interaction, so it is still recommended Disabled elderly people actively participate in social activities.  3) Care:  ① Pay attention to respecting the patient, understand some mental symptoms and personality changes of the patient, listen patiently to the patient's complaint, and establish a trusting relationship with the patient;  ② Encourage patients to increase their confidence in overcoming the disease, closely observe the patient's emotional and psychological dynamics, pay attention to mastering appropriate communication methods and skills, eliminate patients' ideological concerns, and achieve ideal communication effects.  ③ Make adjustments according to the individual characteristics of patients.  For patients who often suffer from anxiety, it is necessary to ensure that the room is quiet and arrange interesting activities; patients can also be instructed to listen to some relaxing and soothing music. For patients with depression, listen patiently to the patient's narration, and do not force the patient to do things he does not want. Try to meet their reasonable requirements, and avoid using words and behaviors that hurt feelings or damage the patient's self-esteem, causing them to suffer psychological harm, depression, and even aggressive behavior. |
| Neurologist 2:  For memory training for Alzheimer's patients, we should pay attention to the training process, not the training results. That is to say, it is not necessary for the patient to memorize many things, but for the patient to participate in the training and use his brain. In the process of memory training, the difficulty of memory should not be too high, and arrangements should be made according to the actual situation of the patient. If the difficulty is too high, on the one hand, the patient will not be able to complete it; on the other hand, it will increase the mental burden of the patient and cause adverse emotional reactions; It can even lead to mental and behavioral disturbances.  1. Alzheimer's patients whose memory damage is obvious but not very serious: if the patient usually likes to read newspapers, books, and watch TV, as long as the patient is still willing to watch, they should not be restricted or prevented, but should be encouraged. He reads, although the patient may not be able to read or remember seeing it at all. If the patient likes to play cards or mahjong, when the patient has not completely lost the interest and ability in this area, he can find a few people to play with him, but don't care whether the patient plays well or not. Of course, as long as the patient is willing, he can also play some simple intellectual games with him, such as playing flying chess, counting 24 points, and doing uncomplicated jigsaw puzzles.  2. For patients with severe memory impairment: put some simple and eye-catching items that are useful in daily life in their rooms, such as calendars, clocks, various toys, etc., teach them what day it is every day, and establish Have a regular life time, tell them when to get up, go to bed, eat, take medicine, take a bath, etc., so that long-term targeted training can be carried out to strengthen memory.  What are the treatments for Alzheimer's disease? Please advise on medication, psychosocial and nursing.  Answer: The treatment principles of Alzheimer's disease include: early diagnosis, timely treatment, and lifelong management. For the mental and behavioral symptoms associated with "dementia", non-drug intervention is the first choice, anti-"dementia" treatment is basic, and psychotropic drugs can be used when necessary, but the efficacy and side effects should be evaluated regularly, and long-term use should be avoided. Health education, psychological support and practical help for caregivers can improve the quality of life of Alzheimer's patients.  Drugs to improve cognition:  1. Cholinesterase inhibitors (1) Donepezil: through competitive and non-competitive inhibition of acetylcholinesterase, thereby increasing the concentration of acetylcholine in the synaptic cleft of neurons. A single daily dose can be administered. Common side effects include diarrhea, nausea, sleep disturbance, and more serious side effects are bradycardia. The recommended starting dose of donepezil is 5 mg/d. For those who are more sensitive to the drug, the initial dose can be 2·5 mg/d, which can be increased to 5 mg/d after 1 week, and the dose can be increased to 10 mg/d after 1 month. If it can be tolerated, use a dose of 10 mg/d as much as possible, and regularly review the electrocardiogram during use. (2) Rivastigmine: It is a carbamic acid that can simultaneously inhibit acetylcholinesterase and butyrylcholinesterase. When the daily dose is greater than 6 mg, its clinical efficacy is relatively certain, but when the high dose is used, the adverse reactions also increase accordingly. At present, the transdermal patch of rivastigmine is already on the market, making the drug more convenient to use.  2. Glutamate receptor antagonist: Memantine acts on the glutamate- glutamine system in the brain, and is a non-competitive N-methyl-D-aspartate antagonist with moderate affinity. The usage is an initial dose of 5 mg, increased to 10 mg in the 2nd week, 15 mg in the 3rd week, and 20 mg in the 4th week, once a day, orally. For patients with impaired renal function, the dose of memantine should be reduced accordingly. For patients with moderate or severe Alzheimer's disease, the combination therapy of a cholinesterase inhibitor and memantine can achieve better cognition, daily living ability and social function, and improve mental and behavioral symptoms.  Drug therapy for psychobehavioral symptoms  1. Antipsychotic drugs: mainly used to control severe hallucinations, delusions and symptoms of excitement and impulse. The use of antipsychotic drugs should follow the principle of "starting with a small dose, slowly increasing according to the treatment response and adverse reactions, and slowly reducing the dose until the drug is stopped after the symptoms are controlled". Commonly used drugs include risperidone, olanzapine, and quetiapine. ➤Initial dose of risperidone 0·25-0·50 mg/d, maximum dose 2 mg/d, divided into 1-2 doses; ➤Olanzapine 1·25-2·50 mg/d, maximum dose 10 mg/d, divided into 1-2 doses 2 doses; ➤Quetiapine 12·5 mg/d, the maximum dose is 200 mg/d, divided into 1-3 doses. ➤For the elderly (usually over 85 years old), 1/2 of the recommended dose can be selected as the starting dose.  2. Antidepressants are mainly used to treat depression, mild agitation and anxiety. Commonly used drugs such as trazodone (25-100 mg), sertraline (25-100 mg), citalopram (10-20 mg, pay attention to QTc interval), mirtazapine (7·5-30 mg), etc.  3. Mood stabilizers can relieve symptoms such as impulsivity and agitation. Commonly used drugs such as sodium valproate (250-1000 mg).  Alzheimer's disease in psychosocial aspects: promote the patient to continue social and physical activities, increase intellectual stimulation, reduce cognitive problems, deal with behavioral problems, resolve family conflicts and improve social support.  For Alzheimer's disease, nursing is also a kind of treatment, and to a certain extent, nursing is more important than drugs. |
| Neurologist 3:  Drugs: 1. Anti-dementia drugs: donepezil, carbastin, memantine.  Antipsychotic drugs: risperidone, olanzapine, quetiapine, etc., start with a small dose, and use according to the principle of slowly increasing the therapeutic response and adverse reactions.  Antidepressants: Sertraline, Citalopra, etc.  Mood stabilizer: sodium valproate.  Psychosocial: The goal is to maintain the patient's cognitive and social functioning as much as possible while ensuring the patient's safety and comfort.  Help the family of the patient decide whether the patient should be hospitalized, family therapy or day care, etc.; help the family take appropriate measures to prevent the patient from committing suicide, impulsive attack and wandering, etc., so as to ensure the safety of the patient. Assisting family members with legal issues related to menopause such as testamentary capacity and other capacity issues.  Nursing: Basic care is particularly important. Caregivers need to be accompanied 24 hours a day and pay attention to the safety of care.  Cognitive and life skills training;  Home care environment: It is recommended to keep the room tidy, properly manage household appliances, etc. Ensure safety and reduce injuries. Reduce the risk of going out alone. |
| Neurologist 4:  (1) Drug treatment : (1) Treatment of cognitive symptoms: cholinesterase inhibitors: It is effective for mild to moderate AD dementia in terms of cognition, function, and overall, and it can still benefit from severe AD dementia; Nepezil 10 mg/d can produce the best maintenance effect, with outstanding cognitive benefits and good safety; carbaladin 9.5 mg/d patch can produce the best maintenance effect, with cognitive and overall benefits comparable to 12 mg/d capsules Equivalent, the safety is better than capsules; galantamine 24mg/d can produce the best maintenance effect, the overall benefit is obvious, and the safety is good; when a ChEI initial drug lacks satisfactory curative effect or is not tolerated, switch to another ChEI A ChEI achieves similar effects as the original drug. Glutamate receptor antagonists: memantine 20mg/d has slight curative effect on the cognition and overall of moderate to severe AD dementia; memantine combined with cholinesterase inhibitors has synergistic effect on cognition, overall and behavior of moderate to severe AD dementia effect.  (2) Treatment of mental and behavioral symptoms: A typical antipsychotics: A typical antipsychotics can alleviate the mental and behavioral symptoms caused by AD, but they all have the risk of aggravating cognitive impairment. Olanzapine was more effective in alleviating the mental and behavioral symptoms of AD, followed by risperidone and quetiapine. Serotonin: Pimavanserin has short-term benefits on psychotic symptoms of AD dementia.  (3) Chinese medicine treatment: Preliminary clinical studies have proved that the combination of conventional western medicine in the treatment of AD dementia has a synergistic effect, improving cognition and behavior for at least 1 year, and the 2-year cognitive improvement rate (MMSE ≥ 0 points) is higher than that of western medicine alone 25.64%, the exacerbation rate (MMSE ≥ 4 points) was reduced by 48.71%, and the benefit in the early stage was greater than that in the middle and late stage.  (2) Social psychology: Cognitive rehabilitation exercise treatment, patients can go to regular hospitals to receive cognitive rehabilitation exercise treatment, which can improve cognitive ability and maintain early memory, avoid continuous memory decline and prevent physical damage If the degree is aggravated, social and family psychotherapy can also be used to help improve. The society provides platforms and conditions such as public welfare organizations and exchange venues for patients, and provides better care and nursing for such patients to avoid bedsores and lung infections. and other complications, so that patients can receive fine care in daily life, improve the quality of life, and can also actively receive nutritional support treatment if necessary.  (3) Nursing:  Provide patients with a comfortable and quiet rest environment, keep the ward clean, with suitable temperature and humidity, and sufficient light. Take the medicine according to the doctor's advice, and observe the curative effect and adverse reactions. If necessary, often help the patient turn over, keep the bed sheets and skin clean and dry, change clothes frequently, massage the skin of the pressure part, and prevent pressure sores. Assist patients with physical and language rehabilitation exercises, walk with patients or do some appropriate sports. Follow the principles of the Mediterranean diet. Instruct patients to listen to some relaxing music and actively communicate with patients. Understand and support the patient spiritually, avoid anxiety and impatience, and be patient with the sick elderly just like a child who cannot speak. The behavior of Alzheimer's patients is relatively slow, so we should treat them patiently, not allow them to be greatly stimulated, and maintain a good mood with them. Drug treatment can improve cognitive function, improve mental symptoms, Psychosocial treatment and good nursing care are important to delay the decline in the quality of life of patients. |

| Question 7: What considerations are there for pharmacological treatment in patients with Alzheimer's disease, and how can medication-related adverse effects be minimized? |
| --- |
| GPT3 Chinese version:  Alzheimer's disease is an incurable neurodegenerative disease, and drug therapy is currently one of the main means of controlling the disease. Drug therapy usually includes two classes of drugs: cholinesterase inhibitors and N-methyl-D-aspartate (NMDA) receptor antagonists. These drugs can slow the progression of the disease and improve a patient's cognitive function, behavior, and activities of daily living.  Drug treatment needs to pay attention to the following issues:  1. Regular follow-up visits and examinations are required during treatment to monitor changes in the condition and the occurrence of side effects of the drug.  2. Use the medicine as directed by the doctor, and do not change the dose or stop the medicine at will. If you need to adjust the treatment plan, you should discuss it with your doctor.  3. Different drugs have different responses to patients, and the appropriate drug should be selected according to the specific conditions of the patient.  4. Pay attention to the side effects of the drug, such as nausea, vomiting, headache, insomnia, etc. If serious side effects occur, the doctor should be informed promptly and the drug should be stopped.  5. Avoid simultaneous use with other drugs, especially sedatives, antidepressants and antipsychotics, so as not to cause adverse reactions.  To avoid side effects of medicines, the following measures can be taken:  1. Carry out drug treatment according to the doctor's advice, and do not change the dose or stop the drug at will.  2. Eat a healthy diet and avoid bad eating habits such as high fat, high sugar, and high salt, as well as bad living habits such as drinking and smoking.  3. Maintain a good lifestyle, including proper exercise, good sleep and a regular schedule.  4. Regular medical check-ups and examinations for early detection and treatment of potential health problems.  5. Keep in close contact with the doctor during the treatment, and report any adverse reactions and changes in symptoms in a timely manner. |
| GPT3 English version:  Alzheimer's disease is a progressive neurodegenerative disorder that affects memory, cognitive function, and behavior. Pharmacological treatment for Alzheimer's disease aims to improve symptoms and slow down disease progression. When considering pharmacological treatment for Alzheimer's disease, several factors need to be taken into account, including the severity of symptoms, age, and medical history of the patient.  There are currently four FDA-approved drugs for the treatment of Alzheimer's disease: donepezil, rivastigmine, galantamine, and memantine. These drugs work by increasing the availability of neurotransmitters in the brain or by regulating glutamate receptors. These medications may be used alone or in combination, depending on the patient's condition.  When administering these medications, it is essential to start at the lowest effective dose and gradually increase it to minimize adverse effects. Adverse effects associated with these medications include nausea, vomiting, diarrhea, dizziness, headache, and insomnia. These side effects can be minimized by taking the medication with food, reducing the dose, or switching to another medication.  It is also crucial to monitor patients for drug interactions, especially those taking multiple medications. Medications that may interact with Alzheimer's disease medications include anticholinergics, anticonvulsants, and opioids.  In addition to medication, non-pharmacological interventions such as cognitive stimulation, physical activity, and social engagement may help manage symptoms and improve overall quality of life for patients with Alzheimer's disease.  Overall, pharmacological treatment for Alzheimer's disease should be individualized to each patient's specific needs and medical history. Healthcare professionals should monitor patients closely for adverse effects and adjust treatment plans accordingly. |
| GPT4 Chinese version:  For patients with Alzheimer's disease, drug therapy mainly consists of two classes of drugs: cholinesterase inhibitors (such as donepezil, rivetium bromide, and gazlamine) and NMDA receptor antagonists (such as meloxime) general). The main purpose of drug treatment is to slow down the progression of the disease, improve cognitive function, and reduce the severity of symptoms. When using drug therapy, the following issues need to be paid attention to:  Follow your doctor's advice: Make sure to follow your doctor's advice and prescriptions, and take your medicines at the dose and when prescribed by your doctor. Do not increase or decrease the dose, or change the medication regimen without authorization.  Observe side effects: The drug may produce certain side effects, such as nausea, vomiting, diarrhea, loss of appetite, insomnia, etc. Patients or family members should closely observe these side effects and report to the doctor in time if problems arise.  Avoid drug interactions: Patients with Alzheimer's disease may take multiple drugs at the same time, so it is necessary to pay attention to drug interactions. Inform your doctor of all medicines you are taking so that your doctor can assess the risk of potential drug interactions.  Regular review: Regular follow-up with the doctor to evaluate the effect of drug treatment and the progress of the patient's condition. Doctors may adjust the drug dose or regimen based on the patient's response.  Diet and lifestyle: Maintaining a good diet and lifestyle, such as getting enough sleep, exercising, and socializing, can improve the overall quality of life for people with Alzheimer's disease.  Family support: The support of family members is crucial for people with Alzheimer's disease. Help patients manage medications, monitor side effects, and provide emotional support when needed. |
| GPT4 English version:  Pharmacological treatment for patients with Alzheimer's disease aims to manage symptoms, slow disease progression, and improve quality of life. However, each patient is unique, and there are several factors to consider when prescribing medications. In order to minimize medication-related adverse effects, it's essential to take a patient-centered approach, which includes:  Assessing severity and stage of the disease: Alzheimer's progresses in stages, from mild to severe. Medications are typically more effective in the early stages, and treatment choices may vary depending on disease severity.  Evaluating comorbidities: Many Alzheimer's patients have other medical conditions (e.g., hypertension, diabetes, depression) that may influence drug choice, dosage, and potential interactions.  Considering patient age and overall health: Older adults may have a higher risk of adverse effects due to age-related changes in drug metabolism and clearance. Monitoring kidney and liver function is important to ensure safe drug use.  Reviewing current medications: Assessing all medications a patient is taking (including over-the-counter drugs and supplements) is crucial to minimize drug-drug interactions and potential adverse effects.  Tailoring treatment to individual needs: Patients may respond differently to medications, so it's essential to monitor their response and adjust treatment accordingly. Starting with low doses and titrating slowly can help minimize side effects.  Monitoring cognitive, behavioral, and functional changes: Regular assessments of cognitive, behavioral, and functional status can help evaluate treatment effectiveness and identify any emerging adverse effects.  Educating caregivers and patients: Providing information about potential side effects, drug interactions, and the importance of adherence can help minimize adverse outcomes.  Encouraging non-pharmacological interventions: Combining medication with non-drug approaches (e.g., cognitive stimulation, physical activity, and social engagement) can help optimize patient outcomes.  Regular follow-ups: Frequent monitoring and follow-up appointments are necessary to assess treatment response, side effects, and any needed adjustments.  In summary, a comprehensive approach that considers each patient's unique circumstances, actively monitors their response to treatment, and integrates pharmacological and non-pharmacological strategies can help minimize medication-related adverse effects in Alzheimer's disease patients. |
| Neurologist 1:  Precautions for drug treatment: People with Alzheimer's disease usually need help to take their medicines, and early-stage patients who live alone also need to call regularly for reminders. Medication storage boxes can be used to facilitate the pre-dispensing of daily doses of medicines to avoid misuse; some medicine boxes have timed reminders, which busy caregivers may also need to help remind medications. Effectively avoid drug side effects: 1) use the lowest possible dose; 2) reduce the use of quick-acting drugs. |
| Neurologist 2:  Drug treatment should follow the principle of "early diagnosis, timely treatment, and lifelong management". For the mental and behavioral symptoms associated with "dementia", non-drug intervention is the first choice, anti-"dementia" treatment is basic, and psychotropic drugs can be used when necessary, but the efficacy and side effects should be evaluated regularly, and long-term use should be avoided. Common side effects of drugs that improve cognition and antipsychotic symptoms include diarrhea, nausea, sleep disturbance, liver and kidney dysfunction, and the more serious side effect is bradycardia. In order to avoid side effects, it should be done: slowly increase the dose, regularly review liver and kidney function, electrocardiogram, etc. |
| Neurologist 3:  1) Start with a small dose: The medication for Alzheimer's disease must be individualized, and drugs cannot be used blindly. At present, clinically, the tolerance of Eastern people to drugs is much lower than that of Westerners, so they should be more cautious when using drugs, avoid one-time use of large doses, and it is best to start with small doses to avoid irritation.  2) Strict detection of adverse reactions after taking the medicine: most patients will have different degrees of adverse reactions after taking the medicine. Blind use of drugs will lead to dizziness and nausea side effects while the patient cannot control the condition, so patients must pay close attention to physical changes. If you have any adverse reactions within three days of taking the medicine, you must report the situation to your doctor in time.  3) The dosage should be increased step by step: if there is a certain effect after taking the medicine and the symptoms are slightly under control, the patient can slowly increase the dosage of the medicine with the help of the doctor. But you must not change the dosage of the medicine without authorization, otherwise it will easily affect the therapeutic effect.  4) Regular follow-up visits: During the treatment of Alzheimer's disease, a good habit of regular follow-up visits should be developed so that the condition can be better controlled. |
| Neurologist 4:  The treatment of Alzheimer's disease requires attention : ① Early diagnosis, timely treatment, and lifelong management.  ②Although the existing anti-Alzheimer's disease drugs cannot reverse the disease, they can delay the progress, and long-term treatment should be adhered to as much as possible. ③ For the psychobehavioral symptoms associated with dementia, non-drug intervention is the first choice, anti-dementia treatment is the basic, and psychotropic drugs can be used when necessary, but the efficacy and side effects should be evaluated regularly, and long-term use should be avoided. ④Health education, psychological support and practical assistance to caregivers can improve the quality of life of Alzheimer's patients.  In order to avoid the corresponding side effects of drugs, doctors need to be familiar with the usage, dosage and corresponding side effects of these drugs:  Drugs that improve cognition.  Memantine acts on the glutamate-glutamine system in the brain and is a non-competitive N-methyl-D-aspartate antagonist with moderate affinity. The usage is the initial dose of 5 mg, increase the dose to 10 mg in the second week, increase the dose to 15 mg in the third week, and increase the dose to 20 mg in the fourth week, once a day, orally. For patients with impaired renal function, the dose of memantine should be reduced accordingly. For patients with moderate or severe Alzheimer's disease, the combination therapy of a cholinesterase inhibitor and memantine can achieve better cognition, daily living ability and social function, and improve mental and behavioral symptoms. On November 2, 2019, the State Drug Administration conditionally approved Mannate Sodium Capsules for the treatment of mild to moderate Alzheimer's disease.  Non-drug interventions for psychobehavioral symptoms  Non-drug interventions for psychobehavioral symptoms emphasize people-centered. The use of nonpharmacologic interventions can promote and improve functioning, promote social and physical activity, increase intellectual stimulation, reduce cognitive problems, manage behavioral problems, resolve family conflict, and improve social support.  Patient-oriented non-drug intervention methods include environmental therapy, sensory stimulation therapy, behavioral intervention, music therapy, soothing therapy, aromatherapy, recognition therapy, and cognitive stimulation therapy. Supportive interventions for caregivers are equally important. Special attention should be paid to individual characteristics when formulating and implementing non-pharmaceutical intervention techniques.  Drug therapy for psychobehavioral symptoms  It is mainly used to control severe hallucinations, delusions and excited impulse symptoms. The use of antipsychotic drugs should follow the principle of "starting with a small dose, slowly increasing according to the treatment response and adverse reactions, and slowly reducing the dose until the drug is stopped after the symptoms are controlled". Commonly used drugs include risperidone, olanzapine, and quetiapine.  of risperidone is 0·25-0·50 mg/d, the maximum dose is 2 mg/d, divided into 1-2 doses;  Olanzapine 1·25-2·50 mg/d, the maximum dose is 10 mg/d, divided into 1-2 doses;  Quetiapine 12·5mg/d, the maximum dose is 200mg/d, administered in 1 to 3 times.  For the elderly (usually over 85 years old), 1/2 of the recommended dose can be selected as the starting dose.  It is mainly used to treat depression, mild agitation and anxiety.  Commonly used drugs such as trazodone (25-100 mg), sertraline (25-100 mg), citalopram (10-20 mg, pay attention to QTc interval), mirtazapine (7·5-30 mg), etc. May relieve symptoms such as impulsiveness and agitation. Commonly used drugs such as sodium valproate (250-1000 mg). |

| Question 8: What types of nursing and care are necessary for patients with Alzheimer's disease? |
| --- |
| GPT3 Chinese version:  Alzheimer's disease is a progressive degenerative disease that causes symptoms such as cognitive decline and emotional instability. This disease can be challenging for patients and their families and friends. Here are the types of care and care people with Alzheimer's disease need:  1. Monitoring and managing medical problems: People with Alzheimer's disease need to undergo routine medical tests to monitor the progression of the disease and manage any associated medical problems. This includes regular checks of blood pressure, blood sugar, blood lipids, hemoglobin A1C and other biological indicators, as well as receiving medication and other treatment measures.  2. Promotes social interaction: Social interaction is very important for people with Alzheimer's disease because it helps keep the brain active. Family members and caregivers can communicate with patients, play games, listen to music, and more.  3. Provide a nutritious diet: People with Alzheimer's disease need a nutritionally balanced diet to stay healthy. Nurses should ensure that patients are eating and hydrating adequately and monitor their weight.  4. Provide safety protection: Because people with Alzheimer's disease may forget basic safety common sense, there is a need for safety protection. Nurses should ensure the safety of the patient's living environment, and regularly check the use of anti-theft door locks, electrical appliances, gas and other equipment.  5. Manage Behavioral Problems: People with Alzheimer's disease may experience behavioral problems such as loss of control, depression, irritability, etc. Nurses need to understand the patient's emotions and behavior and take appropriate steps to manage these issues, such as providing a quiet environment and scheduling regular activities and socializing.  6. Provide daily care: People with Alzheimer's disease may need help with daily activities such as bathing, dressing, and eating. Nursing staff should provide appropriate assistance to ensure that patients can maintain self-hygiene and activities of daily living.  7. Promote exercise and physical activity: People with Alzheimer's disease can benefit from appropriate exercise and physical activity, such as walking, yoga, dancing, etc. These activities can help patients keep their bodies and minds active and enhance their emotional state.  8. Provide psychological support: Alzheimer's patients may feel depressed, anxious, lonely and other emotions, and need family members and caregivers to provide emotional support and comfort, and encourage them to participate in social activities to alleviate the negative effects of these emotional problems Influence.  9. Provide transportation services: People with Alzheimer's disease may need to travel regularly to the hospital or other treatment facilities for treatment, and they may not be able to make these trips on their own. Nurses can provide transportation to help patients travel to hospitals and other locations.  10. Provide medication management: People with Alzheimer's disease usually need to take medication on a regular basis, such as sedatives, antidepressants, etc. Nursing staff should ensure that patients take their medicines correctly according to the doctor's instructions, and purchase and refill needed medicines in a timely manner.  People with Alzheimer's disease require comprehensive care and care, including aspects such as medical management, social interaction, nutritious diet, safety protection, management of behavioral problems, daily care, exercise and physical activity, psychological support, transportation services, and medication management. This care and care should be a collaborative effort between family members and professional caregivers to provide the best possible support and care. |
| GPT3 English version:  Alzheimer's disease is a progressive neurological disorder that affects memory, thinking, and behavior. As the disease progresses, patients require increasing levels of nursing and care. The types of nursing and care that are necessary for patients with Alzheimer's disease include:  Personal care: Patients with Alzheimer's disease may need help with personal care tasks such as bathing, dressing, grooming, and toileting.  Medication management: Patients with Alzheimer's disease may have difficulty remembering to take their medications or may require assistance with administering medications.  Nutritional support: Patients with Alzheimer's disease may have difficulty with eating or swallowing, or may require specialized diets to manage their condition.  Cognitive stimulation: Activities that help stimulate the brain and keep patients mentally engaged, such as puzzles or memory games, may help slow the progression of Alzheimer's disease.  Safety monitoring: Patients with Alzheimer's disease may be at risk for falls, wandering, or other safety issues, and may require constant monitoring to ensure their safety.  Emotional support: Patients with Alzheimer's disease may experience feelings of confusion, anxiety, or depression, and may require emotional support and reassurance from caregivers.  Hospice and end-of-life care: In the later stages of Alzheimer's disease, patients may require hospice and end-of-life care to manage pain and provide comfort.  It is important for caregivers to have specialized training and expertise in caring for patients with Alzheimer's disease to provide the best possible care and support for these patients. |
| GPT4 Chinese version:  Alzheimer's disease is a progressive neurodegenerative disorder characterized by cognitive decline, memory loss, and behavioral and emotional disturbances. Patients require comprehensive, meticulous, patient care and attention. Here are some suggestions for people with Alzheimer's disease:  Safety Nursing: Ensure that the living environment of patients is safe and free from hidden dangers, such as preventing falls and electric shocks. Home security should be checked regularly and emergency contacts should be provided to patients.  Life care: Assisting patients with activities of daily living, such as eating, washing, dressing, going to the toilet, etc. Also pay attention to the patient's personal hygiene, bathe and change clothes for the patient regularly.  Health management: ensure that patients visit the doctor and take medicine regularly. Observe changes in symptoms and report to your doctor. At the same time, pay attention to the diet and nutrition of patients, and provide a balanced and healthy diet.  Cognitive Stimulation: Cognitive training and memory exercises such as reading, puzzles, playing cards, etc. Stimulate the patient's thinking ability and delay the progression of the disease as much as possible.  Emotional support: Give patients sufficient care and companionship, and listen to their emotional demands. Patients are encouraged to stay in touch with family, friends, and participate in social activities.  Behavioral Intervention: Provide appropriate interventions for emotional and behavioral problems such as anxiety, depression, and irritability that may occur in patients. Seek help from a psychotherapist if necessary.  Family education: training family members and caregivers to understand the characteristics of Alzheimer's disease, nursing skills, etc. Make it better to take care of patients and improve the quality of care.  Seek professional support: If home care is difficult to meet the patient's needs, consider seeking help from a professional nursing facility or day care center.  Caring for someone with Alzheimer's disease requires patience, love and expertise. Maintaining good communication with patients and respecting their needs and dignity is key to caring for someone with Alzheimer's disease. Here are some suggestions to help you take better care of your patients:  Customized Individualized Plans: Develop individualized care plans based on the patient's course of illness, needs, and preferences. Every patient's condition and needs are unique, requiring individualized care.  Maintain a regular schedule: Make sure the patient has a regular schedule, including sleep, meals, and activities. Regular lifestyle habits can help reduce anxiety and confusion in patients.  Encourage exercise: Encourage patients to take appropriate exercise, such as walking, yoga, Tai Chi, etc. Exercise helps maintain good health, improves quality of life, and relieves anxiety and depression.  Create a comfortable environment: Create a quiet and comfortable living environment for patients. Avoid excessive noise and stimulation, while ensuring plenty of natural light and ventilation.  Utilize memory aids: Use sticky notes, calendars, family photo albums, and more to help patients identify times, places, and loved ones. These tools can reduce confusion and anxiety for patients.  Prevent excessive fatigue: Avoid prolonged complex or highly focused activities that can cause excessive fatigue. Maintaining proper rest can help relieve the patient's stress.  Community resources: Find local Alzheimer's disease support groups and related services such as rehabilitation classes, counseling services, and more. These resources can help you learn more about caring and expand your support network.  In conclusion, caring for someone with Alzheimer's disease requires attention to their quality of life, patience and love. Make sure they spend each day in a safe, comfortable environment and seek out professional support and resources to help patients and families navigate this challenge. |
| GPT4 English version:  Caring for patients with Alzheimer's disease can be challenging, as the progressive neurological disorder affects memory, cognition, and behavior. Nurses and caregivers must be patient, compassionate, and knowledgeable to provide optimal care. Some types of nursing and care necessary for Alzheimer's patients include:  Personal care: Assistance with activities of daily living (ADLs) such as bathing, grooming, dressing, toileting, and feeding, as the patient's ability to perform these tasks declines.  Medication management: Ensuring proper administration of prescribed medications, monitoring side effects, and adjusting dosages as needed under the guidance of a healthcare professional.  Nutrition and hydration: Providing balanced meals and encouraging fluid intake, while being mindful of any dietary restrictions or swallowing difficulties the patient may have.  Physical activity: Encouraging regular exercise and mobility to maintain strength, flexibility, and overall well-being.  Cognitive stimulation: Engaging the patient in mentally stimulating activities, such as puzzles or memory games, to help slow cognitive decline.  Social engagement: Facilitating social interaction and participation in activities to maintain emotional well-being and reduce isolation.  Emotional support: Providing empathetic and compassionate care, understanding that patients may experience frustration, anxiety, or depression as their condition progresses.  Safety supervision: Ensuring the patient's environment is safe and free from hazards, while also monitoring for wandering and other potentially dangerous behaviors.  Communication: Adapting communication strategies to accommodate the patient's cognitive abilities, such as using simple language, speaking slowly, and maintaining eye contact.  Care coordination: Collaborating with a multidisciplinary team of healthcare professionals, such as physicians, therapists, and social workers, to develop and implement a comprehensive care plan tailored to the patient's individual needs.  Education and support for family members: Providing guidance and resources to help families understand and cope with the challenges of Alzheimer's disease, as well as assisting them in planning for future care needs.  End-of-life care: As Alzheimer's progresses, patients may require palliative care or hospice services to ensure comfort and dignity during the final stages of the disease. |
| Neurologist 1:  ① Pay attention to the personal safety of the patient; ② Pay attention to the communication with the patient; ③ Arrange the daily activities of the patient; ④ Arrange the diet of the patient reasonably. |
| Neurologist 2:  1) Understanding their difficulties Alzheimer's disease is a long process, and patients change dramatically along the way. From early stage to late stage, patients show different symptoms in each stage. Understanding the symptoms of each stage can better improve nursing work: mild Alzheimer's disease (early stage): patients at this stage have the ability to live independently, but there will be some difficulties: forgetting names and words: unable to speak name, and couldn't find the correct wording. Mild to moderate Alzheimer's disease (early to mid-stage): People at this stage experience the following difficulties: Task difficulty: Difficulty completing social or work tasks. Dyslexia: After reading an article, having difficulty remembering what it contains. Misplacement: Losing or misplacing valuables. Loss of Execution: Decreased ability to plan or organize. Moderate Alzheimer's disease (intermediate stage): Moderate Alzheimer's disease is the longest-lasting stage , usually lasting many years. At this stage, the patient's symptoms are more obvious, mainly including: loss of remote memory: forgetting things experienced in the past, and impairment of episodic memory. Mood changes: irritability is a prominent feature. Time and space confusion: not sure where they are and the exact date. easy to get lost. Personality changes: Suspicious, paranoid, and obsessive-compulsive, repeating certain actions, such as rubbing hands or tearing toilet paper. Sleep disturbance: sleeping during the day, restless at night ("sunset syndrome"). Hallucinations (taking yourself in the mirror as a stranger); hallucinations (seeing deceased relatives). Severe Alzheimer's disease (late stage): People at this stage: Unaware of their surroundings. Unable to respond, unable to have a conversation (only words). Unable to control actions. Needing help with meals or toileting. Lost smile, couldn't sit down by himself, couldn't look up. Reflexes become abnormal. Muscles become stiff. Decreased swallowing ability. Susceptibility to disease, especially pneumonia.  2) Understand their anxieties. Anxiety increases cognitive load and may also lead to noncooperation, making caregiving more difficult. Understanding the source of their anxiety and avoiding it can better improve the nursing work.  3) Planning daily activities and providing training stimuli Different from other nursing care, Alzheimer's disease nursing work also needs to pay attention to providing cognitive and sensory stimuli. A 2014 paper (Watchman, 2014) provides examples of stimulating activities that can be done at home, which are listed here: Early: board games, card games, beanbag shuttlecocks or soft balls thrown at each other, massage or aromatherapy, arts and crafts Activities, cooking chores. Early and mid-term: music (strong beat + bass is also acceptable), (sitting on a chair) dancing hands and feet, kneading clay, old photos (memories), narrative (reminiscence narrative), smell and taste (sensory stimulation), VR virtual vision (sensory activation), specific tasks (such as walking around the park along the wall), bubble bath (sensory activation). Middle stage: folding clothes, multisensory stimulation (sound, light, smell), simple kitchen chores (such as peeling, stirring, etc., simple task), simple gardening (also simple task, such as pulling weeds) Late stage: laughing (helping them), touch (their hands), sing (help them hum), rock (their seat). Expose them to plush toys (anxiety-reducing), task blankets. (Task blanket: unbutton, zip, put items into small pockets) If the patient's condition is sufficient, it is advisable to provide some more challenging tasks and give more intensive cognitive training, such as recalling TV details. For example, when an elderly person watches TV, family members can discuss with him, what was being said on TV just now? Encourage him to recall as many details as possible. memorize vocabulary.  4) To communicate effectively, master ten skills Learn how to communicate correctly, the ten skills are listed below: Don't say "Do you remember?" or "How did you forget": their memory is impaired, remind it repeatedly, will only speed up their damage and will not help improve memory. Agree with everything, don't argue with them: Debates can lead to stress and unhappiness, neither of which are good for Alzheimer's patients. Slow down, speak clearly, and speak well. When asking about their intentions, use yes-or-no sentences (“May I get you a cup of tea now, please?”) rather than open sentences (“What would you like to drink?”). Say one thing at a time, ask one thing at a time. More cooperation with body language (such as more hand gestures, more "little movements") can help them understand. Let them see you squarely, don't be condescending, and don't talk behind their backs. Before you move on to the next action, you need to speak up (don't do something without warning). Encourage them to use objects to tell stories. Invite their input and encourage their participation. "  5) Arranging interior furnishings and helping them move comfortably Alzheimer's patients have certain obstacles, so they often get lost, confused, and even have accidents. These particular obstacles include: - Loss of depth: e.g. the edge of a stair (ladder) cannot be discerned. - Loss of color perception: blue is not easily discernible. Experts suggest some interior decorating tips to help people with Alzheimer's move comfortably. (1) Tables and chairs, make good use of contrasting colors: The key point of the arrangement of tables and chairs is to show the boundaries. Therefore, more vivid contrasting colors should be used to help patients identify the edge range. (Contrast the furnishings with large color differences to help identify the edges) (2) Floors or carpets, avoiding complexity: The focus of the layout of floors and carpets is to avoid confusion and avoid causing patients' fear. For example, a checkerboard floor (carpet) can cause confusion and be detrimental to the patient. (Plaid carpets cause confusion, and the floor layout should be simplified). (3) Tableware: Brightly colored cups and plates seem to help increase the diet of people with advanced dementia. One study showed that patients ate 24.6% more when changing white tableware to bright red. (4) Bedroom: Ten furnishing tips. 1- Display family photos: It can help maintain memory and avoid strangeness. 2-Bed, quilt and floor: different colors to help patients identify and separate. 3-Floor (carpet): not complex. 4- Wardrobe: Keep only a few pieces and avoid too many options. 5-Cabinet: display familiar items to increase the sense of security. 6- Night lighting. 7-Clock: Help remind time. 8-Wall Switch: Easy to find. 9- Restroom: remove the door (no door), help patients find it. 10-Ceiling lights: use ceiling lights for lighting instead of standing lights (shadows cast sideways on the ground will cause illusions). (5) "Benign noise" theory: For Alzheimer's patients, a stable and predictable environment is important, but small surprises are also beneficial. Some scholars (Morgan and Stewart, 1999) also pointed out that moderate environmental stimulation can bring benefits. These benefits include: triggering associations, encouraging socialization, and activating sensory abilities. Conclusion Special reminder from experts: In addition to caring for patients with Alzheimer's disease, caregivers should pay attention to their own physical and mental health. Find ways to increase self-care, stay positive, and connect with others. |
| Neurologist 3:  1) For those whose comprehension or executive ability has declined, family members should give more help to avoid wearing reverse clothes and forgetting keys when going out;  2) Family members should give care to those with reduced living ability, and avoid patients from not eating or being full;  3) For long-term bedridden patients, the family members should regularly turn over the patient, pat the back and massage the skin. Families with conditions can replace the air mattress for the patient, so as to avoid the occurrence of pressure sores. Foods rich in protein, lecithin, and cellulose can improve intelligence and promote intestinal peristalsis. |
| Neurologist 4:  Appropriate care can slow progression and improve quality of life in people with Alzheimer's disease. Therefore, families with Alzheimer's disease patients should consider the four aspects of patients, caregivers, living environment and resources during home care, and formulate a comprehensive personalized care plan.  (1) For the elderly who are able to move, caregivers can arrange for the elderly to do some things within their ability, such as going to the toilet regularly, washing, dressing and undressing, etc., and encourage and assist the elderly to participate in activities they are interested in, including playing chess, poker, reading aloud, singing, playing games, etc., let the elderly gain a sense of existence, value and satisfaction in entertainment activities.  (2) The caregiver needs to know the relevant knowledge about Alzheimer's disease, communicate with the patient in a calm tone, listen patiently, and understand the living habits, hobbies and hobbies of the elderly. At the same time, the caregiver should take the medicine according to the dose, supervise the taking of the medicine, and prevent missed doses, excessive doses, and excessive doses. Caregivers need to master the ability to deal with abnormal mental behavior of patients. When the patient has behaviors such as fright, anxiety, and violence, the caregiver should identify it in time, comfort and persuade appropriately, and divert the attention of the elderly.  (3) The patient's home environment should be comfortable, safe and well lit. The room should have fewer thresholds and more handrails, and the floor should be non-slip to prevent patients from falling. Caregivers also manage dangerous items in the home to prevent accidental injuries. The door should be equipped with a combination lock that is not easy to open to prevent patients from getting lost. Try to do less moving, less decoration, and maintain the stability of the living environment of patients.  (4) Caregivers can make full use of family resources and social resources. Ask relatives, friends, etc. to help take care of the elderly and relieve their own pressure. At the same time, caregivers can also keep in touch with day care centers, communities, hospitals, etc., and get support and help in a timely manner. Special attention should be paid to the fact that caregivers should avoid excessive care when caring for Alzheimer's patients, otherwise the rate of loss of residual function of the patient will be accelerated. |

| Question 9: What strategies can be used to provide nutritional support for patients with Alzheimer's disease? |
| --- |
| GPT3 Chinese version:  Nutritional support for patients with Alzheimer's disease should be individualized on an individual basis. Here are some general recommendations that may apply to many patients:  Get enough protein: Protein is essential for proper body function and health. People with Alzheimer's disease should get enough protein to help maintain muscle mass and immune function.  Get enough vitamins and minerals: People with Alzheimer's disease should have enough vitamins and minerals in their diet, especially folic acid, vitamins B6 and B12, and vitamin D. These nutrients can help maintain the health and function of the nervous system.  Get enough fiber: People with Alzheimer's disease should have enough fiber in their diet to help maintain gut health. Dietary fiber can be ingested by eating fruits, vegetables, whole grain bread, oatmeal and other foods.  Control fat intake: Alzheimer's disease patients should control fat intake, especially saturated fat and trans fat intake. These fats may increase the risk of cardiovascular disease.  Maintain an appropriate weight: People with Alzheimer's disease should maintain an appropriate weight to help maintain good health and function. Being overweight or underweight can have negative effects on the body.  Eat a varied diet: People with Alzheimer's disease should eat a varied diet, including eating a variety of proteins, vegetables, fruits, whole grains, low-fat dairy products, and healthy fats.  Eating regularity: People with Alzheimer's disease should maintain a regular eating habit, including eating regularly and avoiding meal skipping. This helps maintain good health and improves the body's ability to absorb nutrients  Increase water intake: People with Alzheimer's disease often suffer from memory loss, so they may forget to drink water. However, adequate water intake is necessary to maintain good health and help the body metabolize. Drinking plenty of water every day is recommended, especially during hot weather or intense activity.  Avoid excessive alcohol consumption: People with Alzheimer's disease should avoid excessive alcohol consumption. Excessive alcohol consumption may damage the nervous system and increase the risk of Alzheimer's disease.  Simple diet: The diet of people with Alzheimer's disease should be as simple as possible. Overly complex food preparation and cooking may increase patient discomfort and difficulty. Therefore, choose simple foods such as protein sources that are easy to chew and digest, and vegetables and fruits that are grilled or steamed.  Nutritional support for patients with Alzheimer's disease should be a comprehensive program that takes into account the patient's individual circumstances and special needs. Patients and their families can seek help from a medical professional, dietitian, or other professional to develop a personalized nutrition plan. |
| GPT3 English version:  Patients with Alzheimer's disease (AD) may experience difficulty with eating and maintaining proper nutrition due to a variety of factors such as cognitive decline, loss of appetite, and physical limitations. Therefore, providing adequate nutritional support is essential for the management of the disease. Some strategies that can be used to provide nutritional support for patients with Alzheimer's disease include:  Encouraging small, frequent meals: Rather than three large meals per day, encourage patients with AD to eat smaller, more frequent meals. This approach can help prevent feelings of fullness and reduce the likelihood of gastrointestinal discomfort.  Offering nutrient-dense foods: Provide foods that are high in nutrients such as lean protein, whole grains, fruits, and vegetables. These foods can help maintain overall health and provide energy for daily activities.  Ensuring adequate hydration: People with AD may forget to drink water or may not feel thirsty. Therefore, caregivers should ensure that patients with AD are drinking enough fluids to stay hydrated.  Providing familiar foods: People with AD may experience a decreased interest in food, so providing familiar and enjoyable foods can help stimulate appetite and promote eating.  Using food fortification: Fortifying food with additional nutrients, such as protein powder, can help increase the nutritional content of meals and improve overall nutrient intake.  Consulting with a dietitian: A registered dietitian can assess the nutritional needs of patients with AD and develop a personalized nutrition plan. They can also offer guidance on food preparation and provide recommendations on supplements if needed.  Overall, the goal of nutritional support for patients with Alzheimer's disease is to maintain proper nutrition and prevent malnutrition, which can worsen symptoms of the disease. |
| GPT4 Chinese version:  Alzheimer's disease is a common neurodegenerative disorder that primarily affects older adults. Providing proper nutritional support to patients is critical to maintaining their health and quality of life. Here are some suggestions for nutritional support for people with Alzheimer's disease:  Balanced diet: Make sure the patient eats a balanced diet that includes the right amount of protein, carbohydrates, fat, fiber, vitamins, and minerals. It is recommended to eat more fruits, vegetables, whole grains, low-fat dairy products, fish and lean meats.  Increase your antioxidant intake: Antioxidants can help reduce oxidative stress, which may be linked to the development of Alzheimer's disease. Antioxidant-rich foods include dark-colored fruits and vegetables such as blueberries, strawberries, spinach, and kale.  Consume healthy fats: Foods rich in omega-3 fatty acids, such as fish (salmon, tuna, sea bass, etc.), nuts, and seeds, help support healthy brain function. At the same time, the intake of trans fat and saturated fat should be limited.  Stay hydrated: Staying well hydrated is critical to brain function. Encourage patients to drink water frequently to prevent dehydration.  Follow a "Mediterranean Diet": Research suggests that a Mediterranean diet may be beneficial for people with Alzheimer's disease. This eating pattern emphasizes foods found in healthy fats (such as olive oil), fish, fruits, vegetables, whole grains, and nuts.  Food safety: Because of the cognitive impairment that may occur in people with Alzheimer's disease, it is critical to ensure food safety. Make sure all food is fresh, properly stored, and heated at the proper temperature.  Offer foods that are easy to chew and swallow: As the disease progresses, patients may have difficulty swallowing. For this, foods that are easy to chew and swallow, such as pasty foods, soft foods, and chopped foods, should be provided. Avoid dry, hard or grainy foods, which can cause coughing or choking.  Regular Meals: Provide patients with regular meals to help maintain blood sugar stability and avoid excessive hunger. If the patient cannot eat large meals, more frequent smaller meals may be considered.  Table Setting: Provides a peaceful, distraction-free environment for people with Alzheimer's disease to help them focus on eating. Avoid excessive background noise, such as television or radio, while eating.  Encourage independent feeding: Try to encourage self-feeding where appropriate, such as using easy-to-grip utensils, to maintain independence and dignity.  Closely observe the patient's dietary status: Continue to pay attention to the patient's dietary status, such as appetite, digestive problems, weight changes, etc., so that the diet plan can be adjusted in time.  Seek Professional Advice: If necessary, consult a dietitian or physician to develop an individualized meal plan to meet the patient's specific nutritional needs. |
| GPT4 English version:  Nutritional support for patients with Alzheimer's disease is crucial to maintain their overall health and potentially slow down the progression of the disease. Here are some strategies to consider:  Regular, balanced meals: Offer three balanced meals and healthy snacks daily, ensuring that they include a variety of foods rich in essential nutrients such as vitamins, minerals, fiber, and antioxidants.  Finger foods: As Alzheimer's progresses, patients may have difficulty using utensils. Offer easy-to-handle finger foods like cut-up fruits and vegetables, cheese, and sandwiches.  Modified textures: If the patient has trouble chewing or swallowing, modify the food's texture by blending, chopping, or pureeing it. Thicken liquids if needed, using commercial thickeners or natural alternatives like mashed potatoes or yogurt.  Encourage fluids: Dehydration can worsen cognitive decline. Offer water, juice, milk, and other hydrating beverages regularly, and use a marked water bottle to track daily fluid intake.  Mealtime environment: Create a calm and structured mealtime environment by minimizing distractions, using contrasting colors for plates and tablecloths, and maintaining a consistent routine.  Eating assistance: Patients may need assistance with eating. Sit beside them, make eye contact, and use simple verbal cues to encourage them to eat. You may also need to physically guide their hand to their mouth or feed them yourself.  Monitor weight and nutritional status: Regularly assess the patient's weight and nutritional status, looking for signs of malnutrition, dehydration, or unintentional weight loss.  Use of supplements: Consult with a healthcare professional to determine if any nutritional supplements, such as multivitamins, omega-3 fatty acids, or vitamin E, could be beneficial.  Dietitian consultation: Collaborate with a registered dietitian who specializes in Alzheimer's disease to develop a tailored meal plan, address specific nutritional concerns, and adjust the plan as the disease progresses.  Family and caregiver support: Educate family members and caregivers about the importance of proper nutrition and enlist their help in implementing these strategies.  Remember that every patient is unique, and their nutritional needs will change as the disease progresses. Regular assessments and adjustments to the care plan are necessary to ensure optimal nutrition and quality of life. |
| Neurologist 1:  Sufficient food; try to avoid dietary restrictions; drugs to increase appetite are not recommended. |
| Neurologist 2:  The main dish is mainly fish, meat and eggs, and the side dishes are supplemented with vitamins and minerals. Eat small meals frequently to ensure nutrition. The amount of food should be small and the variety should be more. Staple food: Rice, pasta, etc. are the main sources of energy. Rice is also an important source of protein, so it is recommended to have a meal of rice a day. Main dish: Choose protein-rich foods such as fish, meat, eggs, and soybeans. Fish and meat can not be neglected, can enhance hematopoietic function and muscle strength. Eggs and soybean products for breakfast, and fish or meat dishes for lunch and dinner can increase energy. Side dishes: vegetables, fungi, yams, and seaweeds are the main ingredients. Eat more foods rich in vitamins, minerals, and dietary fiber to keep various functions of the body normal. Dairy products and fruits: As a snack, fruits can supplement vitamins and calcium. |
| Neurologist 3:  For critically ill patients who need nutritional support, enteral nutrition should be preferred over parenteral nutrition. When starting feeding, the patient's body mass index, nutritional intake before admission, disease severity, comorbidities, and gastrointestinal function should be evaluated, and the target value, that is, the energy requirement, should be calculated. If the patient's gastrointestinal function is evaluated, early enteral nutrition should be started within 24-48 hours after admission, and the feeding goal should be reached within 48-72 hours. During the enteral nutrition process, it is necessary to monitor whether the nutrition is sufficient and the patient's tolerance, and to select an appropriate enteral nutrition formula, the concentration should be from thin to thick, and the speed of the nutrition pump should be controlled, and the infusion speed should be gradually increased. If the patient has enteral nutrition contraindications or enteral nutrition cannot be tolerated, it is expected that normal nutrition will not be possible within 3 days, and parenteral nutrition needs to be started within 24-48 hours, and the target value should be reached within 2-3 days. If enteral nutrition alone cannot achieve the target energy, parenteral nutrition should be considered after 2 days. Doctors will formulate nutrient solution according to the patient’s condition, and provide low-calorie and adequate protein supplements in the first week. Once the patient’s gastrointestinal tract can be used safely, they will gradually transition to enteral nutrition or oral diet. |
| Neurologist 4:  The nutritional problem of Alzheimer's disease may be a factor that delays the onset of the disease, and at the same time is a guarantee for maintaining the basic quality of life of the patient. The nutritional status of patients may also be related to their clinical prognosis. The scientific research results of foreign scholars have shown that insufficient intake of some nutrition-related factors such as folic acid, niacinamide, vitamin C, etc. are all related to the occurrence of Alzheimer's disease. And intake of adequate and balanced nutrients can prevent the occurrence of Alzheimer's disease. Therefore, finding and supplementing nutrients related to Alzheimer's disease is becoming one of the research hotspots in nutrition science. A study published in "Archives of Neurology" in April 2010 showed that a diet rich in nuts, fish and vegetables and low in high-fat dairy products and red meat can prevent Alzheimer's disease The occurrence of silent disease (AD). Dietary patterns were calculated based on differences in the levels of seven key nutrients most associated with AD risk documented in the literature. The results showed that a dietary pattern rich in omega-3 polyunsaturated fatty acids, omega-6 polyunsaturated fatty acids, vitamin E, and folic acid, while low in saturated fatty acids, was strongly associated with the prevention of AD. The protective effect of this dietary pattern did not change after adjusting for age, education, race, and sex. Further analysis after adjusting for smoking status, body mass index, energy intake, comorbidities, and apolipoprotein E genotype found that these effects were not significantly attenuated. Based on recent clinical evidence, nutritional factors associated with AD are as follows:  (1) Foods rich in choline and niacinamide:  Studies have linked Alzheimer's disease to the intake of choline and niacinamide. Niacinamide can stimulate cerebral blood circulation and help most AD patients improve brain cell recovery. The poor memory and learning ability of AD patients may be related to the lack of acetylcholine in the body. Choline is a B vitamin that plays multiple roles in cell membrane phospholipid metabolism. Favored for its potential role in neurological diseases. A precursor to phosphatidylserine and lecithin, phospholipids are found in the membranes of cells, including nerve cells. Because nerve impulse transmission requires choline, choline combines with acetic acid to form acetylcholine, which can cross the gaps between nerve cells and conduct nerve impulses. Lecithin is the raw material converted into choline in the brain, and people can take lecithin from food to prevent AD. In daily diet, soybean and its products, fish brain, egg yolk, pork liver, sesame, yam, mushroom, peanut, etc. are all natural foods rich in lecithin. Regular intake can provide beneficial nutrients for the brain, improve intelligence, and delay mental decline. Therefore, eating foods rich in choline and niacinamide may help AD. At present, the reference intake of choline for adults in my country is 500 mg per day, and foods rich in choline, such as eggs, egg yolks, liver, soybeans, wheat bran, cheese, barley, corn, rice, millet, beer yeast, etc., contain Niacinamide-rich foods include animal liver, kidney, and lean meat.  (2) Foods rich in folic acid and B vitamins  The occurrence of AD disease may also be related to the lack of B vitamins and folic acid. An earlier study by Goodwin et al. found a correlation between nutrient intake and cognitive function, with more cases of AD and elevated serum homocysteine levels in patients compared with controls. Folic acid and vitamin B12 can reduce the content of homocysteine in the body, so supplementing folic acid and vitamin B12 may help prevent AD. In a nine-year clinical study, researchers found that older adults who ate a folic acid-rich diet had half the risk of developing AD compared with older adults who ate less than the recommended amount of folic acid. The researchers investigated the diet of 579 volunteers (353 males, 220 females). None of them suffered from Alzheimer's disease when they were around 60 years old. After 9 years of follow-up observation, 57 people suffered from Alzheimer's disease. The researchers compared the daily nutrient intake of people with and without AD. The results showed that people whose diet was rich in folic acid had about 60% lower chance of developing AD. Folate-rich foods include oranges, bananas, green leafy vegetables, asparagus, broccoli, liver, and various beans and peas, as well as folate-fortified bread.  Although the American Heart Association does not recommend widespread use of folic acid supplements to reduce the risk of heart disease and stroke; they recommend a healthy, balanced diet that includes at least five servings of fresh fruits and vegetables per day, which ensures that Daily folic acid intake. Solfrizzi et al. also found that a high intake of monounsaturated fatty acids appeared to protect against age-related cognitive decline. Other scholars have found that the level and intake of blood vitamin C are related to individual cognitive function. Perkin et al. found that among 4809 subjects, after adjusting factors such as age and education level, it was found that the decrease of vitamin E per unit of cholesterol in serum was correlated with the increase of memory loss. Intervention studies using higher doses of vitamin E have shown some improvement in AD patients with severe dementia.  (3) Soybeans and their products  Soybean (soybean) is a traditional tonic food. Soybeans are known as the king of beans, containing about 40% to 50% protein. The protein content of 1kg of soybeans is equivalent to that of 2.5kg of lean pork or 2kg of lean beef, so some people call soybeans "green milk" or "vegetable meat". In addition to being rich in protein, soybeans also contain phospholipids, carotene, B vitamins, niacin, folic acid, choline, saponins, and various nutrients such as iron, phosphorus, calcium, and potassium. Soybeans are rich in active substances such as isoflavones, saponins, and oligosaccharides.  Scientific research has found that soybean isoflavones have a certain effect on brain health. The chemical substances in it are extremely stable, and no matter whether they are fried, boiled or stewed, their structure will not be destroyed, and their effects will not be affected. Therefore, regular consumption of soybeans can not only absorb vegetable protein, but also prevent blood lipids Abnormalities and arteriosclerosis may have certain effects in preventing AD.  Soybeans are rich in protein, phospholipids, unsaturated fatty acids, calcium and vitamins. The glutamic acid contained in its protein is one of the material bases of human brain physiological activities. Every 100g soybean contains about 6.6g glutamic acid. Its phospholipid content is also high, accounting for about 2% of the total weight. Soy phospholipids can assist in the transport of cholesterol, remove cholesterol on blood vessel walls, prevent cerebral arteriosclerosis, and prevent vascular Alzheimer's disease.  There are also many ways to eat soybeans, which can vary from person to person. As an elderly person, it can be soaked and ground into soy milk or soy milk as a breakfast drink. Soybeans can also be made into various soy products such as tofu, bean curd, yuba, dried tofu, and tempeh.  (4) Whole grains  Whole grains, which have already withdrawn from urban people's tables, are increasingly becoming people's new favorites, especially people are gradually paying attention to the so-called "health" effect of coarse grains on the brain of middle-aged and elderly people. However, evidence of effectiveness is still lacking. There are many studies on oats abroad. Oats are also known as wild wheat and brome. According to modern scientific analysis, the edible part of oats contains 15.6g of protein, 6.7g of fat, 66.9g of sugar per 100g, and the content of calcium, phosphorus, iron, vitamin B1, vitamin B2, niacin, etc. is also high. In recent years, some research results have shown that naked oats contain linoleic acid, which is extremely beneficial to the human body, so it has the effect of inhibiting the rise of cholesterol. Studies conducted by some medical scientists have shown that eating 60g of oatmeal every day can sometimes reduce the total cholesterol level of the human body by 30%. British medical scientists believe that the main reason why oatmeal can lower cholesterol is that it contains a special soluble fiber and also contains a variety of enzymes.  (5) Fish food  There are many kinds of fish, and there are more than 1,500 kinds of marine fish in China. Fish is rich in nutrition and is suitable for middle-aged and elderly people. Canadian researchers found that the content of w3 fatty acids (especially docosahexaenoic acid DHA) in the blood of healthy elderly people was much higher than that of dementia elderly people in a study of 70 elderly people (about 1/4 of whom had AD). This fatty acid is rich in deep-sea fish oil and has the function of preventing heart disease. Therefore, eating salmon properly can prevent dementia and heart disease to a certain extent. Among the fat contained in fish, unsaturated fatty acid is as high as more than 80%, and the carbon chain is longer. Therefore, eating more fish oil can reduce cholesterol, reduce atherosclerosis and vascular dementia. Fish also contains vitamin A, vitamin D, in addition to vitamin B1, vitamin B2, vitamin B12 and so on.  (6) Nut foods such as walnuts  Walnuts, also known as walnuts, have a higher proportion of monounsaturated fatty acids (58% - 74%). Can increase the nutrient supply of the brain. The trace elements and phospholipids contained in it can promote the proliferation of nerve cells. Rich in vitamin E has a strong antioxidant effect. In addition, the use of walnut kernels for so-called "brain strengthening" has a long history in China, and is also very popular in Japan and some countries in Southeast Asia.  (7) wine  Wine contains glucose and fructose and can be absorbed directly. In addition, 1 liter of wine contains 5-7g of organic acids, including lactic acid, acetic acid and so on. These organic acids can stimulate the digestive system, increase appetite, facilitate the digestion and absorption of protein and vitamins, and may reduce cholesterol accumulation. Wine also contains calcium, magnesium, iron, sulfur, tannins, anthocyanins, etc., which may be beneficial to brain function.  (8) Foods that may damage brain memory  Long-term life practice and research have shown that many foods may damage intelligence and should be avoided. Mainly by the following categories: Excessive alcohol: All alcoholic beverages contain ethanol (alcohol). If excessive drinking, it can seriously damage the brain tissue and nerve tissue, neurological disorders, and even dementia. Saccharin and high-sugar food: Saccharin is a chemical product extracted from coal tar, which contains sodium saccharin and ammonia compounds. Eating more and eating for a long time can cause neuritis and brain damage. Excessive intake of sucrose may cause vitamin B1 deficiency, increase calcium consumption, and reduce nutrients necessary for brain physiological activities, thereby causing mental decline. Therefore, sugar should be taken in according to normal needs, not too much. At the same time, simple sucrose does not contain other nutrients, and excessive consumption is not recommended. Lead-containing food and aluminum-containing food: Our daily food does not contain too much aluminum, but aluminum "traces" can often be seen in some food additives. Such as household yeast powder, cheese and soda crackers, although the amount is not large, it is worthy of the attention of the elderly. Due to the leavening agent used in the production, a small part of the food contains alum. For example, "old-fashioned" fried dough sticks and oil cakes contain high aluminum content, so it is not suitable for long-term excessive consumption. Lead-containing foods mostly refer to those foods that are processed in the streets and alleys with heated and pressurized puffers, including dried rice cakes and popcorn. Because this kind of popper contains a large amount of lead, the lead content in popcorn is relatively high, often exceeding the allowable standard amount. When lead enters the human body, it will affect the human brain cells and nervous system, and even cause poisoning.  Artificial colors and canned foods: Artificial colors and some canned foods contain preservatives. Long-term consumption may damage intelligence, so attention should also be paid.  (9) Nutrition and comprehensive factors  The comprehensive network effect of nutrients cannot be ignored. In his article published in 2010, Professor Kamphuis discussed the mechanism of the role of various nutrients such as omega-3 fatty acids, B vitamins and many antioxidant substances in the prevention and early treatment of Alzheimer's disease, suggesting a combined role of these nutrients in membrane/synaptic degeneration, abnormal protein processing (amyloid β, Tau), vascular risk factors (hypertension, hypercholesterolemia), in inflammatory responses, and oxidative stress, etc. All aspects have a regulating effect and play a positive role in the early prevention and treatment of AD. This study also provides a theoretical basis for the research and development of nutritional products for the prevention and treatment of early AD. In 2013, Nick van Wijk et al. studied the comprehensive nutritional preparation Souvenaid (including uridine, docosahexaenoic acid, eicosapentaenoic acid, choline, phospholipids, folic acid, vitamin B12, vitamin B6, C, E, selenium) products contribute to memory improvement in early AD.  In short, it is necessary to adhere to a scientifically balanced diet for a long time to prevent AD. The weight of the human brain is less than 1/40 of the total body weight, but the oxygen consumption accounts for 1/4 of the whole body's oxygen consumption. Therefore, energy satisfaction is the primary factor for brain health. Therefore, for people who do more mental work, their brains need more energy. In the daily diet, it is necessary to pay attention to the intake of staple food not lower than the body's needs to meet the sugars needed by the body.  The demand for protein by the elderly is about 1g-1·2g/(kg body weight d). If the supply is insufficient, the aging of various tissue cells in the body, including brain cells, may be accelerated. The quantity of protein is important, but the quality is also very important. The proportion of amino acids must be complete, and the content of lecithin must be rich, so as to benefit the metabolism of the brain.  Currently, there is no definite and effective treatment for Alzheimer's disease. Reasonable dietary nutrition may delay the occurrence of the disease. What is feasible is to have a balanced nutritional intake, eat plant-based protein and calcium-containing foods, supplement vitamin E and lecithin in an appropriate amount, and eat more fresh vegetables and fruits. Reduce the intake of aluminum and copper, eat less fatty meat, excessive salt and too much sugar. |

| Question 10: What are the symptoms that Alzheimer's disease patients should look out and go to the emergency room for? |
| --- |
| GPT3 Chinese version:  Alzheimer's disease is a degenerative disorder of the nervous system that primarily affects memory, thinking and behavior in older adults. Here are some symptoms that people with Alzheimer's disease should be aware of and seek medical attention if they occur:  Memory loss: People with Alzheimer's disease will gradually lose their memory, often forgetting important appointments, the names of common things, or where frequently used items are placed.  Decreased language skills: People with Alzheimer's disease will gradually decline their language skills, manifested as difficulty speaking or not being able to find the correct vocabulary.  Decreased spatial perception: People with Alzheimer's disease have decreased spatial perception and may experience things like getting lost or lost.  Loss of self-care ability: As the disease progresses, people with Alzheimer's disease may gradually lose the ability to take care of themselves, including dressing, washing, and eating.  Emotional lability: People with Alzheimer's disease may exhibit emotional lability, being easily agitated, crying, or irritable.  Insomnia or sleep disturbances: People with Alzheimer's disease may experience insomnia or sleep disturbances, having trouble falling asleep or waking up often.  If a patient with Alzheimer's disease has any one or several of the above symptoms, it is recommended to go to the hospital for medical help in time. Doctors may conduct relevant examinations and assessments, and formulate a treatment plan suitable for the patient, including drug treatment, behavioral treatment, and supportive treatment. |
| GPT3 English version:  Alzheimer's disease is a progressive neurodegenerative disorder that primarily affects memory and cognitive function. While the symptoms of Alzheimer's disease can vary from person to person, there are some general warning signs that patients and caregivers should be aware of that may warrant a visit to the emergency room. These can include:  Severe confusion or disorientation: If the person with Alzheimer's disease becomes extremely confused, disoriented, or has trouble recognizing people or places, it may be a sign of a more serious issue.  Agitation or aggressive behavior: If the person with Alzheimer's disease becomes suddenly agitated, aggressive, or exhibits other unusual behavior, it could be a sign of an underlying medical condition.  Falls or injuries: People with Alzheimer's disease are at a higher risk of falling and injuring themselves. If the person falls and appears to be in pain or has trouble moving, seek medical attention immediately.  Severe headaches: Headaches can be a symptom of a serious underlying condition, such as a stroke or bleeding in the brain. If the person with Alzheimer's disease experiences severe or sudden headaches, seek medical attention immediately.  Difficulty breathing: If the person with Alzheimer's disease has trouble breathing or appears to be struggling to breathe, seek medical attention immediately.  Seizures: Seizures can be a sign of a serious underlying condition and should be treated as a medical emergency.  It's important to remember that any sudden or significant change in behavior, mood, or physical condition should be evaluated by a healthcare professional to ensure prompt and appropriate care. |
| GPT4 Chinese version:  Alzheimer 's disease is a common neurodegenerative disease that mainly affects the cognitive function of the elderly. The following are some of the main symptoms of Alzheimer's disease. If you or someone you know has these symptoms, seek immediate medical evaluation and diagnosis.  Memory impairment: This is the most common and obvious symptom of Alzheimer's disease. Impairment of short-term memory, such as forgetting recent events or just learned information.  Difficulties with language and communication: People may become articulate, have trouble organizing their thoughts, or have trouble finding the right words and expressions.  Decreased orientation and spatial awareness: Patients may become disoriented, have difficulty recognizing familiar places, or feel confused.  Difficulty handling everyday tasks: Patients may have trouble performing familiar everyday tasks, such as cooking, managing money, or taking medications.  Impaired judgment: Patients may have problems making decisions or making judgments, such as dressing or managing money.  Decreased social activities: Patients may lose interest in previously enjoyed social activities and exhibit withdrawn, mood swings, or irritable behavior.  Mood and personality changes: Patients may experience mood swings such as anxiety, depression, suspicion, and fear.  Vision problems: In some cases, Alzheimer's disease can lead to vision problems, such as a decline in recognizing colors, assessing distance, and visuospatial perception.  If you notice any of the above symptoms in yourself or a loved one, seek medical attention promptly. Although there is currently no cure for Alzheimer's disease, early diagnosis and treatment can help slow the progression of the disease and improve quality of life. |
| GPT4 English version:  I'm not a doctor, but I can provide you with some general information about Alzheimer's disease and situations that might warrant a visit to the emergency room. Keep in mind that you should always consult with a healthcare professional for advice specific to your situation.  Alzheimer's disease is a progressive neurological disorder that primarily affects memory, thinking, and behavior. While there is no definitive list of symptoms that should prompt an immediate visit to the emergency room, some concerning situations that might require urgent medical attention include:  Severe confusion or disorientation: If the individual is suddenly unable to recognize familiar people, places, or things, or is unable to understand basic information, this could indicate a need for emergency care.  Sudden or significant change in behavior: A sudden change in mood, increased agitation, or new-onset psychosis (hallucinations or delusions) may warrant an emergency evaluation.  Inability to communicate: If the person is suddenly unable to speak or understand speech, this could be a sign of a stroke or another medical emergency.  Seizures: Seizures are not common in Alzheimer's disease but can occur in some cases. If an individual experiences a seizure, seek immediate medical help.  Falls or injuries: Falls are more common in people with Alzheimer's due to impaired balance and coordination. If the person falls and suffers a significant injury, like a head injury or fracture, seek emergency care.  Signs of infection: People with Alzheimer's can be more susceptible to infections, such as pneumonia or urinary tract infections. Symptoms like fever, difficulty breathing, or sudden worsening of confusion should prompt immediate evaluation.  Dehydration or malnutrition: If the person is not eating or drinking enough and shows signs of dehydration (e.g., dark urine, dry mouth, sunken eyes) or malnutrition, seek medical attention.  These are just a few examples of situations that may require emergency care for someone with Alzheimer's disease. It is essential to consult with a healthcare professional for personalized advice and to establish a plan for managing Alzheimer's-related emergencies. |
| Neurologist 1:  A. Significant memory loss, repeating words that have been said many times, forgetting whether to eat or take medicine, forgetting to turn off the gas, etc.  B. Confusion of time, place or relationship between people, getting lost or getting lost in a familiar place, or being pretentious to very familiar relatives and friends.  C. Significant decline in judgment, misjudgment of the relationship between weather and clothing, wearing cotton clothes in midsummer, and unlined clothing in cold winter. It is easy to be misled and deceived.  D. Mood fluctuates greatly, without any reasonable reason, there is a rapid change of mood; such as from joy to crying within a few minutes, or joy when it should be sad.  E. There is a change in personality, from a mild and cheerful personality to very irritable, suspicious or inexplicably fearful. F. Poor comprehension, decreased ability to arrange things: can't keep up with other people's conversations, can't understand simple things, and don't prioritize things.  G. Familiar tasks cannot be completed, such as not being able to cook the previous specialty dishes, or doing things slower than before, often hesitating.  H. Loss of interest, no interest in everything, no desire, passive, unwilling to go out for a walk, and even need to be urged to eat.  I. Difficulty in expressing language, often forgetting some words, or unreasonably combining incoherent words, making it difficult for others to understand their speech.  J. Often puts things in the wrong place. |
| Neurologist 2:  Patients with Alzheimer's disease should pay close attention to sudden aggravation of mental symptoms (hallucinations, aggressive behavior, agitation) and adverse drug reaction symptoms such as severe bradycardia, muscle stiffness, etc., as well as eating in patients with advanced Alzheimer's disease. Post-choking cough, bed sore infection and other life-threatening situations that require medication adjustments require timely go to the hospital. |
| Neurologist 3:  When the patient has obvious psychotic symptoms, it refers to anxiety, depression and psychotic manifestations. Behavioral symptoms refer to aggression, apathy, agitation, disinhibition behavior, wandering, circadian disorder, and cursing and verbal aggressive behavior. It is easy to increase the care burden of patients, family members and caregivers, and affect the quality of life. |
| Neurologist 4:  (1) Memory decline, especially episodic memory impairment, can't remember the things I personally experienced and participated in:  For example, you forget to bring your medical insurance card every time you go out, or you make an appointment to go to the bank to withdraw money but forget it. Or I can't remember the more important things I did in the past week. Young people should also communicate more with their family members, talk more about what they said on the phone call last week, and see if their parents remember it? If there is a situation that you can't remember many times, you should go to the hospital to check it out.  (2) Decreased ability to plan work  For example, during the holidays in the past, you can get red envelopes and be able to do everything. This year, you need to ask your wife. The decline in the ability to handle complicated things and the loss of planning ability are also very important early manifestations.  (3) Difficulty in completing familiar tasks  For example, in the past, many dishes were prepared for the New Year's Eve dinner. This year, some dishes need to be prepared according to the recipes. This kind of complicated operation will cause certain problems.  (4) Can't tell the time or place: Can't remember the exact time or often get lost.  (5) Problems with language expression: Familiar words cannot be uttered in the process of speaking, and other words and sentences are used to explain, and it is difficult to find words.  (6) Frequent misplacing of things: for example, putting an electric kettle on a gas stove and similar situations.  (7) Impaired judgment: The elderly sometimes buy health care products, but some elderly people buy a lot of health care products.  (8) There is a problem with the sense of space: For example, when looking for a cup, the cup is right in front of your eyes but you just can’t see it, or you can’t react, it’s the cup.  (9) Withdrawal performance; unwilling to participate in social activities, do not want to do the previous job, uncertain and lack of confidence in one's own ability.  (10) Mood changes: For example, depression may be an early manifestation of Alzheimer's disease. |

| Question 11: How to help the families of late-stage Alzheimer's disease patients cope with the stress of daily living and caregiving? |
| --- |
| GPT3 Chinese version:  The care needs of patients with advanced Alzheimer's disease can be a daunting task that can place a lot of stress and burden on families. Here are some suggestions that may help:  Get professional help: Families can seek professional help, such as nurses, rehabilitation therapists, social workers, etc. These people can provide care advice, provide family members with tips and advice on caring for the patient, and also provide better care and medical treatment for the patient.  Taking care of yourself: Taking care of patients requires a lot of energy and physical strength, and family members also need to take care of themselves, including resting, sleeping and eating at the right time to maintain physical and mental health.  Create a schedule: Create a regular schedule that includes activities such as the patient's medications, meals, cleaning, sleep, and recreation. This helps family members organize their time so that they don't burn out.  Receiving Support: Family members can seek help from friends, church, community, or other support organizations. Sharing one's own emotions, stresses, and experiences can gain understanding and support from others.  Learn more: Families can learn more about Alzheimer's disease and learn how to better care for them. You can find books, online resources, courses, support groups, and more.  Seek support and help: When family members feel overwhelmed by stress, they can reach out for help and support. This may include bringing in other family members, friends, or professionals to help care for the person, or considering transferring the person to a hospital or nursing home.  Caring for people with advanced Alzheimer's disease is a difficult task, and family members need to take appropriate measures to maintain their own physical and mental health, and they also need to seek help and support from professionals. |
| GPT3 English version:  Caring for a loved one with late-stage Alzheimer's disease can be emotionally and physically challenging for families. Here are some suggestions that may help:  Seek Support: Joining a caregiver support group can help reduce feelings of isolation and provide emotional support. It can also be helpful to speak with a therapist or counselor who specializes in dementia care.  Plan and Organize: Developing a structured routine for daily activities can help reduce stress for both the caregiver and the patient. Keep a detailed schedule of medication times, appointments, and other important tasks.  Utilize Respite Care: Respite care is temporary care provided to the patient in a facility or by a professional caregiver, giving the primary caregiver a break. This can help reduce caregiver stress and prevent burnout.  Focus on Communication: As the disease progresses, communication can become increasingly difficult. Try using simple language and gestures to communicate with the patient. Engage in activities that the patient enjoys, such as listening to music or looking at old photographs.  Take Care of Yourself: Caregiving can be physically and emotionally exhausting. Take breaks when needed, prioritize self-care, and try to maintain a healthy work-life balance.  Remember, caring for a loved one with Alzheimer's disease can be overwhelming, but seeking support and utilizing resources can help alleviate some of the stress and improve quality of life for both the caregiver and the patient. |
| GPT4 Chinese version:  In the face of patients with advanced Alzheimer's disease, family members need to learn to deal with life and care pressure. Here are some suggestions:  Learn and understand: Increased knowledge about Alzheimer's disease, its progression, symptoms, and treatments will lead to better patient care and reduced anxiety.  Seek professional help: Consider consulting a professional doctor, nurse, or caregiver for advice and guidance on patient care. Community resources can also be sought, such as joining support groups and sharing experiences with other families.  Make a plan: Create a detailed care plan for the patient, including diet, sleep, activities, medical care, etc. Make sure other family members are aware of and involved in implementing the plan.  Shared Responsibility: Caring for someone with Alzheimer's disease is a daunting task that needs to be shared among family members. Allocate responsibilities and ensure that everyone is involved in the patient's care to reduce individual stress.  Taking care of yourself: Caregivers should pay attention to their physical and mental health and make sure they get enough rest and relaxation. Try exercising, meditating, or joining an interest group to cope with stress.  Maintain communication: maintain open communication with family members, face difficulties together, and provide mutual support. Sometimes it helps a lot to connect with someone who understands the challenges you face.  Seek psychological support: If you feel the stress is overwhelming, consider seeking counseling or psychotherapy. A professional counselor can help you cope with stress and learn to care for patients more effectively.  Consider placement options: Consider the need to place the patient in a skilled care facility, such as a nursing home or nursing home, as the condition progresses. While this is a difficult decision, it can sometimes be the best option to ensure patients receive proper care.  Finally, remember that caring for someone with advanced Alzheimer's disease requires patience and understanding. Seek help and connect with family, friends and support groups for support in times of stress and challenges. |
| GPT4 English version:  Coping with the stress of daily living and caregiving for families of late-stage Alzheimer's patients can be challenging. Here are some suggestions to help families manage this difficult situation:  Education: Learn about Alzheimer's disease, its progression, and the specific challenges faced by families and caregivers. This knowledge will help you anticipate and address the needs of the patient and provide better care.  Support groups: Join local or online support groups to connect with other caregivers in similar situations. Share experiences, coping strategies, and provide emotional support to one another.  Respite care: Arrange for temporary respite care, either through in-home care services, adult day care centers, or short-term residential facilities, to give caregivers a break from the daily responsibilities and reduce their stress.  Develop a routine: Establish a daily routine to manage the patient's needs, medications, and activities. This can help create a sense of predictability and reduce stress for both the caregiver and the patient.  Delegate tasks: Encourage family members and friends to take on specific tasks or responsibilities. This can help distribute the caregiving workload and provide some relief for the primary caregiver.  Prioritize self-care: Caregivers should prioritize their own physical and emotional well-being. Engage in activities that promote relaxation, such as exercise, meditation, or hobbies, and maintain a healthy diet and sleep schedule.  Seek professional help: Consider seeking professional assistance from a therapist or counselor specializing in caregiver stress or Alzheimer's-related issues. They can help provide guidance and coping strategies to manage stress and navigate the challenges of caregiving.  Financial planning: Consult with a financial planner or legal expert to plan for the future needs of the Alzheimer's patient and family. This can help alleviate financial stress and ensure proper care and support for the patient.  Communicate openly: Maintain open and honest communication with family members and friends about the challenges of caregiving. This can help create a supportive environment where everyone is aware of the difficulties and can contribute to finding solutions.  Practice patience and empathy: Be patient and empathetic with the Alzheimer's patient and remember that their actions are a result of the disease, not a personal choice. Be patient with yourself as well, as caregiving is a challenging and demanding role. |
| Neurologist 1:  ①Adjust your mentality, don't try to correct or change certain incorrect behaviors or views of the patient, accept the patient's current situation, and "admit" his views.  ② When the patient has mental symptoms, if there is no aggressive behavior and will not hurt himself or others, he can intentionally ignore it; if there is aggressive behavior, he should be separated from the person or thing that irritated him immediately.  ③ Seek help from other family members to share the workload of taking care of patients.  ④ Seek the help of a professional doctor. When the patient has aggravated symptoms or new symptoms, he should take the patient to the hospital in time to relieve the patient's symptoms through treatment and reduce the burden of care.  ⑤Maintain your own way of life, don't give up your life just because you are taking care of the patient wholeheartedly, and you can choose the way you like to adjust when you feel depressed during the process of caring for the patient. If necessary, mood-regulating drugs can be taken under the guidance of professional physicians. |
| Neurologist 2:  As the degree of aging continues to deepen, more and more people suffer from Alzheimer's disease. Most of the caregivers of Alzheimer's patients are their spouses or children and other immediate family members. They not only have to take care of the patients, but also perform other social and family responsibilities. They face long-term economic pressure and mental pressure, which makes them have great psychological burden. Current domestic and foreign studies have shown that the mental health of caregivers of Alzheimer's disease patients is significantly lower than that of non-Alzheimer's disease caregivers, and depression and anxiety are the main psychological problems in the family members of Alzheimer's disease patients. In recent years , scholars have found that the sources of psychological problems in the family members of patients with Alzheimer's disease are mainly three aspects: first, family members have insufficient understanding of Alzheimer's disease and lack of nursing knowledge and skills; The emergence of Alzheimer's disease patients in the family brings various burdens to family members, such as financial burden, family relationship and the physical health of family members themselves; the third is the degree of Alzheimer's disease patients, such as various mental and behavioral symptoms . Facing the above problems, how do caregivers of Alzheimer's disease deal with it?  Coping method 1. Accept the reality. At present, there is no cure for Alzheimer’s disease, and the most difficult thing for family members to do is to accept the reality. The only thing that can be done is to delay the progression of the disease, improve their symptoms, and treat Alzheimer’s disease with drugs. The suffering of the disease for patients and their families is minimized. Since we cannot change the reality, we can only face it calmly.  Coping method 2. Take care of yourself first. The caregiver must be fully prepared psychologically and physically. During the nursing process, arrange your own daily life reasonably, ensure a nutritious diet and good sleep. When the caregiver has symptoms of weakened immunity such as fatigue, headaches or common colds; lack of motivation in life, loss of interest; often feels helpless, frustrated, and powerless; unable to regulate and manage his emotions; attention Unable to concentrate; feelings of hopelessness in life, even suicide, etc., and cannot be adjusted by oneself, so it is necessary to seek professional help in time.  Coping method 3. Give yourself a chance to take a breath. When the caregiver encounters difficulties and faces pressure, don't deal with it alone. Learn to convert difficulties and resolve troubles through assistance. Harmonious family relationships help to have strong support and make caring meaningful. If conditions permit, family members can be arranged to take turns on duty. Make sure you have at least 1 hour of free time every day to relax yourself and relieve psychological pressure by going out for a walk, chatting, listening to music and other activities. If conditions permit, it is best to ensure that patients are not cared for one day a week and fully relax. If you are the only family member of the patient, in this case, you cannot have breathing time because there is no one to replace you, then may you consider hiring a nurse to take care of the patient alternately with you.  Response 4. Maintain a social circle Survey shows that the better a person’s interpersonal relationship is, the higher the happiness index is. Similarly, the more social activities a person has, the less likely it is to be depressed. Once you only face one person every day, you only need to do one thing repeatedly. When something happens, the feeling of depression will come to your face. Therefore, don't give up your social circle completely because you need to take care of patients. You can have time to chat with friends, and even a walk in nature will make you feel different.  Coping method 5. Keep exercising. In addition to continuing your social circle and trying to make yourself happy, you can also release your bad emotions through exercise. Studies have shown that the brain will produce a substance called endorphins after exercise. The quality of the mood is related to the amount of endorphins secreted by the brain. Exercise can stimulate the secretion of endorphins and increase the secretion of endorphins. Under the stimulation of endorphins, people's body and mind are in a relaxed and happy state. Endorphins are therefore also known as "happiness hormones" or "youth hormones", which can make people feel happy and satisfied, and can even help people relieve stress and unhappiness. Doing 30 minutes or more of aerobic exercise every day can gradually pull you back from a depressed state.  Coping method 6. Confidence and communication You can use the Internet to find groups similar to you. Everyone comforts and complains to each other. When a person finds someone who is in a similar situation to himself, he will have a sense of belonging. The time spent with someone who is in pain like me is also healing in itself.  Coping method 7. Recharging knowledge Some family members feel helpless because they lack the corresponding knowledge and skills to care for patients with Alzheimer's disease. The emergence of social networking on the Internet allows everyone to learn and share some nursing knowledge and skills in a timely manner, establish a correct concept of nursing under the guidance of doctors, and improve the quality of life of patients. On this basis, explore positive coping strategies, do what you can, and enhance the caregiver's sense of control and autonomy.  Coping method 8. Financial security money is a sensitive issue. If you confirm this kind of issue, you will avoid many conflicts. You should confirm the situation of financial support with the elderly and their families in advance. It is better for caregivers to record the family's financial expenditures and manage the expense bills well. As the saying goes, "brothers, clear accounts", it is necessary for family members to make a budget according to the needs of the elderly, whether using the elderly's own money or the money supported by other people should be budgeted as far as possible, and the source of payment for nursing expenses and daily living expenses for the elderly should be clarified.  Response 9. With the help of the social support system, a social insurance that brings benefits to the disabled and demented elderly - long-term care insurance (referred to as "long-term care insurance") is being piloted in some cities. Like the previous new rural cooperative medical care and comprehensive insurance for serious illnesses, insurance institutions play an important role in undertaking this social security insurance. Long-term care insurance: refers to the health insurance that provides compensation for nursing service expenses to the insured who needs long-term care due to old age, illness, or disability. The long-term care insurance for employees focuses on the daily care of the insured who has been disabled or semi-disabled for a long time, and the medical care and other services that are closely related to basic life. As a basic social insurance system, the long-term care insurance system mainly covers the insured persons of the employee basic medical insurance (hereinafter referred to as employee medical insurance). You can consult your local social security office.  If the symptoms of depression and anxiety have affected your health, it is recommended that you go to a tertiary hospital to take a depression self-examination scale to confirm your current situation. Once you are diagnosed with depression, you must take medicine and cooperate with psychology. treat. |
| Neurologist 3:  Caregiver psychological intervention: 1. Caregiver education 2. Teaching problem-solving skills 3. Access to resources 4. Long-term planning 5. Emotional support 6. Short-term breaks. |
| Neurologist 4:  In the advanced stage of Alzheimer's disease, the disease can seriously affect various systems of the body, such as motor coordination, bowel, bladder function, and even respiratory function.  (1) Diet care  One of the most important daily care tasks in advanced Alzheimer's disease is dietary care. Serve high-calorie foods. In this way, under the same nutrient intake, the pressure of taking care of eating can be reduced. When patients experience weight loss, energy supplementation should be ensured. Chewing, swallowing, and digestion become a challenge. Patients may suddenly lose focus during eating and forget that they are eating. Caregivers usually need to accompany the whole process of eating, and prompt chewing and swallowing through gestures, mouth shapes and simple words. In order to maintain the burden on the digestive system, eat as few frequent meals as possible. It is necessary to allow enough time for the patient to eat, and maintain a comfortable, upright posture for 30 minutes after meals to help digestion. Choose liquid food, oatmeal, lotus root powder, fruit juice, milk and soup can all be used to supplement daily nutritional needs. In order to reduce the patient's choking and coughing, thickeners can be used appropriately. At the same time, to encourage patients to eat by themselves, family members can try to provide "cues" for eating, such as gently holding the patient's hand on the spoon containing food, and then extending the spoon to the mouth.  (2) smooth defecation  During this stage, patients are prone to toileting difficulties and may need guidance in walking to the bathroom. Track the regularity of defecation, record when patients usually go to the bathroom every day, or when they are prone to incontinence, and regularly remind them to help patients defecate. You can consider using a mobile toilet to reduce some of the burden of care. Control fluid intake. Reduce fluid intake at least two hours before going to bed to avoid nighttime defecation needs or urinary incontinence; daily water intake should still be adequate, including the intake of liquid foods such as milk, juice, and soup, with a total of 6-8 (about 1·5-2·0 liters) cup is appropriate. Use absorbent and protective products. Use adult diapers and adult nursing mattresses as nighttime aids to reduce caregiving stress. Stomach conditioning. When the patient's bowel habits change, dietary adjustments are required. For example, patients with constipation can increase fiber-rich foods such as whole grains, bamboo shoots, etc. in their diet.  (3) Prevention of pressure sores  People with advanced Alzheimer's disease may be bedridden or seated for long periods of time, making them prone to pressure sores (decubitus sores). Pressure sores are often caused by long-term pressure on local tissues, and may lead to systemic infection and death in severe cases. The following points should be done to prevent pressure sores : ①Turn over frequently: once every 2 hours ; ②Use air mattresses and decompression stickers; ③Avoid skin damage due to friction: raise the head of the bed <45°, and keep the bed surface flat , Do not drag when turning the patient; ④Keep the local skin clean and dry;  (4) Avoid infection  Patients with advanced Alzheimer's disease have relatively low immunity and are prone to infection. Keep your mouth clean. Good oral hygiene can reduce the risk of pneumonia from bacteria in the mouth. Clean with a soft toothbrush or damp gauze pad, if the patient wears dentures, they need to be cleaned after use. Treat wound immediately. When trauma is caused, it should be treated immediately, clean the wound with normal saline or water, and apply iodophor solution 3-4 times a day to prevent infection. For deep wounds or suppurative situations, it is recommended to go to the hospital for treatment in time. Prevent flu and pneumonia. For many diseases that lead to prolonged bed rest, pulmonary complications are one of the major risks of death. The living environment of patients should not be isolated from the outside world, and it is difficult to ensure that the source of infection and the route of transmission are cut off. Vaccines can be given regularly to prevent the occurrence of these diseases. In addition, you should pay attention to the temperature of the room, put on and take off your clothes in time, and keep warm after wiping; avoid cross-infection during the flu season, and if caregivers have a cold, try to avoid contact with the bedridden elderly with Alzheimer's disease.  (5) Identify pain  Pain often means that there are some problems in the body, and it is difficult for patients with advanced Alzheimer's disease to express pain and discomfort in a timely or correct manner. A few characteristics can help caregivers recognize and seek prompt treatment or medical attention: Watch for signs. Watch for signs of pain and illness, such as pallor or redness, swelling in body parts, tremors, vomiting, etc. Pay attention to nonverbal signals. Gestures, vocalizations, and facial expressions may indicate discomfort. Changes in mental behavior. Anxiety, agitation, yelling, sleep problems, etc. Although these may be caused by Alzheimer's disease, sudden changes in mental behavior are likely to be signs of illness or pain. Caring for someone with advanced Alzheimer's disease can be a daunting task, and end-stage care options may include hiring professional caregivers, choosing a long-term care facility, and end-of-life care. |

| Question 12: How can Alzheimer's disease patients receive psychological and emotional support? |
| --- |
| GPT3 Chinese version:  Alzheimer's disease is a chronic neurological disorder that can negatively impact a patient's mental and emotional well-being. Here are some suggestions for helping patients find psychological and emotional support:  Seeking professional help: Seeking the help of a professional psychologist or psychological counselor can provide patients with effective  emotional and psychological support to help them deal with the problems and challenges associated with Alzheimer's disease.  Join a support group: Joining an Alzheimer's support group can help people and their families and caregivers share experiences and emotional support, as well as access information about Alzheimer's treatment and care.  Stay socially connected: Patients need to stay socially connected as much as possible, staying in touch with family, friends, and others in the community to reduce feelings of loneliness and isolation.  Find mental health resources: There are organizations and agencies that provide mental health services and resources for people with Alzheimer's disease and their families. These resources may include counseling, support groups and other related services.  Maintaining a positive attitude towards life: Patients need to try to maintain a positive attitude towards life and find ways to maintain physical and mental health. This may include playing sports, traveling, reading, listening to music, and more.  Receiving caregiver support: Caregivers and family members may need to receive psychological and emotional support to help them cope with the condition and challenges of a person with Alzheimer's disease. They can seek professional support, join a support group, or find other forms of support and resources. |
| GPT3 English version:  Alzheimer's disease is a degenerative brain disorder that affects memory and cognitive function, which can be emotionally and psychologically challenging for both patients and their families. Here are some ways Alzheimer's disease patients can receive psychological and emotional support:  Cognitive Stimulation Therapy: This type of therapy involves participating in activities that help stimulate the brain, such as puzzles, reading, and games.  Music Therapy: Music can have a positive impact on mood and can help stimulate memories. Listening to music or participating in singing or instrument playing can be beneficial.  Art Therapy: Art therapy can help with cognitive function, communication, and self-expression. Painting, drawing, and other forms of artistic expression can be beneficial.  Support Groups: Joining a support group with other individuals who are going through similar experiences can provide emotional and psychological support. These groups can be facilitated by mental health professionals, social workers, or other trained individuals.  Counseling: Seeking counseling from a mental health professional can provide a safe space for individuals to discuss their feelings, fears, and challenges related to Alzheimer's disease.  Caregiver Support: Caregivers of Alzheimer's disease patients also need support. They can attend support groups, receive counseling, and participate in respite care programs that provide temporary relief from caregiving responsibilities.  It is essential to remember that each individual with Alzheimer's disease is unique and may require different types of support. It is important to work with healthcare professionals, such as geriatric psychiatrists, to determine the best course of action for each individual's needs. |
| GPT4 Chinese version:  Alzheimer's disease is a neurodegenerative disease that gradually affects memory, thinking and behavior. Providing psychological and emotional support to people with Alzheimer's is important to help them cope with the challenges the disease presents. Here are some suggestions:  Seek professional support: Find a professional psychologist or psychotherapist who can provide psychotherapy or counseling for people with Alzheimer's disease.  Support from relatives and friends: let family and friends understand the condition, encourage them to spend more time with the patient, listen to their feelings, and provide care and support.  Join a support group: Many areas have support groups for people with Alzheimer's and their families. Here, patients and families can share experiences, exchange information and receive emotional support.  Community Resources: Contact your local Alzheimer's Association or related organization to learn about the resources and services they offer. These organizations often provide a variety of educational, psychological and emotional support for patients and families.  Develop good living habits: Maintaining a regular schedule, diet and exercise is very important for improving mental health and reducing symptoms such as depression and anxiety.  Increase social activities: Participating in some social activities suitable for patients, such as participating in interest groups, classrooms, etc., can help patients maintain connections with others and reduce loneliness.  Learn to cope with stress: Learning some ways to cope with stress, such as deep breathing, progressive muscle relaxation or meditation, can help reduce the patient's tension and anxiety.  Maintain an optimistic attitude: Encourage patients to maintain a positive attitude and focus on the abilities and strengths they still have, rather than focusing only on what they have lost.  Patience and understanding: Caring for someone with Alzheimer's requires a lot of patience and understanding. Family members and friends should learn to adapt to changes in the patient's condition and try to meet their needs. Safe and comfortable environment: Create a safe, comfortable and easy-to-use living environment for patients. Make sure there is adequate lighting in the home to reduce the risk of falls, and use labels and pictures to help patients identify objects and spaces.  Encourage autonomy: Encourage patients to complete daily tasks, such as dressing and eating, as autonomously as possible within the scope of their condition. This helps maintain their self-esteem and confidence.  Maintain Communication: Maintain open, honest, and loving communication with patients. Try to communicate with the patient in simple, unambiguous language, avoiding complex sentences or words.  Using music and art therapy: Music, drawing, and other forms of art therapy can help patients express emotions and reduce anxiety and depression.  Increase cognitive activities: Some moderate cognitive activities, such as puzzles, reading, playing poker, etc., can help patients keep their brains active and slow down the progression of the disease.  Self-care for caregivers: Family members and friends caring for someone with Alzheimer's disease also need to pay attention to their own physical and mental health. Make sure you have enough time for rest and relaxation, seek professional help or join a support group to deal with caregiver stress and challenges. |
| GPT4 English version:  Alzheimer's disease patients and their caregivers can face many psychological and emotional challenges. It's essential to provide support to maintain the well-being of both the patients and their families. Here are some ways to offer psychological and emotional support to individuals with Alzheimer's disease:  Professional support: Seek guidance from healthcare professionals such as neurologists, psychiatrists, psychologists, or geriatricians who specialize in Alzheimer's disease. They can provide appropriate assessments, treatments, and advice for managing the emotional and psychological symptoms.  Support groups: Join local or online support groups specifically designed for Alzheimer's patients and their caregivers. These groups can provide a platform for sharing experiences, learning coping strategies, and fostering a sense of community and understanding.  Counseling and therapy: Individual or family therapy sessions with a mental health professional can help patients and their families cope with the emotional challenges of Alzheimer's disease. Cognitive-behavioral therapy, reminiscence therapy, and validation therapy are some examples of therapeutic interventions that can be beneficial.  Educate yourself and your family: Understand the disease, its progression, and available treatments. Being well-informed can help in managing expectations and adapting to the challenges that come with Alzheimer's.  Maintain social connections: Encourage Alzheimer's patients to engage in social activities and maintain relationships with friends and family. Social interaction can provide emotional support and enhance the patient's quality of life.  Encourage self-expression: Art, music, and other forms of creative expression can help Alzheimer's patients to communicate their emotions and maintain a sense of identity.  Establish a daily routine: Having a predictable daily schedule can provide a sense of stability and comfort for Alzheimer's patients, reducing anxiety and agitation.  Ensure a safe and supportive environment: Create a calm, clutter-free, and safe living space for the patient. This can minimize confusion and stress while promoting a sense of security and independence.  Practice self-care for caregivers: Caregivers should take care of their own physical and mental health to be better equipped to support Alzheimer's patients. Engage in regular exercise, eat well, maintain social connections, and seek respite care when needed.  Reach out for help: Recognize when additional help is needed, and don't hesitate to seek assistance from professionals, family members, or friends. This can help alleviate caregiver stress and ensure that the patient receives the care they need.  Remember that every person with Alzheimer's disease is unique, and their needs may change over time. Remain flexible and adapt support strategies as necessary. |
| Neurologist 1:  1. Don't belittle or belittle patients, but encourage and agree with them instead. 2. Don't easily argue with patients, but understand and agree with them as much as possible. 3. Don't be ashamed of patients behaving inappropriately in public, try to guide patients to do other things or distract them. 4. Caregivers themselves should do some self-psychological counseling work on a regular basis. At the same time, you can also seek the help of professional nurses to reduce your own burden. 5. Arrange an exercise schedule for patients, such as helping them relax their joints, rubbing their hands, walking with them, etc. 6. Accumulate knowledge about Alzheimer's disease, including the mechanism of disease, disease development process, disease behavior, drug intervention methods, and precautions for disease care, which will not only help patients get better care, but also It also reduces the burden on caregivers. 7. Often show the patient old photos and help them recall the past time, and play the music that the patient liked to listen to in order to arouse the patient's memory of the old time. |
| Neurologist 2:  Only by fully respecting and caring for these patients can they have a better quality of life, reduce the difficulty of care, and make the care journey smoother. Caregiving attention Everyone wants to be treated with respect, and people with dementia are no exception. In the care of the elderly with Alzheimer's disease, we must give due respect, maintain their dignity, and try to meet their reasonable requirements; we must not use words or actions that hurt feelings or damage the self-esteem of the elderly, causing them psychological harm. Depressed mood, even aggressive behavior; don't force the elders to do things they don't want, and speak more caring words to the elders; make them feel needed, cared for, respected, and increase their sense of belonging.  In addition to memory loss due to Alzheimer's disease, some old people may also often fight and throw objects, act coquettishly, and lose their temper with their children. In the eyes of children, it is a manifestation of disobedience, and sometimes it is an emotion created by parents who are very scared and uneasy in order to attract children's attention. Behind the seemingly inexplicable problematic behaviors of many elderly people with dementia, it is often the expression of their emotions and emotions. Because of the pressure of caring, family members and caregivers often ignore these emotional needs, and the squeezed emotional needs further stimulate the elderly. emotions, making them prone to problem behaviors. Caregiver attention Alzheimer's disease patients often have difficulty understanding, but they are very sensitive to the tone of speech. An excited tone will make the patient feel uneasy, while a calm tone can make them feel at ease. Therefore , try to keep calm when speaking as much as possible, and pay attention to the following aspects: ① Avoid quarrels ② Eliminate doubts ③ Properly handle ④ Understand mood swings ⑤ Seek support and help ⑥ Change nursing methods. When the patient is emotional, it is best for the caregiver to change the subject as soon as possible, so that they can quickly forget the reason for their anger.  Elderly people with Alzheimer's disease often feel depressed and stressed, as well as angry at sudden events, such as things falling on the floor, and sometimes the mounting frustrations of everyday life can bring them to a breaking point of anger. What we think of as simple tasks is actually quite complicated for them because it is difficult to remember every step and sequence. Elderly people with dementia often have difficulty with these small steps, which can easily lead to frustration and subsequent outbursts of anger. Their world has become increasingly chaotic and unruly, so it is easy to feel "too much" and react with anger. So to reduce the outbursts of "dementia rage", it is important to reduce their sense of stress. Care attention ① caregivers should not force them to work hard to do something, so as not to be pushed to the limit. Understand that their brains are failing and they are losing basic knowledge and abilities. So just accept their current situation and let them do what they can. ② To reduce complex choices, it is not necessary to help them decide their daily schedule, because they still need to make some decisions of their own; but it is not necessary to let them decide all of them. Our goal is to simplify the options to make it easier for them to choose. ③ Speak clearly and clearly. Dementia will affect the brain's ability to process and retrieve information. It is easier to understand with short and direct sentences, and each sentence has only one instruction. The purpose of this is to make them think less and remember less. If you're giving instructions, do it step by step; if you're expressing things, keep a single line of thought. Also, a warm and positive tone will be more effective. |
| Neurologist 3:  1) Early stage: the patient's symptoms are mild, and a few people may have a certain degree of insight. At this time, the nature, treatment and prognosis of the disease should be told to the patient to help the patient further understand his condition. With the help of family members, give some simple guidance on improving memory and life ability training methods, let patients participate in housework, and at the same time tell patients to give up doing things that require nervous brain and are prone to danger, such as driving a car, swimming, etc.  2) Mid-term: The symptoms are more serious, and the insight is lost, and the memory and living ability are obviously decreased. At this time, the family environment should be kept as calm and comfortable as possible, and the furniture should be simple and convenient, and should not be changed frequently. Develop a daily routine and carry out occupational therapy. In addition to participating in simple housework, patients can also be allowed to do some work (such as recreational activities) that they are interested in and within their ability. In order to improve the patient's memory, nostalgia therapy and music therapy can be carried out. Use the past memory stored by the patient to recall and strengthen it. For example, showing the patient repeatedly the meaningful photos of the past (wedding photos, family portraits, etc.), and telling the unforgettable memories of the past, can improve the patient's mood, calm agitated behavior, and improve the remaining memory function. In addition, repeatedly giving orientation and memory reinforcement (such as repeatedly emphasizing time, space and character training), chatting with patients about the books, newspapers and magazines they are interested in, and letting patients participate in simple intellectual games (such as simple jigsaw puzzles) are all helpful. Helps improve memory.  3) Late stage: At this time, most patients cannot take care of themselves, and most of their memory is lost. In addition to providing daily care and physical function training for the patients, family members should have more contact with the patients, have a good attitude, and try to meet their requirements to prevent them from developing " Abandoned" thoughts. In addition, mental symptoms such as emotional depression, hallucinations, delusions, and agitation often appear during the course of the disease, which brings certain harm to patients and the society, and also brings certain difficulties to the care of family members. It is also the main reason for the diagnosis, treatment and hospitalization of this disease reason, we must pay attention to it. Therefore, patients should be observed carefully and regularly, and the evolution of the condition should be paid attention to. For patients with serious negative behaviors, hurting others, violent behaviors, and obvious hallucinations, delusions and other dangerous behaviors, if it is difficult to take care of them, they should be hospitalized in time. For advanced patients who cannot take care of themselves, they should be advised to live in nursing homes for the elderly. The roamer should be restricted or accompanied to go out, and the identification card with the patient's name, address and other content can also be placed on the patient so that when he is lost, others can escort him home. At the same time, family members should also learn to master some safe nursing knowledge about mental symptoms, and strengthen the awareness of preventing dangerous behaviors such as hurting others, roaming, and suicide, so as to prevent accidents. |
| Neurologist 4:  (1) Treat patiently: family members and medical staff should care for and love the patient, pay attention to respect the personality of the patient, be kind and pleasant in conversation, and avoid using words such as stupid and stupid. At the same time, according to the psychological characteristics of different patients, methods such as encouragement, comfort, and hints should be used to give enlightenment. For patients with pessimistic mood, patiently explain and introduce some typical cured cases to arouse the patients' confidence and courage to overcome the disease. Family members should take the initiative to take care of patients with difficulties in life, take care of them enthusiastically, and warm their hearts with practical actions.  (2) Do a good job of psychological counseling: At the early stage of the elderly suffering from Alzheimer's disease, the psychological changes are complex and the emotions are changeable. Confidence in life, at this time, we must do a good job in psychological counseling for patients, eliminate their worries in time, communicate with them in the heart at ordinary times, encourage patients to tell their distress in their hearts, and come to counsel them.  (3) Recalling the past: This is the best way to encourage the elderly to keep thinking. But try to avoid recalling sad events. The elderly have a sense of accomplishment, so let them tell more interesting stories about "passing five trials and beheading six generals", so as to activate their thinking and drive away sadness and depression with optimism. If you let him live alone and feel depressed all day long, it will only accelerate the development of Alzheimer's disease.  (4) Integration into society: Family members can help patients to broaden their thinking, keep up with the changes of the times, and let the brain receive fresh stimulation, so as to prevent the brain from shrinking. Take the elderly for a walk and see the outside world. Only in this way will their thinking become more active, their emotions will be more optimistic, and their mood will be more comfortable. Patients should be encouraged to participate in some learning and social and family activities within their capabilities to distract patients from their attention and negative emotions and arouse their confidence in life. |

| Question 13: How to determine the stage of Alzheimer's disease progression in a patient? |
| --- |
| GPT3 Chinese version:  Alzheimer's disease is a progressive degenerative disease, usually divided into early, middle and late stages. The following are common diagnostic methods and features that can help determine where a patient is currently in the disease process:  Early stages: Mild memory problems, such as not remembering recent events or recalling earlier experiences Impact on abstract thinking and planning skills Gradual social and language impairment Changes in behavior and personality Often feeling confused, tired, or depressed  Middle stage: memory worsens, may forget important personal information, such as home address, phone number, etc. Speech skills are further impaired, difficulty organizing speech or understanding what others say Emotions and behavior become more unpredictable, may show anger, critical or resist possible hallucinations or delusions  Late stages: Severely impaired memory and speech, difficulty expressing or understanding speech Impaired body movement and balance, needing help to walk or perform daily activities May not be able to control bowel movements, mental instability, often hallucinations and delusions.  As the disease progresses, the patient's cognitive and behavioral functions will be increasingly affected. Physicians can determine what stage of Alzheimer's disease a patient is currently in by conducting cognitive and behavioral assessments, as well as performing brain imaging tests. |
| GPT3 English version:  The stage of Alzheimer's disease progression can be determined using a variety of methods, including clinical assessment, cognitive testing, and neuroimaging. Here are some common approaches:  Clinical assessment: A clinician can evaluate the patient's symptoms and medical history to determine the stage of Alzheimer's disease. The clinician may use standardized tests such as the Mini-Mental State Examination (MMSE) or the Clinical Dementia Rating (CDR) scale to assess cognitive function.  Cognitive testing: Neuropsychological testing can provide a detailed assessment of cognitive function, including memory, attention, and language abilities. These tests can help determine the severity of cognitive impairment and provide a baseline for monitoring disease progression over time.  Neuroimaging: Brain imaging techniques such as magnetic resonance imaging (MRI) or positron emission tomography (PET) can detect structural changes in the brain associated with Alzheimer's disease. These changes include the buildup of amyloid plaques and tau protein tangles, which are hallmark features of the disease.  Biomarkers: Researchers are developing biomarkers, such as blood tests or cerebrospinal fluid (CSF) tests, that can detect early signs of Alzheimer's disease before symptoms appear. These tests may also be useful for monitoring disease progression and response to treatment.  It is important to note that Alzheimer's disease progression can vary widely between individuals, and the staging system used may differ depending on the healthcare provider or organization. |
| GPT4 Chinese version:  Alzheimer's disease is a common neurodegenerative disease, the course of which is usually divided into three stages: early, middle and late. Determining which stage a patient is in requires an assessment of symptoms and cognitive abilities. Please note that it may not be accurate to judge based on symptoms alone, and the diagnosis and course of disease should be judged by professional doctors.  Early stage (mild Alzheimer's disease) The symptoms of early Alzheimer's disease may be relatively mild and undetectable. The main manifestations are:  memory impairment, especially impairment of recent memory  mood swings and anxiety  Gradual decline in social and occupational competence  Difficulty handling complex tasks and plans  In the middle stage (moderate Alzheimer's disease) , symptoms of middle stage Alzheimer's disease become more pronounced and may include:  Further deterioration of memory, including impairment of long-term memory  Decreased language skills, difficulty organizing sentences and finding the right words  Cognitive decline, making it difficult to make decisions and solve problems  behavioral and emotional problems, such as irritability, depression, anxiety, and insomnia  Impaired spatial and visual cognition  Advanced (Severe Alzheimer's Disease) - People with advanced Alzheimer's disease have very severe symptoms and often require round-the-clock care. Key symptoms include:  Severe memory impairment, who may not be able to recognize relatives and friends;  Loss of activities of daily living, such as eating, dressing and washing;  Speech is severely impaired and may not be able to communicate at all  Behavioral problems such as hallucinations, delusions, and aggressive behavior in most patients  Muscle stiffness, resulting in limited mobility and possible loss of walking  Note that these symptoms may vary from individual to individual. If you suspect that you or a family member may have Alzheimer's disease, seek immediate professional evaluation from your doctor. Doctors use the history, physical exam, cognitive assessment, and other tests to determine the stage of the disease. |
| GPT4 English version:  I am not a doctor, but I can provide you with some general information on how to determine the stage of Alzheimer's disease progression in a patient. It is essential to consult a healthcare professional for a thorough assessment and accurate diagnosis.  Typically, Alzheimer's disease is classified into three main stages: mild (early stage), moderate (middle stage), and severe (late stage). A medical professional can help determine the stage based on a combination of factors:  Patient history and interview: The doctor will gather information about the patient's symptoms, medical history, and any changes in memory, thinking, and behavior.  Cognitive tests: A variety of cognitive tests, such as the Mini-Mental State Examination (MMSE) and the Montreal Cognitive Assessment (MoCA), can be administered to assess cognitive function, memory, attention, language, and other skills.  Neuropsychological testing: Comprehensive testing that evaluates various cognitive domains can help determine the extent of cognitive impairment and identify specific deficits.  Brain imaging: CT scans, MRI, or PET scans can be used to detect changes in the brain's structure and function. These imaging techniques can help rule out other potential causes of cognitive decline, such as tumors or strokes, and may provide additional information about the stage of Alzheimer's disease.  Blood tests and biomarkers: Blood tests can help rule out other potential causes of cognitive impairment, and some emerging biomarker tests may help determine the stage of Alzheimer's disease.  Activities of daily living (ADL) assessment: A healthcare professional will evaluate the patient's ability to perform essential daily tasks independently, such as dressing, bathing, and eating. The level of assistance required can help determine the stage of Alzheimer's.  The stages of Alzheimer's disease generally include:  Mild (early-stage): Patients in this stage may have difficulty remembering recent events, misplacing items, and struggling with problem-solving or complex tasks. They may still be able to perform daily activities independently but may require assistance with more complicated tasks.  Moderate (middle-stage): In this stage, patients may experience more significant memory loss, difficulty recognizing friends and family, and increased confusion. They may also have difficulty with language, motor function, and performing daily activities without assistance.  Severe (late-stage): In the severe stage, patients often lose the ability to communicate, require full-time assistance with daily activities, and may have difficulty walking or sitting up. They may also experience significant personality and behavioral changes.  Remember that only a healthcare professional can accurately diagnose and determine the stage of Alzheimer's disease in a patient. If you suspect that you or a loved one may have Alzheimer's, consult a medical professional for an evaluation. |
| Neurologist 1:  Stage 1: No impairment; Stage 2: Very mild cognitive decline; Stage 3: Mild cognitive decline; Stage 4: Moderate cognitive decline; Stage 5: Severe cognitive decline; stage 6: severe cognitive decline; stage 7: very severe cognitive decline. |
| Neurologist 2:  Mild Alzheimer's disease (early stage): Patients at this stage have the ability to live independently, but there will be some difficulties: forgetting names and words: they can't call names, and can't find the correct words.  Mild to moderate Alzheimer's disease (early to mid-stage): People at this stage experience the following difficulties: Task difficulty: Difficulty completing social or work tasks. Dyslexia: After reading an article, having difficulty remembering what it contains. Misplacement: Losing or misplacing valuables. Loss of Execution: Decreased ability to plan or organize.  Moderate Alzheimer's disease (intermediate stage): Moderate Alzheimer's disease is the longest-lasting stage, usually lasting many years. At this stage, the patient's symptoms are more obvious, mainly including: loss of remote memory: forgetting things experienced in the past, and impairment of episodic memory. Mood changes: irritability is a prominent feature. Time and space confusion: not sure where they are and the exact date. easy to get lost. Personality changes: Suspicious, paranoid, and obsessive-compulsive, repeating certain actions, such as rubbing hands or tearing toilet paper. Sleep disturbance: sleeping during the day, restless at night ("sunset syndrome"). Hallucinations (taking yourself in the mirror as a stranger); hallucinations (seeing deceased relatives).  Severe Alzheimer's disease (late stage): People at this stage: Unaware of their surroundings. Unable to respond, unable to have a conversation (only words). Unable to control actions. Needing help with meals or toileting. Lost smile, couldn't sit down by himself, couldn't look up. Reflexes become abnormal. Muscles become stiff. Decreased swallowing ability. Susceptibility to disease, especially pneumonia. |
| Neurologist 3:  The first stage (1 to 3 years)  For mild dementia. Manifested as memory loss, prominent forgetting of recent events; decline in judgment ability, patients cannot analyze, think, and judge events, and find it difficult to deal with complex problems; work or housework is inattentive, unable to independently carry out shopping, economic affairs, etc., social difficulties; Although still able to do some familiar daily work, he is at a loss and incomprehensible to new things, emotionally indifferent, occasionally agitated, and often suspicious; time disorientation occurs, and he can orient himself to the place and person he is in. , Difficulty in orienting to the geographical location, poor visuospatial ability of complex structures ; less verbal vocabulary, difficulty in naming.  The second stage (2 to 10 years)  For moderate dementia. It is manifested as severe damage to near and far memory, decreased visuospatial ability of simple structures, and time and place orientation disorders; severe damage in dealing with problems and identifying similarities and differences in things; inability to independently carry out outdoor activities, in dressing , personal Need help in hygiene and maintaining personal appearance; inability to calculate; various neurological symptoms, aphasia, apraxia and agnosia can be seen; emotion changes from apathy to restlessness, often walking around, and urinary incontinence can be seen.  The third stage (8 to 12 years)  For severe dementia. The patient has been completely dependent on the caregiver, has severe memory loss, and only fragments of memory remain; he cannot take care of himself in daily life, he is incontinent, presents silent, stiff limbs, positive pyramidal tract signs can be seen on physical examination, and there are primitive symptoms such as strong grasping, groping and sucking. reflection. Eventually coma, usually died of complications such as infection. |
| Neurologist 4:  (1) Stage 1: Appearing normal behavior: At this early stage, he will not have any symptoms to detect. Only Aβ and Tau tests (PET scan, cerebrospinal fluid or blood) can reveal whether he has Alzheimer's disease. As the disease progresses, his thinking and reasoning will change more and more noticeably.  (2) Stage 2: Very slight changes You may still not notice anything wrong with his behavior, not even doctors, but he himself may notice small differences like forgetting words or not remembering Where to put things. Mild symptoms at this stage will not affect his ability to work or live independently. Keep in mind that these symptoms may not be signs of Alzheimer's disease at all, but just normal changes with aging.  (3) The third stage: mild decline stage, family members may begin to notice changes in the patient's thinking and reasoning, for example: forgetting what he just read; asking the same questions over and over again; making plans or organizing activities harder and harder; can't remember new friends' names, etc.  (4) Stage IV: Moderately declining stage, the patient's thinking and reasoning problems become more pronounced, and new problems arise: for example, difficulty writing the correct date and amount on a check; inability to remember the month and season; difficulties cooking and even ordering from the menu.  (5) Stage V: Moderate to Severe Declining Stage: Patients may begin to lose track of where they are and what time it is. It may be difficult to remember his address, phone number or where he used to go to school. May be confused about what to wear on a given day or in different seasons.  (6) Stage 6: Severely declining stage: As Alzheimer's disease progresses, he may remember faces but not names; may mistake one person for another. For example: think that the wife is the mother. Occasionally hallucinating, even though he has been away from work for a long time, he will repeatedly think that he needs to go to work.  (7) The seventh stage: a very serious decline stage: many of his basic skills, such as eating, walking, and sitting still, will degenerate or even completely lose at this stage. May be bedridden all day, completely unable to communicate. |

| Question 14: How to determine the stage of Alzheimer's disease progression in a patient? |
| --- |
| GPT3 Chinese version:  Alzheimer's disease is a progressive neurodegenerative disease with severe cognitive and behavioral symptoms in advanced stages. Here are some serious symptoms that people with advanced Alzheimer's disease may experience and suggestions for how to manage and relieve them:  Loss of speech: People with advanced Alzheimer's disease may lose speech, making it difficult to communicate with family members and caregivers. To alleviate this, families can use pictures and gestures to convey information, and try activities such as listening to music and watching movies to help the patient relax and feel comfortable.  Loss of movement ability: People with advanced Alzheimer's disease may lose the ability to move and become dependent on others for basic life care. Family members can provide them with a comfortable sleeping environment and help them adjust their posture. Diet also requires special attention, providing nutritious and easy-to-digest foods as much as possible.  Loss of self-awareness: People with advanced Alzheimer's disease may lose the ability to recognize themselves or family members. Family members can help patients build a sense of security and trust, provide as much familiar environment and companionship as possible, and try to show photos and evoke some memories.  Anxiety and depression: People with advanced Alzheimer's disease may experience anxiety and depression. Families can use gentle, quiet words and touch to reassure them, offer close hugs and warm hugs, or try deep breathing or meditation to ease emotions.  Loss of independence: People with advanced Alzheimer's disease may become completely incapacitated and require round-the-clock care. Families may consider hiring professional nursing staff or seeking other nursing resources to help care for the patient and work with their physicians to develop a comprehensive plan of care.  For patients with advanced Alzheimer's disease and their family members, the most important thing is to establish a safe, warm and comfortable environment, provide warm companionship and care, and relieve the patient's symptoms and discomfort as much as possible, while seeking professional medical treatment. With the support and assistance of nursing resources, a complete nursing plan is established to ensure that the patient receives the best attention and care. In addition, family members also need to pay attention to their own health and mental health, and seek support and help as much as possible to cope with the challenges and pressures of caring for patients. |
| GPT3 English version:  In the late stages of Alzheimer's disease, the symptoms can become quite severe, leading to a significant decline in cognitive, physical, and emotional functioning. Some of the potential symptoms that may manifest in the late stages of Alzheimer's disease include:  Severe memory loss: The individual may not be able to remember their own name or recognize loved ones.  Difficulty with communication: The individual may have trouble communicating verbally and may resort to nonverbal cues, such as facial expressions or hand gestures.  Loss of mobility: The individual may have difficulty walking, standing, or even sitting up.  Incontinence: The individual may have difficulty controlling their bladder or bowels.  Increased agitation and aggression: The individual may become more agitated and aggressive, particularly in situations where they feel overwhelmed or confused.  Sleep disturbances: The individual may have trouble sleeping at night and may become increasingly disoriented.  To manage and alleviate these symptoms, family members can take a few strategies, including:  Creating a calm and familiar environment: Keeping the individual in familiar surroundings with familiar people, objects, and routines can help reduce confusion and agitation.  Providing comfort and reassurance: Offering comfort and reassurance can help alleviate anxiety and promote a sense of safety.  Encouraging engagement in activities: Engaging the individual in activities that they enjoy, such as music, art, or exercise, can help reduce agitation and promote a sense of purpose.  Using medication and therapies: In some cases, medications or therapies may be prescribed to manage symptoms such as agitation or sleep disturbances.  Seeking support: Caregivers may benefit from support groups or counseling to help them cope with the challenges of caring for a loved one with Alzheimer's disease.  Overall, managing late-stage Alzheimer's disease can be challenging, but with the right strategies, caregivers can help alleviate symptoms and improve quality of life for their loved one. |
| GPT4 Chinese version:  Alzheimer's disease is a common neurodegenerative disorder usually manifested by progressively worsening cognitive and behavioral symptoms. In advanced stages of the disease, patients may experience severe symptoms such as:  Severe memory impairment: People with advanced Alzheimer's disease may not recognize loved ones and friends, may not understand where they are, and may even forget how to perform basic life activities.  Language and communication difficulties: Patients may lose the ability to speak and understand language, making it difficult to communicate effectively.  Behavioral problems: Patients may experience psychological symptoms such as anxiety, depression, and irritability, as well as behavioral problems such as wandering and increased nocturnal activities.  Unable to take care of themselves: Patients with advanced stages may need help from others for basic life activities such as eating, bathing, and dressing.  Muscle stiffness and immobility: Muscles gradually stiffen, making it difficult to move or even bedridden.  Incontinence: Patients with advanced stage may suffer from incontinence of feces and bladder.  Families can take the following steps to cope with and relieve the symptoms of a person with advanced Alzheimer's disease:  Be patient and understanding: In the face of the patient's plight, family members need to be patient and understanding, and try to avoid accusing and complaining to the patient.  Ensure a safe and comfortable living environment: Create a safe and comfortable living environment for patients to reduce their anxiety and fear.  Simplify Communication: Use simple, unambiguous language and instructions and avoid complex and ambiguous expressions. Try to maintain eye contact with the patient and use body language and gestures to aid communication.  Regular work and rest: Try to keep patients on a regular work and rest time to avoid overwork. You can try to reduce restlessness and restlessness at night with plenty of activity during the day.  Seek professional help: Consider consulting professional doctors and nurses on how to provide the most appropriate care for patients. When necessary, consider choosing a professional nursing facility or enlist professional caregivers to assist with caring for someone with advanced Alzheimer's disease. |
| GPT4 English version:  In the late stage of Alzheimer's disease, a person may experience severe symptoms that can significantly impact their daily life and well-being. Some potential severe symptoms include:  Severe memory loss: Individuals may not recognize family members or close friends and may have difficulty remembering their own personal history.  Communication difficulties: They may lose the ability to communicate coherently or at all, making it difficult to express their needs or understand others.  Mobility issues: There may be a decline in motor skills, making it challenging to walk, sit, or move independently. They may eventually become bedridden.  Incontinence: They may lose control of their bladder or bowel functions.  Eating and swallowing difficulties: This may lead to malnutrition, dehydration, or choking hazards.  Increased susceptibility to infections: They may be more prone to infections, such as pneumonia, due to a weakened immune system and decreased mobility.  Behavioral changes: Individuals may experience agitation, aggression, anxiety, depression, hallucinations, or delusions.  Strategies for family members to manage and alleviate these symptoms include:  Establishing a daily routine: This can provide a sense of structure and predictability, reducing anxiety and agitation.  Ensuring a safe environment: Remove hazards and use assistive devices to prevent falls or injuries. Install bedrails and use specialized utensils to aid in eating.  Communication techniques: Use simple, clear sentences, maintain eye contact, and use nonverbal cues to help facilitate communication.  Personal care assistance: Help with daily activities such as bathing, dressing, and toileting to maintain hygiene and comfort.  Monitoring nutrition and hydration: Offer easy-to-swallow, nutrient-dense foods and ensure regular fluid intake to prevent malnutrition and dehydration.  Managing behavioral changes: Create a calm, soothing environment, and use distraction techniques or gentle touch to alleviate agitation. Consult a healthcare professional for appropriate medications if necessary.  Seeking professional support: Engage healthcare providers, such as geriatricians, neurologists, and palliative care specialists, to provide medical care and symptom management.  Utilizing caregiver support: Seek support from caregiver support groups, respite care services, or counseling to help manage the emotional and practical challenges of caregiving.  It is important for family members to prioritize their own mental and emotional well-being, as caregiving for a loved one with late-stage Alzheimer's can be both physically and emotionally demanding. |
| Neurologist 1:  In the advanced stages of Alzheimer's disease, the memory of patients has been severely impaired, and even their own names and ages can be forgotten. The language expression ability is also basically lost, the speech is fragmented, and the situation of talking to himself often occurs. In addition, the patient almost lost the ability to take care of himself, was unable to move independently, and was incontinent, requiring the care of nursing staff. In advanced stages, the role of caregiver focuses on maintaining the patient's quality of life and dignity. Although people in the advanced stages of Alzheimer's disease often lose their ability to express themselves verbally, long-term research tells us that some core selves of patients still exist, and they also produce feelings. This means you can continue to be emotionally expressive with the patient in the advanced stages of the disease. In addition, caregivers usually need to accompany the whole process of eating, and prompt chewing and swallowing through gestures, mouth shapes, and simple words, such as demonstrating chewing and swallowing movements, while imitating teeth occlusal movements with hands around the mouth, and telling patients "Chew, chew, swallow". In order to maintain the level of digestion, it is necessary to eat small meals as much as possible. It is necessary to allow enough time for the patient to eat and maintain a comfortable, upright position for 30 minutes after the meal to help digestion. |
| Neurologist 2:  Patients with Alzheimer's disease (advanced stage): Unaware of the surrounding environment. Unable to respond, unable to have a conversation (only words). Unable to control actions. Needing help with meals or toileting. Lost smile, couldn't sit down by himself, couldn't look up. Reflexes become abnormal. Muscles become stiff. Decreased swallowing ability. Susceptibility to disease, especially pneumonia.  How are families coping?  1. Keep bones and skin healthy. Hands touching TA must be light; move or turn over every 2 hours to reduce pressure on certain parts of the body; use pillows or cushions to protect joints, and apply body lotion to joints , help TA maintain joint activities every day; keep the skin clean, scrub regularly, be sure to check the skin for abrasions, redness, lumps, etc., pay attention to moisturizing the skin; change the bedding in time, sweep the bed carefully every day, and avoid hard objects , debris damage the skin.  2. Maintain bowel and bladder functions Master the timing of going to the toilet, remind and assist in going to the toilet in time; for frequent incontinence, use diapers, clean and ventilate in time; monitor stool conditions, and adjust diet and drinking water appropriately to avoid prolonged incontinence. Time constipation; check regularly to keep TA clean.  3. Help TAs in the late stage of dementia to no longer pay attention to eating and drinking water. If chewing and swallowing are difficult, the risk of hiccups and choking will increase. It is necessary to: adjust to a sitting position before feeding, and maintain a sitting position for half an hour after eating; give TA plenty of time to eat, do not urge and force; choose And timely adjust the food suitable for TA, such as crushed food, paste food, etc.; before feeding a mouthful, check whether the food has been swallowed; if there is a risk of choking, try to make the liquid thicker; familiarize yourself with the first aid method for choking , always be prepared to prevent the risk of choking.  4. Recognize Pain and Disease Learn to look for signs of pain, such as: pale complexion, flushed complexion, dry and pale gums, mouth sores, vomiting, fever, etc.; do not ignore swelling anywhere in the body, which means something must be happening; observe His gestures, voice, and expressions, and changes in behavior such as anxiety, agitation, shouting, and sleep problems. These can all be signs of pain or discomfort.  5. Prevent infection and pneumonia Keep the mouth and teeth clean, which can reduce the bacteria in the mouth that cause infection; brush your teeth every meal to ensure that the gums, tongue and oral mucosa are clean; drink plenty of water every day to keep your mouth moist; don't let your lips dry; Handle wounds and scars to prevent infection; pay attention to room temperature, put on and take off clothes in time, and keep warm after wiping to prevent colds; in flu season, pay attention to hospital flu, and nurses wash their hands frequently to avoid cross-infection; if nurses have colds or diarrhea, try to Rest and avoid contact with elderly people with poor resistance such as bedridden. |
| Neurologist 3:  Severe symptoms: Patients generally do not know their name and age, let alone their relatives. Patients can only speak simple words, and often practice vocalization, with short, repetitive or rigid speech, or repeated certain sounds, and finally can not speak at all. Occasional vocal responses to painful stimuli. After the language function is lost, the patient gradually loses the ability to walk, cannot stand by himself after sitting down, and the patient can only stay in bed all day long, with urinary and urinary incontinence, and difficulty in eating. During this period, the mental and behavioral symptoms gradually reduced or disappeared. Most patients died of malnutrition, lung infection, bed sores, or other physical diseases within 2 years after entering this period.  Response or mitigation: The patient's family members patiently nurse the patient and conduct regular physical examination and health assessment to maintain the patient's nutritional status. With good nursing and nutritional status, and no other serious physical diseases, they can still survive for a long time. |
| Neurologist 4:  With the progression of Alzheimer's disease, patients gradually lose the ability of daily life and social interaction, and there is a decrease in motor function, nerve function and sensory function, and even circulatory disorders, and can cause systemic diseases. The daily life of patients with advanced Alzheimer's disease is completely dependent on family members or caregivers. Therefore, it is a great challenge for family members to do their best to make patients' lives comfortable.  (1) Eating disorders: Alzheimer's disease patients will have different degrees and forms of eating difficulties during the disease progression, one of the most common complications in advanced Alzheimer's disease patients, mainly including dysphagia, pharyngeal Difficulty swallowing leading to aspiration, inability to eat on their own, or refusal to eat. Insufficient nutrition or unreasonable nutritional mix will affect the health status of patients, leading to adverse consequences such as nutritional disorders. Patients with dysphagia should prevent choking and choking when eating, and a gastric tube can be given for feeding if necessary. However, gastric tube feeding may also increase the risk of lung infection in patients, so family members of patients should fully discuss with doctors and refer to patients' previous opinions and plans to choose as appropriate. When eating problems occur, other means can be used to encourage patients to eat orally, such as eating smaller amounts of food, changing food types, and supplementing with high carbohydrates, etc., if necessary. Alternatively, feeding by a caregiver is optional.  (2) Infection: Infection is a relatively common complication in patients with advanced Alzheimer's disease. About two-thirds of patients have experienced infection in clinical practice, mainly including urinary tract infection or respiratory tract infection. In daily life, it is necessary to dynamically monitor the patient's body temperature changes. When the body temperature rises without obvious infection factors or sources of infection, the possibility of lung infection should be highly suspected and timely medical treatment is required. For the use of antibiotics in patients with advanced Alzheimer's disease, family members need to fully communicate with doctors to indicate the overall treatment goal (for patient comfort or to prolong survival), and professional doctors formulate palliative treatment or add it according to the specific conditions of patients. Antibiotic treatment regimens.  (3) Pressure sores: Pressure sores are also called pressure ulcers in clinical practice. They refer to local tissues that are under pressure for a long time, causing blood circulation disorders, continuous ischemia, hypoxia, and malnutrition, resulting in soft tissue ulceration and necrosis. Patients with advanced severe Alzheimer's disease lose their physiological functions, and will gradually appear unable to change their positions independently, have difficulty eating, and incontinence. In addition, the patient is bedridden for a long time, the local soft tissue is under pressure for a long time, nutritional imbalance, and blood circulation disorder. There is also an increased risk of developing pressure sores. If it is not detected in time or improper measures are taken, it will make it difficult for the wound to heal, and in severe cases, it may be life-threatening due to secondary infection. Predilection sites for pressure ulcers are as follows: Lateral position: auricle, acromion, elbow, hip, medial and lateral knee joint, medial and lateral ankle joint. Supine position: occipital tuberosity, scapula, elbow, sacrum, especially sacrum. Prone position: cheeks, auricles, acromion, female breasts, costal margin protrusion, anterior superior iliac spine, male genitalia, knees, toes, etc. Sitting position: ischial tuberosity, scapula, heel, the contact point between the back of the knee and the edge of the chair, etc. Prevention is the primary care for pressure sores, and family members or caregivers should help patients turn over frequently and keep them clean. At the same time, it is necessary to regularly check the skin of the patient's pressure part to protect the integrity of the patient's skin. A decompression mattress can also be used according to the patient's condition to help prevent pressure sores. Once a pressure sore occurs, local tissue inflammation, blisters, or even epidermal damage occur, it is recommended to take the patient to see a doctor in time and be treated by professional medical staff.  (4) Sleep disorders: In the late stages of Alzheimer's disease, due to abnormal hormone levels in the body, sleep rhythm disorders may occur. If you have a habit of napping during the day, you will sleep less at night. As the disease progresses, the sleep rhythm disorder will become more obvious, manifested as sleep disorders, such as the gradual indistinguishability of day and night, and no fixed sleep time. It is recommended that family members or caregivers help the elderly to adjust their sleep and minimize the time and frequency of naps during the day. In addition, it can also promote the sleep of patients by creating a quiet sleeping environment, allowing patients to listen to soothing music, soak their feet, and perform back massage to relax their bodies before going to bed. If necessary, you can also take sleep aid drugs as directed by your doctor to promote sleep.  (5) Joint deformities and muscle atrophy: Bedridden patients should regularly perform passive activities of limb joints, often stretch and massage muscles. At the same time, they can also consult professionals to prevent joint deformities and muscle atrophy through acupuncture and electrical stimulation. During this period, family members need to be patient, considerate to the patient, and avoid loud reprimands, so as not to hurt the patient's self-esteem and aggravate the deterioration of the condition. In the care of patients with severe Alzheimer's disease, in addition to discussing treatment and nursing plans with doctors, it is also necessary to fully discuss and formulate end-of-life care plans based on the specific conditions of the patients. Taking care of patients with severe Alzheimer's disease is a heavy and long-term job. Family members or caregivers need to pay a lot of patience, which will change the original life and work status, and will require greater physical strength in the later stage. Therefore, those who take care of patients need to have a strong mentality to accept this companionship process. In daily life, they should pay attention to regulating emotions, maintain an optimistic attitude, communicate more with relatives and friends, and leave an appropriate amount of time and space for themselves to relieve stress.  In addition, family members need to pay attention to providing a safe, stable, and appropriately stimulating living environment for patients with Alzheimer's disease, and take good care of the daily life of patients with Alzheimer's disease. They can also use available assistive technologies, such as the elderly phone with convenient buttons Machines, fully automatic calendar clocks, GPS anti-lost trackers, multimedia software, wristband electronic tags, home monitoring equipment if necessary, and so on. At the same time, physical and cognitive rehabilitation training should be combined with nursing care to help delay the cognitive decline of patients. |

Table S3. Eight items on the clinician evaluation questionnaire.

| **Item** | |  |
| --- | --- | --- |
| **Accuracy** | |  |
| S1. These answers are completely correct and accurate. | |  |
| **Comprehensiveness** | |  |
| S2. These answers are very comprehensive and complete (able to satisfy the needs of patient family members). | |  |
| **Comprehensibility** | |  |
| S3. These answers are very clear. | |  |
| S4. These answers are easy for patient family members to understand. | |  |
| S7. I believe these answers are very helpful to patients and their family members. | |  |
| **Satisfaction** | |  |
| S5. Overall, I am very satisfied with these answers. | |  |
| S6. I would recommend the content of this response to other doctors or professionals. | |  |
| S8. I would use this content in patient health education. | |  |
|  |  |  |

Table S4. Eight items on the patient’s family evaluation questionnaire.

| **Item** | |  |  |
| --- | --- | --- | --- |
| **Comprehensibility** | |  |  |
| I1. These answers are clear and easy to understand, giving me a better understanding of Alzheimer's disease. | |  |  |
| I2. These answers address my doubts and concerns about Alzheimer's disease. | |  |  |
| I3. The suggestions in these answers are easy to implement, and I will follow the advice provided. | |  |  |
| **Usefulness** | |  |  |
| I4. These answers can help me care for and look after Alzheimer's patients. | |  |  |
| I6. These answers are very helpful for the treatment or prevention of the disease for patients. | |  |  |
| **Satisfaction** | |  |  |
| I5. These answers completely cover the questions I wanted to know. | |  |  |
| I7. Overall, I am very satisfied with these answers. | |  |  |
| I8. I would not hesitate to recommend these answers to other patient family members or friends. | |  |  |
|  |  |  |  |

Table S5. Top 10 responses rated by clinicians. N: neurologists-generated suggestions; G: ChatGPT-generated suggestions.

|  | **Question** | | **Satisfied** | **Dissatisfied** | **Overall** | | |  | |
| --- | --- | --- | --- | --- | --- | --- | --- | --- | --- |
| G | Q5. How to prevent and manage memory impairment in patients with Alzheimer's disease? | | 12 | 0 | 12 | | |  |  |
| G | Q8. What types of nursing and care are necessary for patients with Alzheimer's disease? | | 11 | 0 | 11 | | |  |  |
| G | Q12. How can Alzheimer's disease patients receive psychological and emotional support? | | 8 | 0 | 8 | | |  |  |
| G | Q10. What are the symptoms that Alzheimer's disease patients should look out and go to the emergency room for? | | 8 | 0 | 8 | | |  |  |
| N | Q12. How can Alzheimer's disease patients receive psychological and emotional support? | | 7 | 0 | 7 | | |  |  |
| G | Q3. What are the symptoms that Alzheimer's disease patients should look out and go to the emergency room for? | | 7 | 0 | 7 | | |  |  |
| G | Q9. What strategies can be used to provide nutritional support for patients with Alzheimer's disease? | | 7 | 0 | 7 | | |  |  |
| N | Q10. What are the symptoms that Alzheimer's disease patients should look out and go to the emergency room for? | | 6 | 0 | 6 | | |  |  |
| G | Q14. How to determine the stage of Alzheimer's disease progression in a patient? | | 7 | 2 | 5 | | |  |  |
| G | Q11. How to help the families of late-stage Alzheimer's disease patients cope with the stress of daily living and caregiving? | | 4 | 0 | 4 | | |  |  |
|  |  |  | | | |  |  | |  |

Table S6. Top 10 responses rated by patient’s family. N: neurologists-generated suggestions; G: ChatGPT-generated suggestions.

|  | **Question** | | **Satisfied** | **Dissatisfied** | **Overall** | | |  | |
| --- | --- | --- | --- | --- | --- | --- | --- | --- | --- |
| G | Q8. What types of nursing and care are necessary for patients with Alzheimer's disease? | | 15 | 0 | 15 | | |  |  |
| G | Q10. What are the symptoms that Alzheimer's disease patients should look out and go to the emergency room for? | | 9 | 0 | 9 | | |  |  |
| N | Q12. How can Alzheimer's disease patients receive psychological and emotional support? | | 9 | 0 | 9 | | |  |  |
| G | Q1. How to prevent Alzheimer's disease? Please provide me alternative suggestions from the aspects of medications, lifestyle, and diet. | | 11 | 2 | 9 | | |  |  |
| G | Q4. What is the role of regular physical examination and health assessments help the prevention of Alzheimer's disease? | | 11 | 2 | 9 | | |  |  |
| G | Q2. What are the causes or risk factors for Alzheimer's disease? | | 10 | 2 | 8 | | |  |  |
| G | Q3. If a parent had/has Alzheimer's disease, are their children at greater risk of getting it? Is Alzheimer's disease hereditary? | | 9 | 1 | 8 | | |  |  |
| N | Q14. How to determine the stage of Alzheimer's disease progression in a patient? | | 9 | 1 | 8 | | |  |  |
| G | Q13. How to determine the stage of Alzheimer's disease progression in a patient? | | 7 | 0 | 7 | | |  |  |
| G | Q12. How can Alzheimer's disease patients receive psychological and emotional support? | | 9 | 2 | 7 | | |  |  |
|  |  |  | | | |  |  | |  |

Table S7. Means and SD of clinicians’ ratings for survey items using a five-point Likert scale (1—strongly disagree, 5—strongly agree)

|  | | **ChatGPT-generated responses**  **(mean ± SD**) | **Neurologists-generated responses**  **(mean ± SD)** | ***p-value*** |  |
| --- | --- | --- | --- | --- | --- |
| **Accuracy** | |  |  |  |  |
| S1. These answers are completely correct and accurate. | | 4·3 ± 0·6 | 3·7 ± 0·8 | 0·0096 |  |
| **Comprehensiveness** | |  |  |  |  |
| S2. These answers are very comprehensive and complete (able to satisfy the needs of patient family members). | 4·4 ± 0·6 | | 4·0 ± 0·7 | 0·055 |  |
| **Comprehensibility** | |  |  |  |  |
| S3. These answers are very clear. | | 4·4 ± 0·6 | 3·8 ± 0·8 | 0·021 |  |
| S4. These answers are easy for patient family members to understand. | | 4·2 ± 0·8 | 4·0 ± 0·8 | 0·33 |  |
| S7. I believe these answers are very helpful to patients and their family members. | | 4·6 ± 0·5 | 4·0 ± 0·7 | 0·0033 |  |
| **Satisfaction** | |  |  |  |  |
| S5. Overall, I am very satisfied with these answers. | | 4·3 ± 0·6 | 4·0 ± 0·5 | 0·082 |  |
| S6. I would recommend the content of this response to other doctors or professionals. | | 4·3 ± 0·6 | 4·0 ± 0·6 | 0·11 |  |
| S8. I would use this content in patient health education· | | 4·6 ± 0·5 | 4·0 ± 0·6 | 0·0025 |  |
| **Overall** | | **4·4 ± 0·6** | **3·9 ± 0·7** | **<0·0001** |  |
|  | | | | | |

|  | | **GPT3-generated responses**  **(mean ± SD**) | **GPT4-generated responses**  **(mean ± SD)** | ***p-value*** |  |
| --- | --- | --- | --- | --- | --- |
| **Accuracy** | |  |  |  |  |
| S1. These answers are completely correct and accurate. | | 4·1 ± 0·6 | 4·5 ± 0·5 | 0·12 |  |
| **Comprehensiveness** | |  |  |  |  |
| S2. These answers are very comprehensive and complete (able to satisfy the needs of patient family members). | 4·2 ± 0·6 | | 4·5 ± 0·5 | 0·26 |  |
| **Comprehensibility** | |  |  |  |  |
| S3. These answers are very clear. | | 4·4 ± 0·7 | 4·3 ± 0·5 | 0·71 |  |
| S4. These answers are easy for patient family members to understand. | | 3·9 ± 0·9 | 4·5 ± 0·5 | 0·079 |  |
| S7. I believe these answers are very helpful to patients and their family members. | | 4·6 ± 0·5 | 4·5 ± 0·5 | 0·67 |  |
| **Satisfaction** | |  |  |  |  |
| S5. Overall, I am very satisfied with these answers. | | 4·2 ± 0·6 | 4·3 ± 0·5 | 0·70 |  |
| S6. I would recommend the content of this response to other doctors or professionals. | | 4·3 ± 0·7 | 4·2 ± 0·4 | 0·70 |  |
| S8. I would use this content in patient health education. | | 4·7 ± 0·5 | 4·4 ± 0·5 | 0·20 |  |
| **Overall** | | **4·3 ± 0·7** | **4·4 ± 0·5** | **0·51** |  |
|  | | | | | |

|  | | **Chinese responses**  **(mean ± SD**) | **English responses**  **(mean ± SD)** | ***p-value*** |  |
| --- | --- | --- | --- | --- | --- |
| **Accuracy** |  | |  |  |  |
| S1. These answers are completely correct and accurate. | 4·3 ± 0·7 | | 4·3 ± 0·5 | 1.0 |  |
| **Comprehensiveness** |  | |  |  |  |
| S2. These answers are very comprehensive and complete (able to satisfy the needs of patient family members). | 4·3 ± 0·7 | | 4·4 ± 0·5 | 0·71 |  |
| **Comprehensibility** |  | |  |  |  |
| S3. These answers are very clear. | | 4·3 ± 0·7 | 4·4 ± 0·5 | 0·71 |  |
| S4. These answers are easy for patient family members to understand. | | 3·8 ± 0·8 | 4·6 ± 0·5 | 0·015 |  |
| S7. I believe these answers are very helpful to patients and their family members. | | 4·3 ± 0·5 | 4·8 ± 0·4 | 0·024 |  |
| **Satisfaction** |  | |  |  |  |
| S5. Overall, I am very satisfied with these answers. | | 4·1 ± 0·6 | 4·4 ± 0·5 | 0·23 |  |
| S6. I would recommend the content of this response to other doctors or professionals. | | 4·1 ± 0·6 | 4·4 ± 0·5 | 0·23 |  |
| S8. I would use this content in patient health education. | | 4·5 ± 0·5 | 4·7 ± 0·5 | 0·20 |  |
| **Overall** | | **4·4 ± 0·6** | **4·5 ± 0·5** | **0·0023** |  |
|  | | | | | |

|  | | **ChatGPT-generated responses**  **(mean ± SD**) | **Neurologists-generated responses**  **(mean ± SD)** | ***p-value*** |  |
| --- | --- | --- | --- | --- | --- |
| **Comprehensibility** |  | |  |  |  |
| I1. These answers are clear and easy to understand, giving me a better understanding of Alzheimer's disease. | 4.2 ± 0.5 | | 4.0 ± 0.6 | 0.29 |  |
| I2. These answers address my doubts and concerns about Alzheimer's disease. | 4.0 ± 0.5 | | 3.9 ± 0.9 | 0.52 |  |
| I3. The suggestions in these answers are easy to implement, and I will follow the advice provided. | | 3.8 ± 0.6 | 3.9 ± 0.6 | 0.46 |  |
| **Usefulness** |  | |  |  |  |
| I4. These answers can help me care for and look after Alzheimer's patients. | | 4.1 ± 0.3 | 4.1 ± 0.6 | 0.74 |  |
| I6. These answers are very helpful for the treatment or prevention of the disease for patients. | | 3.7 ± 0.6 | 3.9 ± 0.7 | 0.34 |  |
| **Satisfaction** |  | |  |  |  |
| I5. These answers completely cover the questions I wanted to know. | | 3.5 ± 0.5 | 3.6 ± 0.8 | 0.81 |  |
| I7. Overall, I am very satisfied with these answers. | | 4.0 ± 0.6 | 3.8 ± 0.8 | 0.37 |  |
| I8. I would not hesitate to recommend these answers to other patient family members or friends. | | 3.8 ± 0.6 | 3.9 ± 1.1 | 0.59 |  |
| **Overall** | | **3.9 ± 0.6** | **3.9 ± 0.8** | **0.75** |  |
|  | | | | | |

Table S8. Means and SD of patient’s family ratings for survey items using a five-point Likert scale (1—strongly disagree, 5—strongly agree).

|  | | **GPT3-generated responses**  **(mean ± SD**) | **GPT4-generated responses**  **(mean ± SD)** | ***p-value*** |  |
| --- | --- | --- | --- | --- | --- |
| **Comprehensibility** | |  |  |  |  |
| I1. These answers are clear and easy to understand, giving me a better understanding of Alzheimer's disease. | | 4.4 ± 0.5 | 4.0 ± 0.5 | 0.087 |  |
| I2. These answers address my doubts and concerns about Alzheimer's disease. | 4.0 ± 0.5 | | 4.0 ± 0.5 | 1.0 |  |
| I3. The suggestions in these answers are easy to implement, and I will follow the advice provided. | | 3.8 ± 0.6 | 3.7 ± 0.7 | 0.74 |  |
| **Usefulness** | |  |  |  |  |
| I4. These answers can help me care for and look after Alzheimer's patients. | | 4.1 ± 0.3 | 4.1 ± 0.3 | 1.0 |  |
| I6. These answers are very helpful for the treatment or prevention of the disease for patients. | | 3.5 ± 0.5 | 3.9 ± 0.6 | 0.12 |  |
| **Satisfaction** | |  |  |  |  |
| I5. These answers completely cover the questions I wanted to know. | | 3.5 ± 0.5 | 3.5 ± 0.5 | 1.0 |  |
| I7. Overall, I am very satisfied with these answers. | | 3.8 ± 0.6 | 4.1 ± 0.6 | 0.28 |  |
| I8. I would not hesitate to recommend these answers to other patient family members or friends. | | 3.7 ± 0.7 | 3.8 ± 0.6 | 0.74 |  |
| **Overall** | | **3.9 ± 0.6** | **3.9 ± 0.6** | **0.65** |  |
|  | | | | | |

|  | | **Chinese responses**  **(mean ± SD**) | **English responses**  **(mean ± SD)** | ***p-value*** |  |
| --- | --- | --- | --- | --- | --- |
| **Comprehensibility** | |  |  |  |  |
| I1. These answers are clear and easy to understand, giving me a better understanding of Alzheimer's disease. | | 4.3 ± 0.5 | 4.1 ± 0.6 | 0.41 |  |
| I2. These answers address my doubts and concerns about Alzheimer's disease. | 4.0 ± 0.5 | | 4.0 ± 0.5 | 1.0 |  |
| I3. The suggestions in these answers are easy to implement, and I will follow the advice provided. | | 3.9 ± 0.7 | 3.6 ± 0.5 | 0.31 |  |
| **Usefulness** | |  |  |  |  |
| I4. These answers can help me care for and look after Alzheimer's patients. | | 4.1 ± 0.3 | 4.1 ± 0.3 | 1.0 |  |
| I6. These answers are very helpful for the treatment or prevention of the disease for patients. | | 3.6 ± 0.7 | 3.8 ± 0.4 | 0.45 |  |
| **Satisfaction** | |  |  |  |  |
| I5. These answers completely cover the questions I wanted to know. | | 3.3 ± 0.5 | 3.7 ± 0.5 | 0.081 |  |
| I7. Overall, I am very satisfied with these answers. | | 3.9 ± 0.6 | 4.0 ± 0.7 | 0.72 |  |
| I8. I would not hesitate to recommend these answers to other patient family members or friends. | | 3.7 ± 0.7 | 3.8 ± 0.6 | 0.73 |  |
| **Overall** | | **3.9 ± 0.6** | **3.9 ± 0.5** | **0.62** |  |
|  | | | | | |
