# Supplementary material for: Assessing the Role of the Generative Pretrained Transformer (GPT) in Alzheimer’s Disease Management: Comparative Study of Neurologist- and Artificial Intelligence–Generated Responses
Source: J Med Internet Res. 2024 Oct 31;26:e51095. doi: 10.2196/51095 (PMC11565080; doi:10.2196/51095)
Supplement: Multimedia Appendix 3 [file jmir_v26i1e51095_app3.docx]

**Translation Development Process**

In this study, the English-language questionnaires and responses were carefully translated into Chinese by certified professionals. All participating neurologists had study experience in English-speaking countries and experience in English translation. This process was designed to ensure not only linguistic accuracy but also cultural relevance, thereby bridging any potential language barriers for participants. The translation methodology included the following stages:

**Independent Translation Phase**

The first step was to select two neurologists to independently translate the text into Chinese. An English expert with medical translation experience evaluated and revised the translations of the two neurologists. After discussion and research, a preliminary draft of the Chinese version was produced.

**Back Translation Technique**

To ensure that the translated text was as close as possible to the original, while respecting Chinese cultural norms and linguistic conventions, we invited a neurologist to carry out a back-translation. The back-translated content was then carefully compared with the original content and modified.

**Revision and Enhancement Stage**

To validate the Chinese version, we asked a professor of neurology to rate each item in the Chinese version and provide feedback for revisions. Based on the professor's feedback, further changes and improvements were made.

**Testing Phase**

Once the translated version was complete, we invited a person with Alzheimer's disease (AD), a family member of someone with AD and a healthcare provider to review the content and assess its readability and overall understanding.

In conclusion, this study used a careful translation and rigorous review process to ensure linguistic accuracy and cultural relevance, including independent translation, back-translation, revision and improvement, and testing to minimize language barriers for participants.
