# Supplementary material for: Assessing the Role of the Generative Pretrained Transformer (GPT) in Alzheimer’s Disease Management: Comparative Study of Neurologist- and Artificial Intelligence–Generated Responses
Source: J Med Internet Res. 2024 Oct 31;26:e51095. doi: 10.2196/51095 (PMC11565080; doi:10.2196/51095)
Supplement: Multimedia Appendix 4 [file jmir_v26i1e51095_app4.doc]

Case Processing Summary	
	Group	Cases	
		Valid	Missing	Total	
		N	Percent	N	Percent	N	Percent	
GPT	A	20	100.0%	0	0.0%	20	100.0%	
	C1	20	100.0%	0	0.0%	20	100.0%	
	C2	60	100.0%	0	0.0%	60	100.0%	
	S	60	100.0%	0	0.0%	60	100.0%	
Human	A	20	100.0%	0	0.0%	20	100.0%	
	C1	20	100.0%	0	0.0%	20	100.0%	
	C2	60	100.0%	0	0.0%	60	100.0%	
	S	60	100.0%	0	0.0%	60	100.0%	


Descriptivesa,b	
	Group	Statistic	Std. Error	
GPT	A	Mean	4.300	.1277	
		95% Confidence Interval for Mean	Lower Bound	4.033		
			Upper Bound	4.567		
		5% Trimmed Mean	4.333		
		Median	4.000		
		Variance	.326		
		Std. Deviation	.5712		
		Minimum	3.0		
		Maximum	5.0		
		Range	2.0		
		Interquartile Range	1.0		
		Skewness	-.038	.512	
		Kurtosis	-.395	.992	
	C1	Mean	4.350	.1313	
		95% Confidence Interval for Mean	Lower Bound	4.075		
			Upper Bound	4.625		
		5% Trimmed Mean	4.389		
		Median	4.000		
		Variance	.345		
		Std. Deviation	.5871		
		Minimum	3.0		
		Maximum	5.0		
		Range	2.0		
		Interquartile Range	1.0		
		Skewness	-.212	.512	
		Kurtosis	-.552	.992	
	C2	Mean	4.367	.0822	
		95% Confidence Interval for Mean	Lower Bound	4.202		
			Upper Bound	4.531		
		5% Trimmed Mean	4.426		
		Median	4.000		
		Variance	.406		
		Std. Deviation	.6369		
		Minimum	2.0		
		Maximum	5.0		
		Range	3.0		
		Interquartile Range	1.0		
		Skewness	-.902	.309	
		Kurtosis	1.755	.608	
	S	Mean	4.350	.0706	
		95% Confidence Interval for Mean	Lower Bound	4.209		
			Upper Bound	4.491		
		5% Trimmed Mean	4.370		
		Median	4.000		
		Variance	.299		
		Std. Deviation	.5469		
		Minimum	3.0		
		Maximum	5.0		
		Range	2.0		
		Interquartile Range	1.0		
		Skewness	-.011	.309	
		Kurtosis	-.795	.608	
Human	A	Mean	3.700	.1792	
		95% Confidence Interval for Mean	Lower Bound	3.325		
			Upper Bound	4.075		
		5% Trimmed Mean	3.722		
		Median	4.000		
		Variance	.642		
		Std. Deviation	.8013		
		Minimum	2.0		
		Maximum	5.0		
		Range	3.0		
		Interquartile Range	.0		
		Skewness	-1.418	.512	
		Kurtosis	1.415	.992	
	C1	Mean	3.950	.1535	
		95% Confidence Interval for Mean	Lower Bound	3.629		
			Upper Bound	4.271		
		5% Trimmed Mean	4.000		
		Median	4.000		
		Variance	.471		
		Std. Deviation	.6863		
		Minimum	2.0		
		Maximum	5.0		
		Range	3.0		
		Interquartile Range	.0		
		Skewness	-1.023	.512	
		Kurtosis	2.886	.992	
	C2	Mean	3.900	.1000	
		95% Confidence Interval for Mean	Lower Bound	3.700		
			Upper Bound	4.100		
		5% Trimmed Mean	3.944		
		Median	4.000		
		Variance	.600		
		Std. Deviation	.7746		
		Minimum	2.0		
		Maximum	5.0		
		Range	3.0		
		Interquartile Range	.0		
		Skewness	-.955	.309	
		Kurtosis	1.206	.608	
	S	Mean	3.967	.0712	
		95% Confidence Interval for Mean	Lower Bound	3.824		
			Upper Bound	4.109		
		5% Trimmed Mean	4.000		
		Median	4.000		
		Variance	.304		
		Std. Deviation	.5513		
		Minimum	2.0		
		Maximum	5.0		
		Range	3.0		
		Interquartile Range	.0		
		Skewness	-1.277	.309	
		Kurtosis	5.017	.608	

a. There are no valid cases for GPT when Group = .000. Statistics cannot be computed for this level.	
b. There are no valid cases for Human when Group = .000. Statistics cannot be computed for this level.	


Tests of Normalitya,c	
	Group	Kolmogorov-Smirnovb	Shapiro-Wilk	
		Statistic	df	Sig.	Statistic	df	Sig.	
GPT	A	.350	20	.000	.736	20	.000	
	C1	.324	20	.000	.744	20	.000	
	C2	.284	60	.000	.730	60	.000	
	S	.356	60	.000	.710	60	.000	
Human	A	.446	20	.000	.635	20	.000	
	C1	.379	20	.000	.741	20	.000	
	C2	.368	60	.000	.759	60	.000	
	S	.424	60	.000	.609	60	.000	

a. There are no valid cases for GPT when Group = .000. Statistics cannot be computed for this level.	
b. Lilliefors Significance Correction	
c. There are no valid cases for Human when Group = .000. Statistics cannot be computed for this level.	


GPT


Histograms


Stem-and-Leaf Plots


GPT Stem-and-Leaf Plot for
Group= A

 Frequency    Stem &  Leaf

     1.00        3 .  0
      .00        3 .
    12.00        4 .  000000000000
      .00        4 .
     7.00        5 .  0000000

 Stem width:  1.0
 Each leaf:        1 case(s)


GPT Stem-and-Leaf Plot for
Group= C1

 Frequency    Stem &  Leaf

     1.00        3 .  0
      .00        3 .
    11.00        4 .  00000000000
      .00        4 .
     8.00        5 .  00000000

 Stem width:  1.0
 Each leaf:        1 case(s)


GPT Stem-and-Leaf Plot for
Group= C2

 Frequency    Stem &  Leaf

     1.00 Extremes    (=<2.0)
     2.00        3 .  00
      .00        3 .
    31.00        4 .  0000000000000000000000000000000
      .00        4 .
    26.00        5 .  00000000000000000000000000

 Stem width:  1.0
 Each leaf:        1 case(s)


GPT Stem-and-Leaf Plot for
Group= S

 Frequency    Stem &  Leaf

     2.00        3 .  00
      .00        3 .
    35.00        4 .  00000000000000000000000000000000000
      .00        4 .
    23.00        5 .  00000000000000000000000

 Stem width:  1.0
 Each leaf:        1 case(s)


Normal Q-Q Plots


Detrended Normal Q-Q Plots


Human


Histograms


Stem-and-Leaf Plots


Human Stem-and-Leaf Plot for
Group= A

 Frequency    Stem &  Leaf

     4.00 Extremes    (=<3)
      .00        0 .
    15.00        0 .  444444444444444
     1.00 Extremes    (>=5)

 Stem width:   10
 Each leaf:        1 case(s)


Human Stem-and-Leaf Plot for
Group= C1

 Frequency    Stem &  Leaf

     3.00 Extremes    (=<3)
      .00        0 .
    14.00        0 .  44444444444444
     3.00 Extremes    (>=5)

 Stem width:   10
 Each leaf:        1 case(s)


Human Stem-and-Leaf Plot for
Group= C2

 Frequency    Stem &  Leaf

    11.00 Extremes    (=<3)
      .00        0 .
    39.00        0 .  444444444444444444444444444444444444444
    10.00 Extremes    (>=5)

 Stem width:   10
 Each leaf:        1 case(s)


Human Stem-and-Leaf Plot for
Group= S

 Frequency    Stem &  Leaf

     6.00 Extremes    (=<3)
      .00        0 .
    48.00        0 .  444444444444444444444444444444444444444444444444
     6.00 Extremes    (>=5)

 Stem width:   10
 Each leaf:        1 case(s)


Normal Q-Q Plots


Detrended Normal Q-Q Plots


Case Processing Summary	
	Group2	Cases	
		Valid	Missing	Total	
		N	Percent	N	Percent	N	Percent	
GPT_3	A	10	100.0%	0	0.0%	10	100.0%	
	C1	10	100.0%	0	0.0%	10	100.0%	
	C2	30	100.0%	0	0.0%	30	100.0%	
	S	30	100.0%	0	0.0%	30	100.0%	
GPT_4	A	10	100.0%	0	0.0%	10	100.0%	
	C1	10	100.0%	0	0.0%	10	100.0%	
	C2	30	100.0%	0	0.0%	30	100.0%	
	S	30	100.0%	0	0.0%	30	100.0%	
Chinese	A	10	100.0%	0	0.0%	10	100.0%	
	C1	10	100.0%	0	0.0%	10	100.0%	
	C2	30	100.0%	0	0.0%	30	100.0%	
	S	30	100.0%	0	0.0%	30	100.0%	
English	A	10	100.0%	0	0.0%	10	100.0%	
	C1	10	100.0%	0	0.0%	10	100.0%	
	C2	30	100.0%	0	0.0%	30	100.0%	
	S	30	100.0%	0	0.0%	30	100.0%	


Descriptivesa,b,c,d	
	Group2	Statistic	Std. Error	
GPT_3	A	Mean	4.100	.1795	
		95% Confidence Interval for Mean	Lower Bound	3.694		
			Upper Bound	4.506		
		5% Trimmed Mean	4.111		
		Median	4.000		
		Variance	.322		
		Std. Deviation	.5676		
		Minimum	3.0		
		Maximum	5.0		
		Range	2.0		
		Interquartile Range	.3		
		Skewness	.091	.687	
		Kurtosis	1.498	1.334	
	C1	Mean	4.200	.2000	
		95% Confidence Interval for Mean	Lower Bound	3.748		
			Upper Bound	4.652		
		5% Trimmed Mean	4.222		
		Median	4.000		
		Variance	.400		
		Std. Deviation	.6325		
		Minimum	3.0		
		Maximum	5.0		
		Range	2.0		
		Interquartile Range	1.0		
		Skewness	-.132	.687	
		Kurtosis	.179	1.334	
	C2	Mean	4.300	.1369	
		95% Confidence Interval for Mean	Lower Bound	4.020		
			Upper Bound	4.580		
		5% Trimmed Mean	4.370		
		Median	4.000		
		Variance	.562		
		Std. Deviation	.7497		
		Minimum	2.0		
		Maximum	5.0		
		Range	3.0		
		Interquartile Range	1.0		
		Skewness	-1.094	.427	
		Kurtosis	1.621	.833	
	S	Mean	4.400	.1135	
		95% Confidence Interval for Mean	Lower Bound	4.168		
			Upper Bound	4.632		
		5% Trimmed Mean	4.444		
		Median	4.000		
		Variance	.386		
		Std. Deviation	.6215		
		Minimum	3.0		
		Maximum	5.0		
		Range	2.0		
		Interquartile Range	1.0		
		Skewness	-.517	.427	
		Kurtosis	-.534	.833	
GPT_4	A	Mean	4.500	.1667	
		95% Confidence Interval for Mean	Lower Bound	4.123		
			Upper Bound	4.877		
		5% Trimmed Mean	4.500		
		Median	4.500		
		Variance	.278		
		Std. Deviation	.5270		
		Minimum	4.0		
		Maximum	5.0		
		Range	1.0		
		Interquartile Range	1.0		
		Skewness	.000	.687	
		Kurtosis	-2.571	1.334	
	C1	Mean	4.500	.1667	
		95% Confidence Interval for Mean	Lower Bound	4.123		
			Upper Bound	4.877		
		5% Trimmed Mean	4.500		
		Median	4.500		
		Variance	.278		
		Std. Deviation	.5270		
		Minimum	4.0		
		Maximum	5.0		
		Range	1.0		
		Interquartile Range	1.0		
		Skewness	.000	.687	
		Kurtosis	-2.571	1.334	
	C2	Mean	4.433	.0920	
		95% Confidence Interval for Mean	Lower Bound	4.245		
			Upper Bound	4.622		
		5% Trimmed Mean	4.426		
		Median	4.000		
		Variance	.254		
		Std. Deviation	.5040		
		Minimum	4.0		
		Maximum	5.0		
		Range	1.0		
		Interquartile Range	1.0		
		Skewness	.283	.427	
		Kurtosis	-2.062	.833	
	S	Mean	4.300	.0851	
		95% Confidence Interval for Mean	Lower Bound	4.126		
			Upper Bound	4.474		
		5% Trimmed Mean	4.278		
		Median	4.000		
		Variance	.217		
		Std. Deviation	.4661		
		Minimum	4.0		
		Maximum	5.0		
		Range	1.0		
		Interquartile Range	1.0		
		Skewness	.920	.427	
		Kurtosis	-1.242	.833	
Chinese	A	Mean	4.300	.2134	
		95% Confidence Interval for Mean	Lower Bound	3.817		
			Upper Bound	4.783		
		5% Trimmed Mean	4.333		
		Median	4.000		
		Variance	.456		
		Std. Deviation	.6749		
		Minimum	3.0		
		Maximum	5.0		
		Range	2.0		
		Interquartile Range	1.0		
		Skewness	-.434	.687	
		Kurtosis	-.283	1.334	
	C1	Mean	4.300	.2134	
		95% Confidence Interval for Mean	Lower Bound	3.817		
			Upper Bound	4.783		
		5% Trimmed Mean	4.333		
		Median	4.000		
		Variance	.456		
		Std. Deviation	.6749		
		Minimum	3.0		
		Maximum	5.0		
		Range	2.0		
		Interquartile Range	1.0		
		Skewness	-.434	.687	
		Kurtosis	-.283	1.334	
	C2	Mean	4.133	.1244	
		95% Confidence Interval for Mean	Lower Bound	3.879		
			Upper Bound	4.388		
		5% Trimmed Mean	4.185		
		Median	4.000		
		Variance	.464		
		Std. Deviation	.6814		
		Minimum	2.0		
		Maximum	5.0		
		Range	3.0		
		Interquartile Range	1.0		
		Skewness	-.871	.427	
		Kurtosis	2.275	.833	
	S	Mean	4.200	.1006	
		95% Confidence Interval for Mean	Lower Bound	3.994		
			Upper Bound	4.406		
		5% Trimmed Mean	4.222		
		Median	4.000		
		Variance	.303		
		Std. Deviation	.5509		
		Minimum	3.0		
		Maximum	5.0		
		Range	2.0		
		Interquartile Range	1.0		
		Skewness	.106	.427	
		Kurtosis	.097	.833	
English	A	Mean	4.300	.1528	
		95% Confidence Interval for Mean	Lower Bound	3.954		
			Upper Bound	4.646		
		5% Trimmed Mean	4.278		
		Median	4.000		
		Variance	.233		
		Std. Deviation	.4830		
		Minimum	4.0		
		Maximum	5.0		
		Range	1.0		
		Interquartile Range	1.0		
		Skewness	1.035	.687	
		Kurtosis	-1.224	1.334	
	C1	Mean	4.400	.1633	
		95% Confidence Interval for Mean	Lower Bound	4.031		
			Upper Bound	4.769		
		5% Trimmed Mean	4.389		
		Median	4.000		
		Variance	.267		
		Std. Deviation	.5164		
		Minimum	4.0		
		Maximum	5.0		
		Range	1.0		
		Interquartile Range	1.0		
		Skewness	.484	.687	
		Kurtosis	-2.277	1.334	
	C2	Mean	4.600	.0910	
		95% Confidence Interval for Mean	Lower Bound	4.414		
			Upper Bound	4.786		
		5% Trimmed Mean	4.611		
		Median	5.000		
		Variance	.248		
		Std. Deviation	.4983		
		Minimum	4.0		
		Maximum	5.0		
		Range	1.0		
		Interquartile Range	1.0		
		Skewness	-.430	.427	
		Kurtosis	-1.950	.833	
	S	Mean	4.500	.0928	
		95% Confidence Interval for Mean	Lower Bound	4.310		
			Upper Bound	4.690		
		5% Trimmed Mean	4.500		
		Median	4.500		
		Variance	.259		
		Std. Deviation	.5085		
		Minimum	4.0		
		Maximum	5.0		
		Range	1.0		
		Interquartile Range	1.0		
		Skewness	.000	.427	
		Kurtosis	-2.148	.833	

a. There are no valid cases for GPT_3 when Group2 = .000. Statistics cannot be computed for this level.	
b. There are no valid cases for GPT_4 when Group2 = .000. Statistics cannot be computed for this level.	
c. There are no valid cases for Chinese when Group2 = .000. Statistics cannot be computed for this level.	
d. There are no valid cases for English when Group2 = .000. Statistics cannot be computed for this level.	


Tests of Normalitya,c,d,e	
	Group2	Kolmogorov-Smirnovb	Shapiro-Wilk	
		Statistic	df	Sig.	Statistic	df	Sig.	
GPT_3	A	.370	10	.000	.752	10	.004	
	C1	.324	10	.004	.794	10	.012	
	C2	.258	30	.000	.769	30	.000	
	S	.300	30	.000	.749	30	.000	
GPT_4	A	.329	10	.003	.655	10	.000	
	C1	.329	10	.003	.655	10	.000	
	C2	.372	30	.000	.632	30	.000	
	S	.440	30	.000	.577	30	.000	
Chinese	A	.272	10	.035	.802	10	.015	
	C1	.272	10	.035	.802	10	.015	
	C2	.322	30	.000	.754	30	.000	
	S	.375	30	.000	.721	30	.000	
English	A	.433	10	.000	.594	10	.000	
	C1	.381	10	.000	.640	10	.000	
	C2	.389	30	.000	.624	30	.000	
	S	.337	30	.000	.638	30	.000	

a. There are no valid cases for GPT_3 when Group2 = .000. Statistics cannot be computed for this level.	
b. Lilliefors Significance Correction	
c. There are no valid cases for GPT_4 when Group2 = .000. Statistics cannot be computed for this level.	
d. There are no valid cases for Chinese when Group2 = .000. Statistics cannot be computed for this level.	
e. There are no valid cases for English when Group2 = .000. Statistics cannot be computed for this level.	


GPT_3


Histograms


Stem-and-Leaf Plots


GPT_3 Stem-and-Leaf Plot for
Group2= A

 Frequency    Stem &  Leaf

     1.00 Extremes    (=<3)
      .00        0 .
     7.00        0 .  4444444
     2.00 Extremes    (>=5)

 Stem width:   10
 Each leaf:        1 case(s)


GPT_3 Stem-and-Leaf Plot for
Group2= C1

 Frequency    Stem &  Leaf

     1.00        3 .  0
      .00        3 .
     6.00        4 .  000000
      .00        4 .
     3.00        5 .  000

 Stem width:  1.0
 Each leaf:        1 case(s)


GPT_3 Stem-and-Leaf Plot for
Group2= C2

 Frequency    Stem &  Leaf

     1.00 Extremes    (=<2.0)
     2.00        3 .  00
      .00        3 .
    14.00        4 .  00000000000000
      .00        4 .
    13.00        5 .  0000000000000

 Stem width:  1.0
 Each leaf:        1 case(s)


GPT_3 Stem-and-Leaf Plot for
Group2= S

 Frequency    Stem &  Leaf

     2.00        3 .  00
      .00        3 .
    14.00        4 .  00000000000000
      .00        4 .
    14.00        5 .  00000000000000

 Stem width:  1.0
 Each leaf:        1 case(s)


Normal Q-Q Plots


Detrended Normal Q-Q Plots


GPT_4


Histograms


Stem-and-Leaf Plots


GPT_4 Stem-and-Leaf Plot for
Group2= A

 Frequency    Stem &  Leaf

     5.00        4 .  00000
      .00        4 .
     5.00        5 .  00000

 Stem width:  1.0
 Each leaf:        1 case(s)


GPT_4 Stem-and-Leaf Plot for
Group2= C1

 Frequency    Stem &  Leaf

     5.00        4 .  00000
      .00        4 .
     5.00        5 .  00000

 Stem width:  1.0
 Each leaf:        1 case(s)


GPT_4 Stem-and-Leaf Plot for
Group2= C2

 Frequency    Stem &  Leaf

    17.00        4 .  00000000000000000
      .00        4 .
      .00        4 .
      .00        4 .
      .00        4 .
    13.00        5 .  0000000000000

 Stem width:  1.0
 Each leaf:        1 case(s)


GPT_4 Stem-and-Leaf Plot for
Group2= S

 Frequency    Stem &  Leaf

    21.00        4 .  000000000000000000000
      .00        4 .
      .00        4 .
      .00        4 .
      .00        4 .
     9.00        5 .  000000000

 Stem width:  1.0
 Each leaf:        1 case(s)


Normal Q-Q Plots


Detrended Normal Q-Q Plots


Chinese


Histograms


Stem-and-Leaf Plots


Chinese Stem-and-Leaf Plot for
Group2= A

 Frequency    Stem &  Leaf

     1.00        3 .  0
      .00        3 .
     5.00        4 .  00000
      .00        4 .
     4.00        5 .  0000

 Stem width:  1.0
 Each leaf:        1 case(s)


Chinese Stem-and-Leaf Plot for
Group2= C1

 Frequency    Stem &  Leaf

     1.00        3 .  0
      .00        3 .
     5.00        4 .  00000
      .00        4 .
     4.00        5 .  0000

 Stem width:  1.0
 Each leaf:        1 case(s)


Chinese Stem-and-Leaf Plot for
Group2= C2

 Frequency    Stem &  Leaf

     1.00 Extremes    (=<2.0)
     2.00        3 .  00
      .00        3 .
    19.00        4 .  0000000000000000000
      .00        4 .
     8.00        5 .  00000000

 Stem width:  1.0
 Each leaf:        1 case(s)


Chinese Stem-and-Leaf Plot for
Group2= S

 Frequency    Stem &  Leaf

     2.00        3 .  00
      .00        3 .
    20.00        4 .  00000000000000000000
      .00        4 .
     8.00        5 .  00000000

 Stem width:  1.0
 Each leaf:        1 case(s)


Normal Q-Q Plots


Detrended Normal Q-Q Plots


English


Histograms


Stem-and-Leaf Plots


English Stem-and-Leaf Plot for
Group2= A

 Frequency    Stem &  Leaf

     7.00        4 .  0000000
      .00        4 .
     3.00        5 .  000

 Stem width:  1.0
 Each leaf:        1 case(s)


English Stem-and-Leaf Plot for
Group2= C1

 Frequency    Stem &  Leaf

     6.00        4 .  000000
      .00        4 .
     4.00        5 .  0000

 Stem width:  1.0
 Each leaf:        1 case(s)


English Stem-and-Leaf Plot for
Group2= C2

 Frequency    Stem &  Leaf

    12.00        4 .  000000000000
      .00        4 .
      .00        4 .
      .00        4 .
      .00        4 .
    18.00        5 .  000000000000000000

 Stem width:  1.0
 Each leaf:        1 case(s)


English Stem-and-Leaf Plot for
Group2= S

 Frequency    Stem &  Leaf

    15.00        4 .  000000000000000
      .00        4 .
      .00        4 .
      .00        4 .
      .00        4 .
    15.00        5 .  000000000000000

 Stem width:  1.0
 Each leaf:        1 case(s)


Normal Q-Q Plots


Detrended Normal Q-Q Plots
