# Supplementary material for: Assessing the Role of the Generative Pretrained Transformer (GPT) in Alzheimer’s Disease Management: Comparative Study of Neurologist- and Artificial Intelligence–Generated Responses
Source: J Med Internet Res. 2024 Oct 31;26:e51095. doi: 10.2196/51095 (PMC11565080; doi:10.2196/51095)
Supplement: Multimedia Appendix 5 [file jmir_v26i1e51095_app5.doc]

Case Processing Summary	
	Group	Cases	
		Valid	Missing	Total	
		N	Percent	N	Percent	N	Percent	
GPT	C	60	100.0%	0	0.0%	60	100.0%	
	S	60	100.0%	0	0.0%	60	100.0%	
	U	40	100.0%	0	0.0%	40	100.0%	
Human	C	60	100.0%	0	0.0%	60	100.0%	
	S	60	100.0%	0	0.0%	60	100.0%	
	U	40	100.0%	0	0.0%	40	100.0%	


Descriptivesa,b	
	Group	Statistic	Std. Error	
GPT	C	Mean	3.983	.0732	
		95% Confidence Interval for Mean	Lower Bound	3.837		
			Upper Bound	4.130		
		5% Trimmed Mean	3.981		
		Median	4.000		
		Variance	.322		
		Std. Deviation	.5672		
		Minimum	3.0		
		Maximum	5.0		
		Range	2.0		
		Interquartile Range	.0		
		Skewness	-.005	.309	
		Kurtosis	.279	.608	
	S	Mean	3.733	.0784	
		95% Confidence Interval for Mean	Lower Bound	3.577		
			Upper Bound	3.890		
		5% Trimmed Mean	3.704		
		Median	4.000		
		Variance	.368		
		Std. Deviation	.6069		
		Minimum	3.0		
		Maximum	5.0		
		Range	2.0		
		Interquartile Range	1.0		
		Skewness	.198	.309	
		Kurtosis	-.515	.608	
	U	Mean	3.900	.0784	
		95% Confidence Interval for Mean	Lower Bound	3.741		
			Upper Bound	4.059		
		5% Trimmed Mean	3.889		
		Median	4.000		
		Variance	.246		
		Std. Deviation	.4961		
		Minimum	3.0		
		Maximum	5.0		
		Range	2.0		
		Interquartile Range	.0		
		Skewness	-.239	.374	
		Kurtosis	1.191	.733	
Human	C	Mean	3.917	.0959	
		95% Confidence Interval for Mean	Lower Bound	3.725		
			Upper Bound	4.109		
		5% Trimmed Mean	3.926		
		Median	4.000		
		Variance	.552		
		Std. Deviation	.7431		
		Minimum	2.0		
		Maximum	5.0		
		Range	3.0		
		Interquartile Range	1.0		
		Skewness	-.120	.309	
		Kurtosis	-.532	.608	
	S	Mean	3.733	.1137	
		95% Confidence Interval for Mean	Lower Bound	3.506		
			Upper Bound	3.961		
		5% Trimmed Mean	3.778		
		Median	4.000		
		Variance	.775		
		Std. Deviation	.8804		
		Minimum	1.0		
		Maximum	5.0		
		Range	4.0		
		Interquartile Range	1.0		
		Skewness	-.829	.309	
		Kurtosis	.831	.608	
	U	Mean	3.975	.1043	
		95% Confidence Interval for Mean	Lower Bound	3.764		
			Upper Bound	4.186		
		5% Trimmed Mean	3.972		
		Median	4.000		
		Variance	.435		
		Std. Deviation	.6597		
		Minimum	3.0		
		Maximum	5.0		
		Range	2.0		
		Interquartile Range	.0		
		Skewness	.026	.374	
		Kurtosis	-.567	.733	

a. There are no valid cases for GPT when Group = .000. Statistics cannot be computed for this level.	
b. There are no valid cases for Human when Group = .000. Statistics cannot be computed for this level.	


Tests of Normalitya,c	
	Group	Kolmogorov-Smirnovb	Shapiro-Wilk	
		Statistic	df	Sig.	Statistic	df	Sig.	
GPT	C	.345	60	.000	.738	60	.000	
	S	.320	60	.000	.758	60	.000	
	U	.405	40	.000	.669	40	.000	
Human	C	.261	60	.000	.838	60	.000	
	S	.319	60	.000	.838	60	.000	
	U	.290	40	.000	.794	40	.000	

a. There are no valid cases for GPT when Group = .000. Statistics cannot be computed for this level.	
b. Lilliefors Significance Correction	
c. There are no valid cases for Human when Group = .000. Statistics cannot be computed for this level.	


GPT


Histograms


Stem-and-Leaf Plots


GPT Stem-and-Leaf Plot for
Group= C

 Frequency    Stem &  Leaf

    10.00 Extremes    (=<3)
      .00        0 .
    41.00        0 .  44444444444444444444444444444444444444444
     9.00 Extremes    (>=5)

 Stem width:   10
 Each leaf:        1 case(s)


GPT Stem-and-Leaf Plot for
Group= S

 Frequency    Stem &  Leaf

    21.00        3 .  000000000000000000000
      .00        3 .
    34.00        4 .  0000000000000000000000000000000000
      .00        4 .
     5.00        5 .  00000

 Stem width:  1.0
 Each leaf:        1 case(s)


GPT Stem-and-Leaf Plot for
Group= U

 Frequency    Stem &  Leaf

     7.00 Extremes    (=<3)
      .00        0 .
    30.00        0 .  444444444444444444444444444444
     3.00 Extremes    (>=5)

 Stem width:   10
 Each leaf:        1 case(s)


Normal Q-Q Plots


Detrended Normal Q-Q Plots


Human


Histograms


Stem-and-Leaf Plots


Human Stem-and-Leaf Plot for
Group= C

 Frequency    Stem &  Leaf

     1.00        2 .  0
      .00        2 .
    16.00        3 .  0000000000000000
      .00        3 .
    30.00        4 .  000000000000000000000000000000
      .00        4 .
    13.00        5 .  0000000000000

 Stem width:  1.0
 Each leaf:        1 case(s)


Human Stem-and-Leaf Plot for
Group= S

 Frequency    Stem &  Leaf

     1.00 Extremes    (=<1.0)
     5.00        2 .  00000
      .00        2 .
    12.00        3 .  000000000000
      .00        3 .
    33.00        4 .  000000000000000000000000000000000
      .00        4 .
     9.00        5 .  000000000

 Stem width:  1.0
 Each leaf:        1 case(s)


Human Stem-and-Leaf Plot for
Group= U

 Frequency    Stem &  Leaf

     9.00 Extremes    (=<3)
      .00        0 .
    23.00        0 .  44444444444444444444444
     8.00 Extremes    (>=5)

 Stem width:   10
 Each leaf:        1 case(s)


Normal Q-Q Plots


Detrended Normal Q-Q Plots


Case Processing Summary	
	Group2	Cases	
		Valid	Missing	Total	
		N	Percent	N	Percent	N	Percent	
GPT_3	C	30	100.0%	0	0.0%	30	100.0%	
	S	30	100.0%	0	0.0%	30	100.0%	
	U	20	100.0%	0	0.0%	20	100.0%	
GPT_4	C	30	100.0%	0	0.0%	30	100.0%	
	S	30	100.0%	0	0.0%	30	100.0%	
	U	20	100.0%	0	0.0%	20	100.0%	
Chinese	C	30	100.0%	0	0.0%	30	100.0%	
	S	30	100.0%	0	0.0%	30	100.0%	
	U	20	100.0%	0	0.0%	20	100.0%	
English	C	30	100.0%	0	0.0%	30	100.0%	
	S	30	100.0%	0	0.0%	30	100.0%	
	U	20	100.0%	0	0.0%	20	100.0%	


Descriptivesa,b,c,d	
	Group2	Statistic	Std. Error	
GPT_3	C	Mean	4.067	.1065	
		95% Confidence Interval for Mean	Lower Bound	3.849		
			Upper Bound	4.284		
		5% Trimmed Mean	4.074		
		Median	4.000		
		Variance	.340		
		Std. Deviation	.5833		
		Minimum	3.0		
		Maximum	5.0		
		Range	2.0		
		Interquartile Range	.0		
		Skewness	.003	.427	
		Kurtosis	.229	.833	
	S	Mean	3.667	.1107	
		95% Confidence Interval for Mean	Lower Bound	3.440		
			Upper Bound	3.893		
		5% Trimmed Mean	3.630		
		Median	4.000		
		Variance	.368		
		Std. Deviation	.6065		
		Minimum	3.0		
		Maximum	5.0		
		Range	2.0		
		Interquartile Range	1.0		
		Skewness	.294	.427	
		Kurtosis	-.550	.833	
	U	Mean	3.800	.1170	
		95% Confidence Interval for Mean	Lower Bound	3.555		
			Upper Bound	4.045		
		5% Trimmed Mean	3.778		
		Median	4.000		
		Variance	.274		
		Std. Deviation	.5231		
		Minimum	3.0		
		Maximum	5.0		
		Range	2.0		
		Interquartile Range	.8		
		Skewness	-.294	.512	
		Kurtosis	.457	.992	
GPT_4	C	Mean	3.900	.1000	
		95% Confidence Interval for Mean	Lower Bound	3.695		
			Upper Bound	4.105		
		5% Trimmed Mean	3.889		
		Median	4.000		
		Variance	.300		
		Std. Deviation	.5477		
		Minimum	3.0		
		Maximum	5.0		
		Range	2.0		
		Interquartile Range	.0		
		Skewness	-.081	.427	
		Kurtosis	.589	.833	
	S	Mean	3.800	.1114	
		95% Confidence Interval for Mean	Lower Bound	3.572		
			Upper Bound	4.028		
		5% Trimmed Mean	3.778		
		Median	4.000		
		Variance	.372		
		Std. Deviation	.6103		
		Minimum	3.0		
		Maximum	5.0		
		Range	2.0		
		Interquartile Range	1.0		
		Skewness	.117	.427	
		Kurtosis	-.298	.833	
	U	Mean	4.000	.1026	
		95% Confidence Interval for Mean	Lower Bound	3.785		
			Upper Bound	4.215		
		5% Trimmed Mean	4.000		
		Median	4.000		
		Variance	.211		
		Std. Deviation	.4588		
		Minimum	3.0		
		Maximum	5.0		
		Range	2.0		
		Interquartile Range	.0		
		Skewness	.000	.512	
		Kurtosis	2.980	.992	
Chinese	C	Mean	4.067	.1065	
		95% Confidence Interval for Mean	Lower Bound	3.849		
			Upper Bound	4.284		
		5% Trimmed Mean	4.074		
		Median	4.000		
		Variance	.340		
		Std. Deviation	.5833		
		Minimum	3.0		
		Maximum	5.0		
		Range	2.0		
		Interquartile Range	.0		
		Skewness	.003	.427	
		Kurtosis	.229	.833	
	S	Mean	3.633	.1123	
		95% Confidence Interval for Mean	Lower Bound	3.404		
			Upper Bound	3.863		
		5% Trimmed Mean	3.593		
		Median	4.000		
		Variance	.378		
		Std. Deviation	.6149		
		Minimum	3.0		
		Maximum	5.0		
		Range	2.0		
		Interquartile Range	1.0		
		Skewness	.404	.427	
		Kurtosis	-.567	.833	
	U	Mean	3.850	.1313	
		95% Confidence Interval for Mean	Lower Bound	3.575		
			Upper Bound	4.125		
		5% Trimmed Mean	3.833		
		Median	4.000		
		Variance	.345		
		Std. Deviation	.5871		
		Minimum	3.0		
		Maximum	5.0		
		Range	2.0		
		Interquartile Range	.8		
		Skewness	.004	.512	
		Kurtosis	.178	.992	
English	C	Mean	3.900	.1000	
		95% Confidence Interval for Mean	Lower Bound	3.695		
			Upper Bound	4.105		
		5% Trimmed Mean	3.889		
		Median	4.000		
		Variance	.300		
		Std. Deviation	.5477		
		Minimum	3.0		
		Maximum	5.0		
		Range	2.0		
		Interquartile Range	.0		
		Skewness	-.081	.427	
		Kurtosis	.589	.833	
	S	Mean	3.833	.1081	
		95% Confidence Interval for Mean	Lower Bound	3.612		
			Upper Bound	4.054		
		5% Trimmed Mean	3.815		
		Median	4.000		
		Variance	.351		
		Std. Deviation	.5921		
		Minimum	3.0		
		Maximum	5.0		
		Range	2.0		
		Interquartile Range	1.0		
		Skewness	.040	.427	
		Kurtosis	-.082	.833	
	U	Mean	3.950	.0881	
		95% Confidence Interval for Mean	Lower Bound	3.766		
			Upper Bound	4.134		
		5% Trimmed Mean	3.944		
		Median	4.000		
		Variance	.155		
		Std. Deviation	.3940		
		Minimum	3.0		
		Maximum	5.0		
		Range	2.0		
		Interquartile Range	.0		
		Skewness	-.531	.512	
		Kurtosis	4.985	.992	

a. There are no valid cases for GPT_3 when Group2 = .000. Statistics cannot be computed for this level.	
b. There are no valid cases for GPT_4 when Group2 = .000. Statistics cannot be computed for this level.	
c. There are no valid cases for Chinese when Group2 = .000. Statistics cannot be computed for this level.	
d. There are no valid cases for English when Group2 = .000. Statistics cannot be computed for this level.	


Tests of Normalitya,c,d,e	
	Group2	Kolmogorov-Smirnovb	Shapiro-Wilk	
		Statistic	df	Sig.	Statistic	df	Sig.	
GPT_3	C	.345	30	.000	.750	30	.000	
	S	.309	30	.000	.754	30	.000	
	U	.399	20	.000	.695	20	.000	
GPT_4	C	.372	30	.000	.721	30	.000	
	S	.328	30	.000	.765	30	.000	
	U	.400	20	.000	.632	20	.000	
Chinese	C	.345	30	.000	.750	30	.000	
	S	.291	30	.000	.753	30	.000	
	U	.351	20	.000	.754	20	.000	
English	C	.372	30	.000	.721	30	.000	
	S	.344	30	.000	.755	30	.000	
	U	.450	20	.000	.545	20	.000	

a. There are no valid cases for GPT_3 when Group2 = .000. Statistics cannot be computed for this level.	
b. Lilliefors Significance Correction	
c. There are no valid cases for GPT_4 when Group2 = .000. Statistics cannot be computed for this level.	
d. There are no valid cases for Chinese when Group2 = .000. Statistics cannot be computed for this level.	
e. There are no valid cases for English when Group2 = .000. Statistics cannot be computed for this level.	


GPT_3


Histograms


Stem-and-Leaf Plots


GPT_3 Stem-and-Leaf Plot for
Group2= C

 Frequency    Stem &  Leaf

     4.00 Extremes    (=<3)
      .00        0 .
    20.00        0 .  44444444444444444444
     6.00 Extremes    (>=5)

 Stem width:   10
 Each leaf:        1 case(s)


GPT_3 Stem-and-Leaf Plot for
Group2= S

 Frequency    Stem &  Leaf

    12.00        3 .  000000000000
      .00        3 .
    16.00        4 .  0000000000000000
      .00        4 .
     2.00        5 .  00

 Stem width:  1.0
 Each leaf:        1 case(s)


GPT_3 Stem-and-Leaf Plot for
Group2= U

 Frequency    Stem &  Leaf

     5.00        3 .  00000
      .00        3 .
    14.00        4 .  00000000000000
     1.00 Extremes    (>=5.0)

 Stem width:  1.0
 Each leaf:        1 case(s)


Normal Q-Q Plots


Detrended Normal Q-Q Plots


GPT_4


Histograms


Stem-and-Leaf Plots


GPT_4 Stem-and-Leaf Plot for
Group2= C

 Frequency    Stem &  Leaf

     6.00 Extremes    (=<3)
      .00        0 .
    21.00        0 .  444444444444444444444
     3.00 Extremes    (>=5)

 Stem width:   10
 Each leaf:        1 case(s)


GPT_4 Stem-and-Leaf Plot for
Group2= S

 Frequency    Stem &  Leaf

     9.00        3 .  000000000
      .00        3 .
    18.00        4 .  000000000000000000
      .00        4 .
     3.00        5 .  000

 Stem width:  1.0
 Each leaf:        1 case(s)


GPT_4 Stem-and-Leaf Plot for
Group2= U

 Frequency    Stem &  Leaf

     2.00 Extremes    (=<3)
      .00        0 .
    16.00        0 .  4444444444444444
     2.00 Extremes    (>=5)

 Stem width:   10
 Each leaf:        1 case(s)


Normal Q-Q Plots


Detrended Normal Q-Q Plots


Chinese


Histograms


Stem-and-Leaf Plots


Chinese Stem-and-Leaf Plot for
Group2= C

 Frequency    Stem &  Leaf

     4.00 Extremes    (=<3)
      .00        0 .
    20.00        0 .  44444444444444444444
     6.00 Extremes    (>=5)

 Stem width:   10
 Each leaf:        1 case(s)


Chinese Stem-and-Leaf Plot for
Group2= S

 Frequency    Stem &  Leaf

    13.00        3 .  0000000000000
      .00        3 .
    15.00        4 .  000000000000000
      .00        4 .
     2.00        5 .  00

 Stem width:  1.0
 Each leaf:        1 case(s)


Chinese Stem-and-Leaf Plot for
Group2= U

 Frequency    Stem &  Leaf

     5.00        3 .  00000
      .00        3 .
    13.00        4 .  0000000000000
     2.00 Extremes    (>=5.0)

 Stem width:  1.0
 Each leaf:        1 case(s)


Normal Q-Q Plots


Detrended Normal Q-Q Plots


English


Histograms


Stem-and-Leaf Plots


English Stem-and-Leaf Plot for
Group2= C

 Frequency    Stem &  Leaf

     6.00 Extremes    (=<3)
      .00        0 .
    21.00        0 .  444444444444444444444
     3.00 Extremes    (>=5)

 Stem width:   10
 Each leaf:        1 case(s)


English Stem-and-Leaf Plot for
Group2= S

 Frequency    Stem &  Leaf

     8.00        3 .  00000000
      .00        3 .
    19.00        4 .  0000000000000000000
      .00        4 .
     3.00        5 .  000

 Stem width:  1.0
 Each leaf:        1 case(s)


English Stem-and-Leaf Plot for
Group2= U

 Frequency    Stem &  Leaf

     2.00 Extremes    (=<3)
      .00        0 .
    17.00        0 .  44444444444444444
     1.00 Extremes    (>=5)

 Stem width:   10
 Each leaf:        1 case(s)


Normal Q-Q Plots


Detrended Normal Q-Q Plots
